# Eco-evolutionary feedbacks drive the co-occurrence of restriction-modification systems and antimicrobial resistance genes in bacteria

**DOI:** 10.1371/journal.pbio.3003842

**Published:** 2026-06-15

**Authors:** Joseph Westley, Paritosh Bedekar, Elizabeth Pursey, Mark D. Szczelkun, Mario Recker, Stineke van Houte, Edze R. Westra

**Affiliations:** 1 Centre for Ecology and Conservation, Faculty of Environment, Science, and Economy, University of Exeter, Penryn, United Kingdom; 2 DNA-Protein Interactions Unit, School of Biochemistry, Faculty of Life Sciences, University of Bristol, Bristol, United Kingdom; 3 Institute for Tropical Medicine, University Hospital Tübingen, Tübingen, Germany; London School of Hygiene & Tropical Medicine, UNITED KINGDOM OF GREAT BRITAIN AND NORTHERN IRELAND

## Abstract

Bacterial pathogens commonly become drug resistant via horizontal acquisition of antimicrobial resistance genes (ARGs), which are often encoded on mobile genetic elements (MGEs). Although bacterial defence systems are typically considered barriers to horizontal gene transfer (HGT), previous studies revealed that bacteria with more restriction-modification (RM) systems (the most abundant bacterial defences) frequently carry more MGEs. It was suggested that this counterintuitive relationship might result from stronger selection for RM systems when exposure to costly MGEs increases. Here, we test this hypothesis using a combination of modeling and bioinformatics analysis of >40,000 bacterial genomes to better understand how eco-evolutionary feedbacks between selection for RM and acquisition of MGEs shape bacterial genome evolution. Our model predicts negative associations between HGT and RM, but only if RM diversity is high. By contrast, at low RM diversity, eco-evolutionary feedbacks drive the emergence of positive associations between HGT and RM. Consistent with these predictions, we identified negative relationships between acquired ARG counts and RM counts across species but positive relationships within individual species. Collectively, our work helps to understand how RM systems shape patterns of HGT of ARGs, which may offer opportunities for targeted surveillance of strains at higher risk of horizontally acquiring novel drug resistance alleles.

## Abstract 2

Bacteria acquire antimicrobial resistance genes via horizontal gene transfer despite defense systems that should block it. This study shows that restriction-modification (RM) systems’ diversity determines this relationship, with high diversity limiting gene transfer and low diversity enabling eco-evolutionary feedbacks that promote both RM investment and gene acquisition.

## Significance statement

Previous research shows positive associations between bacterial restriction-modification (RM) systems and mobile genes, despite RM being well-evidenced as a barrier to mobile gene acquisition. Eco-evolutionary feedbacks have been hypothesized to drive this; higher HGT exposure will select for greater RM investment, while still leading to increased mobile gene acquisition. By formalizing this hypothesis into a mathematical model and analyzing bacterial genomes, we reveal novel insights into how the relationship between RM and mobile antimicrobial resistance genes (ARGs) depends on ecological variables. This study illustrates that to better comprehend how defence systems regulate gene transfer in natural communities, it is essential to consider both their mechanisms and the complex eco-evolutionary dynamics at play.

## Introduction

It is difficult to overstate the urgency of the antimicrobial resistance crisis, with global clinical and agricultural use of antimicrobials on the increase [1,2], and predictions of 10 million deaths annually from drug-resistant infections for 2050 [3]. The recent growth in the prevalence of multi-drug-resistant pathogens is to a large extent driven by horizontal gene transfer (HGT) of antimicrobial resistance genes (ARGs) by mobile genetic elements (MGEs) [4–7]. MGEs are pieces of DNA that specialize in moving between and within genomes, and include plasmids, transposons, integrative and conjugative elements and bacteriophages (phages).

Prokaryotes have evolved a diverse arsenal of defence systems to protect them against infection from MGEs [8]. It remains unclear how important these defence systems are in controlling how ARGs are horizontally transferred. Of the > 100 microbial defence systems discovered to date [9], the most ubiquitous are restriction-modification (RM) systems [10]. Understanding the role RM plays in regulating HGT of ARGs is essential to better understand how multi-drug resistance is acquired, which taxa may be important sources of ARGs, and which taxa may be more likely to acquire them.

RM systems perform two main functions: the methylation of self-DNA at specific target sites (modification), and the degradation of DNA that is unmethylated at these sites (restriction) [11]. Through these two processes, RM systems allow discrimination between self and non-self-DNA and cleavage of the latter, providing the cell with innate immunity against unmethylated MGEs [11–13]. Importantly, if a cell donating an MGE (donor) possesses an RM system that methylates the same site as the RM system of a cell receiving the MGE (recipient), the recipient’s RM system will not cleave the MGE. This is because the donor’s own methyltransferase will methylate the MGE at the target site, protecting the MGE from cleavage by the respective endonuclease, allowing the MGE to escape the recipient’s RM system upon transfer. De novo escape can also occur at low frequencies when the recipient possesses an RM system the donor does not, if the MGE is methylated by the recipient’s own methyltransferases before the respective endonucleases can degrade the MGE.

Experimental studies have demonstrated that RM systems act as a barrier to phage and plasmid infection, albeit imperfectly [14–27]. Moreover, the role of RM as a barrier to MGE infection is further supported by signatures of restriction avoidance in MGEs. Specifically, the target motifs that RM systems recognize are short (typically 4–8 bp for Type II and Type III systems) [28] but frequencies in many MGEs (especially small plasmids and phages) are often below what would be expected by chance [29–31] or are in orientations that preclude cleavage [32]. Additionally, anti-restriction systems have been observed in both phages [33–35] and plasmids [36–38], and function to neutralize their host’s RM system(s) [11,39]. Furthermore, HGT of ARGs between phylogroups (genetically distinct lineages) in methicillin-resistant *Staphylococcus aureus* (MRSA) has been proposed to be regulated by between-phylogroup variation in RM systems [29].

Given this extensive evidence that RM systems can pose a barrier to the acquisition of MGEs, intuitively one would predict that lineages associated with more RM systems would carry fewer horizontally acquired genes. However, a bioinformatic study of >800 genomes that investigated the relationship between the presence of RM systems and gene gain via HGT and homologous recombination demonstrated that lineages associated with more RM systems were also associated with greater gene gain [40].

Several potential explanations for these positive associations between RM and MGE content have been put forward, but none of these has been rigorously tested. First, it has been suggested that positive associations may arise as a result of eco-evolutionary feedbacks: as exposure to costly MGEs increases, so will selection for investment into RM systems [40]. A second explanation is that MGEs may carry RM systems (genetic linkage) [41–44], thus resulting in positive associations. Thirdly, as RM systems can be horizontally acquired [41–44], it is possible lineages acquired MGEs before they acquired RM, and then these lineages carrying both adaptive MGE-borne genes [45] as well as genome defences (RM) may be under strong positive selection.

To identify the potential mechanisms that drive previously reported positive associations between RM and MGEs, we first developed a mathematical model of the eco-evolutionary dynamics of bacterial populations carrying RM systems exposed to MGE infection. This model predicts conditions that drive either positive or negative associations between RM and MGE content, depending on the population-level diversity of RM systems. Next, we tested model predictions through in-depth bioinformatic analyses of >40,000 publicly available genomes of key bacterial pathogens. Pathogenic species were chosen due to their propensity to acquire novel ARGs through HGT making them especially clinically relevant for studying the interplay between RM systems, MGEs, and antibiotic resistance. Due to the greater scale of our dataset compared with previous research [40], we were able to conduct both between-species and phylogenetically controlled within-species analyses.

Combining these modeling and bioinformatics methods, we conclude that eco-evolutionary feedbacks between MGE exposure and investment in genome defence form the most parsimonious explanation for the observed positive relationships between RM and MGE content in bacterial genomes. We propose that this information can be used to identify species networks where HGT is potentially less restricted by RM, which may offer opportunities for targeted intervention strategies to limit the spread of AMR.

## Results and discussion

### Positive associations between RM and MGEs arise when population-level RM diversity is low

To formalize the hypothesis that eco-evolutionary feedbacks can drive positive associations between RM and MGE content in bacterial genomes, we utilized a modeling approach (see Materials and methods, “Mathematical modeling”). We competed three sub-populations with varying levels of investment into RM: zero, low, and high (see Table 2 for parameter values including selection coefficients of sub-populations). We allowed the gain of up to five MGEs, with higher investment into RM resulting in a reduced likelihood of MGE acquisition. We varied the rates of HGT as well as the diversity of RM systems in the population, as we predict RM becomes a weaker barrier to HGT when the probability that donors and recipients share the same RM specificity increases [46,47], thus increasing the probability of gene exchange between lineages.

**Fig 1:**
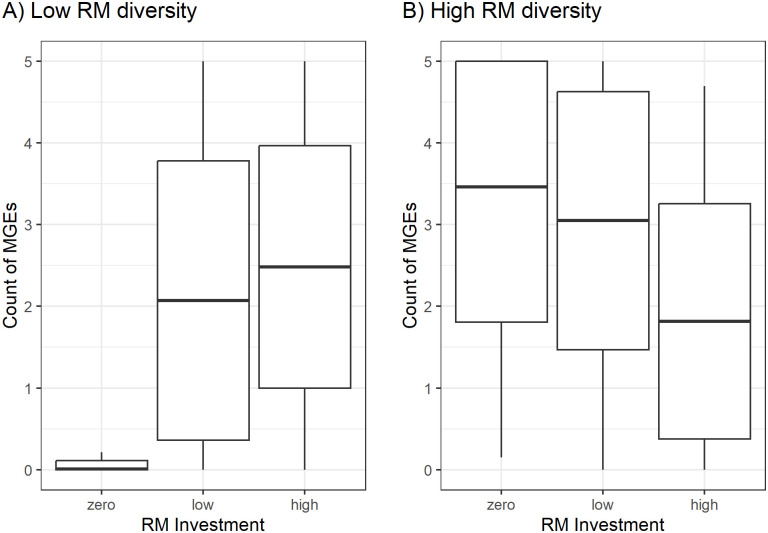
The average count of MGEs per individual carried by sub-populations with different levels of RM investment once equilibrium had been reached. Simulations when population-level RM diversity is either **A)** low or **B)** high. The central horizontal line indicates the mean, the boxes indicate ± 1 standard deviation (SD) from the mean, the whiskers denote ±2 SD from the mean. Here, data from all simulations across the full range of relative HGT rates are combined. The data underlying this Figure are available via Zenodo: https://doi.org/10.5281/zenodo.19387437.

In simulations where population-level RM diversity was low, bacteria with high RM investment also had a higher mean count of MGEs (Fig 1A) once equilibrium had been reached, whereas a negative relationship between RM investment and mean MGE count was observed when the simulated population-level RM diversity was higher (Fig 1B). To ensure robustness of these results, we ran simulations with varying RM investment costs and varying MGE costs. This revealed qualitatively similar outcomes unless RM diversity was low and carrying these systems was more costly than being infected by MGEs (S1 Fig).

Collectively, these simulations suggest high exposure to costly MGEs can, through changes in allele frequencies, drive both an increased investment into RM-mediated genome defence as well as an increase in the acquisition of mobile genes, provided that the population-level diversity of RM systems is low. This is likely because RM systems are an imperfect barrier, and once the barrier is crossed, methylated MGEs will be able to infect other bacteria that carry the same RM specificity, but not bacteria with a different RM specificity. In other words, RM diversity impacts the prevalence of routes for gene exchange between lineages with matching RM target sites (as described by Oliveira *and colleagues* [40]), and will therefore impact the directionality of the relationship between MGE acquisition and RM.

To elucidate whether positive associations between RM investment and MGE carriage at low RM diversity is dependent upon the efficacy of the RM system, we varied the fold reduction in MGE uptake the high investment RM system provides. We consistently observed positive associations between RM investment and MGE carriage, even at the highest modeled efficacies (S2 Fig). Again, this is because even the most stringent RM systems function only against non-methylated MGEs, so even if escape is very rare, if it can happen at least once, MGEs bearing the protective methylation signature freely spread through the RM sub-population.

In addition to the mean count of MGEs carried within each sub-population, we also investigated how the size of sub-populations with different RM investment strategies, varied with RM and MGE costs, and RM diversity once equilibrium had been reached, (S3 Fig). For low RM diversity, high RM investment sub-populations tended to outcompete low and zero investment sub-populations when RM investment was less costly and MGE carriage was more costly. However, as RM investment became more costly and MGE carriage became less costly, the subpopulation with low investment in RM systems tended to be the dominant one. Zero investment into RM always had the lowest cell counts at equilibria, except for the simulations with highest and lowest extremes of RM and MGE costs, respectively. In the high RM diversity simulations, there was a shift towards the low RM investment sub-populations being dominant across a broader range of parameters than in low RM diversity simulations, and zero investment sub-populations also increased in relative abundance across simulations. The relationship between RM investment and fitness did, however, shift with RM and MGE cost in a similar manner to the low diversity simulations; as MGEs became more costly and RM investment became less costly, the high RM investment sub-population increased in relative abundance.

Our findings that across simulations, high RM investment sub-populations were more competitive when RM carriage was less costly, and MGE carriage was more costly, is intuitive. Furthermore, high RM investment sub-populations becoming less competitive when RM diversity is high is consistent with a reduction in the force of infection by MGEs (due to each sub-population being exposed to infection by fewer methylated MGEs), reducing selection for high investment into defence.

### Identification of RM systems and mobile ARGs in a large-scale genomic dataset

To test our model predictions, we utilized bioinformatic analyses to investigate the association between RM systems and horizontally acquired ARGs, leveraging the wealth of publicly available genomic data for bacterial pathogens and established tools for ARG annotation.

We searched a total of 40,181 genomes from 14 bacterial pathogen species from NCBI’s *RefSeq* database, for defence systems and acquired ARGs (Table 1). This 50-fold increase in genomic dataset size, compared with previous analyses of the relationship between RM and mobile genes [40], allowed us to conduct between-species and phylogenetically controlled within-species analyses.

**Table 1:** Count of genomes analyzed, RM systems found, and ARGs found, for each species. The data underlying this Table are available via Zenodo: https://doi.org/10.5281/zenodo.19387437.

Species	Genomes	Type I RM	Type II RM	Type III RM	Type IV RM	Total RM	ARGs
*Pseudomonas aeruginosa*	4,751	1,991	1,132	370	51	3,544	33,826
*Acinetobacter baumannii*	3,986	574	135	88	1	798	41,996
*Enterococcus faecium*	1,947	297	154	94	1	546	22,258
*Streptococcus pyogenes*	1,753	1,687	72	0	0	1,759	782
*Neisseria gonorrhoeae*	622	498	3,253	579	0	4,330	97
*Campylobacter jejuni*	1,478	577	2,530	267	0	3,374	1,956
*Helicobacter pylori*	1,512	1,652	5,566	1,479	0	8,697	35
*Shigella flexneri*	415	407	2	0	2	411	3,309
*Shigella sonnei*	994	30	982	15	8	1,035	6,734
*Clostridioides difficile*	1,717	1,337	459	4	8	1,808	10,329
*Klebsiella pneumoniae*	5,184	2,826	696	882	186	4,590	37,306
*Staphylococcus aureus*	4,669	692	1,342	66	3,438	5,538	27,558
*Mycobacterium tuberculosis*	5,241	0	7,875	0	9,348	17,223	12,388
*Salmonella enterica*	5,912	4,503	773	5,835	2,581	13,692	9,804
*Total*	40,181	17,071	24,971	9,679	15,624	67,345	208,378

Across our dataset, we observed systems from over 141 distinct defence system types, as defined by PADLOC, a tool that identifies bacterial defence systems in sequence data. We grouped all functionally similar systems, and of the resulting 41 distinct groups, we found that RM systems were the most abundant type of defence system, with 67,345 complete systems observed (Table 1, S4 Fig). Here, we further improve upon past work by including all types of RM systems in our analyses, rather than just Type II [40]. The average per-genome counts of RM systems varied considerably across species, from 0.20 in *Acinetobacter baumannii* to 6.96 in *Neisseria gonorrhoeae*. For the majority of species, the most abundant RM systems belonged to either Type I or Type II, with Type III and IV systems being less frequent (Table 1), a finding consistent with previous literature [8]. There were, however, two outlier species in this regard; over half of all identified Type III systems were observed in *Salmonella enterica*, and over half of all identified Type IV systems were observed in *Mycobacterium tuberculosis* (Table 1). In *Salmonella enterica*, the high prevalence of Type III systems is consistent with previous work suggesting that these systems were acquired early in the species’ evolutionary history, and subsequently became widespread across lineages [48]. In *Mycobacterium tuberculosis*, the enrichment of Type IV systems that cleave methylated DNA, together with relatively high counts of Type II systems that target unmethylated sites, may reflect a long-term arms race with phages or parasitic MGEs, in which Type II systems select for phage-encoded methyltransferases [49], and Type IV systems are then selected to counter these methylated invaders.

To assess whether annotated RM systems are functional, we validated our detection pipeline using genomes with independent evidence of epigenetic modification. Across 584 genomes from 14 species (including all study species except *M. tuberculosis*, due to a lack of available data, but additionally including *E. coli* to increase the validation dataset size), the per-genome count of RM systems (Types I, II, and III) generally closely aligned with the per-genome count of unique methylation patterns (Table A in S1 Appendix). A Bland–Altman analysis demonstrated strong agreement between these measures, with 95.04% of genomes falling within the limits of agreement (LOA) (S5A Fig). Consistent with this, the total number of RM systems per genome was strongly positively correlated with the number of unique methylation patterns (*R* = 0.67, *P* < 0.001; S5B Fig), a relationship that persisted when stratified by RM type (Type I: *R* = 0.36; Type II: *R* = 0.58; Type III: *R* = 0.38; all *P* < 0.001; S5C Fig). As expected, no correlation was observed for Type IV RM systems, which do not methylate host DNA (*R* = −0.12, *P* = 0.16).

Furthermore, we show that for 12 of 14 species, there is no significant difference between the count of annotated RM systems, and the count of unique methylation patterns (S5D Fig, Table A in S1 Appendix). Despite general agreement between these measures, two species showed discrepancies. *N. gonorrhoeae*, which also made up the vast majority of genomes that fell outside of the LOA in the Bland-Altman analysis, had more annotated RM systems than detected methylation patterns per genome, which likely reflects the high prevalence of phase-variable methyltransferases in this species [50,51], which can be reversibly inactivated. *S. aureus* had on average fewer Types I, II, and III RM systems than unique methylation patterns per genome, suggestive of this species carrying more methyltransferases with unique specificities than we are assigning to functional RM systems. Nevertheless, the direction of this result means we can be confident that we are not overestimating the count of functional RM systems in *S. aureus*. Together, our validation of RM functionality using epigenetic data indicates general agreement between annotated RM system counts and methylation patterns across most species.

Across all genomes, we also identified 208,378 acquired ARGs. Again, we observed considerable variability in the average counts of ARGs per genome between-species, from 0.02 in *Helicobacter pylori* to 10.54 in *Acinetobacter baumannii* (S6 Fig).

#### Species with more RM systems have fewer acquired ARGs.

**Fig 2:**
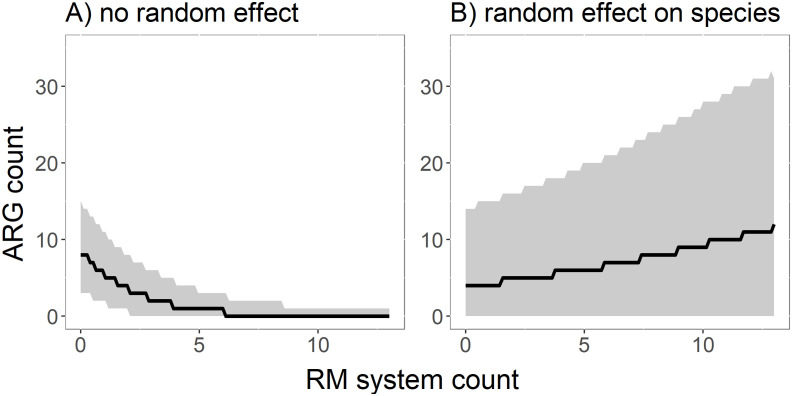
Relationship between the count of RM systems per genome and the count of ARGs per genome as predicted by Bayesian models fit to all taxa data either without (A) or with (B) a random group effect fitted to species identity. Shaded gray area indicates the space within which 95% of predicted draws from the posterior distributions fall. Note, to prevent species with more genomes having a disproportionate influence on the model predictions a dataset comprising random samples of 400 genomes from each species was utilized. The data underlying this Figure are available via Zenodo: https://doi.org/10.5281/zenodo.19387437.

Firstly, to investigate the relationship between RM systems and acquired ARGs at the broadest scale, we modeled this relationship using data from all 14 bacterial species (see Table 1), using RM system and ARG counts per genome as the explanatory and response variables, respectively. We took a random subset of 400 genomes from each species for this analysis to reduce biases in our data. We found that when we did not control for species identity, there was a strongly negative non-linear relationship observed (Fig 2A, Table B in S1 Appendix). However, when species identity was controlled for, this relationship became weakly positive (Fig 2B, Table B in S1 Appendix). This suggests that the initial negative relationship observed is driven by between-species differences, i.e., species with more RM systems tend towards having fewer ARGs.

#### Within species, genomes with more RM systems have more acquired ARGs.

To investigate the relationship between RM systems and acquired ARGs at a finer scale, we conducted within-species analyses for five select species from our larger dataset (*Pseudomonas aeruginosa*, *Acinetobacter baumannii*, *Enterococcus faecium*, *Streptococcus pyogenes*, and *Neisseria gonorrhoeae*), controlling for the effect of phylogeny and genome length. These species were chosen as they are taxonomically diverse, all belong to different families, span three phyla, and span the full range of both ARG and RM system counts per genome seen in the full dataset (see S7–S11 Figs for RM and ARG distribution across phylogenies).

**Fig 3:**
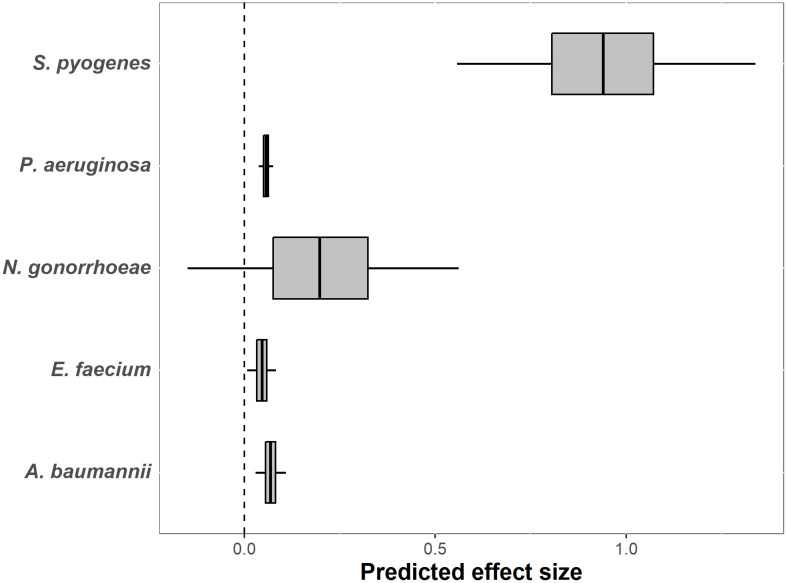
Draws from posterior distributions of the predicted effect of RM system count on ARG count per genome, after controlling for phylogeny and genome length. The predicted effect is the change in count of ARGs per additional RM system, as predicted by a Bayesian Generalized Linear Mixed Model (GLMM). The boxes denote the interquartile ranges, and the whiskers denote the 95% credibility intervals of the predicted effect sizes. The data underlying this Figure are available via Zenodo: https://doi.org/10.5281/zenodo.19387437.

Consistent with the results of the species identity-controlled global analysis, for four out of five species (*Pseudomonas aeruginosa*, *Acinetobacter baumannii*, *Enterococcus faecium*, and *Streptococcus pyogenes*) the predicted relationships between RM systems and ARGs were positive and had 95% credibility intervals that did not span 0 (Fig 3, Table B in S1 Appendix), meaning the models have a high degree of confidence the true relationship is positive. The effect size of RM on ARG count was much larger for *S. pyogenes*, than for the other three species. For *Neisseria gonorrhoeae*, the predicted relationship trended towards being positive, but the 95% credibility interval did span 0 (Fig 3, Table B in S1 Appendix). It is notable that we had much broader posterior distributions for our predictions for *Neisseria gonorrhoeae*, suggesting uncertainty. This could be due to reduced statistical power, owing to it being the species with the fewest genomes and fewest observed ARGs per genome in our dataset.

#### Between- and within-species relationships between RM and MGEs differ due to differences in diversity of RM.

We observed that between species there are negative relationships between the ARG count and the number of RM systems, while within species, we consistently observe positive relationships. Our mathematical modeling predicts that these results may emerge as a consequence of differences in the RM diversity levels across these scales. We therefore hypothesized that as RM system richness between species will necessarily be higher than within species, per HGT opportunity RM systems will pose a more effective barrier to the HGT of ARGs between heterospecifics than between conspecifics.

To test this, we first analyzed the levels of RM system diversity between and within species. We show that all individual species have significantly lower RM system Shannon diversity indices than the entire dataset combined (Table C in S1 Appendix). Next, we calculated for every RM system observed in a species the probability that it would restrict intra- versus interspecific HGT events, based on the sequence similarity between RM systems in each genome. For the five species we conducted intraspecific bioinformatic analyses for, the average probability that a recipient cell will have an RM system that a donor cell does not is 0.69 ± 0.31. Between species, for all 14 species in the analyses, the average probability that a recipient cell will have an RM system that a donor cell does not is 0.96 ± 0.01. Hence, this suggests that on average, per HGT opportunity, RM will more frequently function as a barrier to HGT between species than within species (see Table D in S1 Appendix for all within-species probabilities and all pairwise between-species probabilities), resulting in positive and negative associations between ARGs and RM system counts within and between species, respectively, as predicted by our mathematical model. Note, however, that interspecific HGT opportunities are likely to be rarer than intraspecific HGT opportunities, as heterospecifics are less likely to encounter each other due to ecological niche differences. Consequently, in absolute terms, RM systems may more often act as barriers to intraspecific HGT.

#### ARGs and RM rarely co-localize on the same MGE.

An alternative hypothesis for the positive associations we observed between ARGs and RM within species is that specific RM systems and specific ARGs could be co-localized on the same MGE and acquired together by the same lineage. To investigate the prevalence of this, we identified specific ARG and specific RM system pairs that co-occurred at much higher rates than would be expected by chance. 147 unique pairs of RM systems and ARGs were observed to significantly co-occur (Bonferroni-corrected *P* values < .05, Table F in S1 Appendix). The percentage of genomes carrying a linked pair varied notably across species (*Pseudomonas aeruginosa* = 40.18%, *Acinetobacter baumannii* = 12.32%, *Enterococcus faecium* = 6.27%, *Streptococcus pyogenes* = 1.03%, and *Neisseria gonorrhoeae* 10.13%). To test whether co-occurrence resulted from co-localization, we evaluated the genetic distance between co-occurring pairs, the frequency at which pairs were observed on the same contig, and genomic context of the pair (see Materials and methods, “Identification of co-localization of specific RM systems and specific ARGs”). From this, we identified only one instance where RM-ARG co-occurrence is explained by origin on the same MGE. In this case, the respective Type II RM system and two macrolide resistance genes were co-localized on the previously described *S. pyogenes* conjugative prophage Φ1207.3 [52–54] (S12 Fig). This explains why the positive effect size of the relationship between RM and ARG count per genome is much larger for *S. pyogenes* than for the other species (Fig 3). There is a clinical significance to this co-localization as Type II RM systems can function similarly to toxin-antitoxin (TA) systems [55,56]. As other TA systems have been shown to stabilize ARG carrying MGEs [57], MGEs that carry a Type II RM system as well as ARGs will be under stronger selection for maintenance than MGEs lacking an RM system. Notwithstanding this example of co-localization, we conclude there is no evidence that RM and ARGs co-localizing on the same MGE is driving the positive associations we observed in our within-species analyses. However, it is notable that despite co-localization of specific RM systems and ARGs being very rare, significant co-occurrence is much more common. This is consistent with the idea that there are channels of increased gene exchange between lineages with the same RM systems. If a novel ARG is horizontally acquired by a lineage with a certain RM system, then it will be more readily transferred to other lineages with this RM system, causing the novel ARG and RM system to co-occur significantly more often than would be expected by chance.

#### RM systems are positively associated with ARGs, even when acquired first.

We also hypothesized that, as RM systems are themselves often horizontally transferred [41–44], the order of acquisition of RM systems and ARGs could impact their relationship. For example, if a lineage acquired an RM system more recently than an ARG, the RM system would have no opportunity to pose a barrier to the ARG’s acquisition. To ensure the conclusions we drew from our within-species analyses were not confounded by this, we calculated the ‘trait depth’, a measure of the evolutionary time since genetic material was acquired, for each RM system and each ARG in our dataset (see Materials and methods, “Trait depth filtered modeling”). We then filtered our dataset to include only RM systems that fell within the upper tertile (33rd percentile) for trait depth (the earlier acquired RM systems), and ARGs that fell within the lower tertile for trait depth (more recently acquired ARGs). We then conducted within-species analyses of the relationship between RM systems and ARGs using this trait depth filtered dataset. For *S. pyogenes*, the relationship between count of RM systems and acquired ARGs per genome, which was positive when utilizing all the data, became a null relationship when the model was trained on the trait depth filtered dataset (S13 Fig). This is likely because here our filtering removed the two ARGs and RM system associated with the conjugative prophage Φ1207.3. However, for the other four species, the directionality of the ARG–RM relationship remained unchanged. From this, we conclude that our finding that ARGs are positively associated with RM systems within species is robust.

Interestingly, we found that for four out of five species, RM systems in general had a higher trait depth than ARGs, indicating that they evolved or were acquired more distantly in evolutionary time (S14 Fig). This further reinforces that even when we do not filter our dataset by trait depth, in any given lineage, RM systems are likely to have been present prior to the acquisition of the respective lineage’s ARGs. This disparity in trait depth between RM and ARGs could be explained by selection for antimicrobial resistance increasing dramatically over the last century with the advent of the clinical use of antimicrobials [58], while this is likely not the case for selection for defence systems.

We acknowledge that trait depth is an imperfect proxy for the evolutionary time since mobilizable genes were acquired, as elevated rates of gene gain and loss will erode its correspondence with true acquisition times. However, our inference relies on relative ordering of trait acquisition rather than absolute timescales, and any loss of signal due to gene mobility should act similarly on both ARGs and RM systems. As such, these limitations do not undermine our core comparisons, and trait depth remains an appropriate and informative metric in this context.

#### Conclusions and outlook.

Here, we combine mathematical modeling and bioinformatic analyses to demonstrate that variation in HGT exposure between lineages can lead to positive associations between mobile genes and RM systems, across a range of realistic parameter values. Additionally, we show that increasing population-level RM diversity increases its efficacy as a barrier to gene flux and thus causes the relationship between RM investment and MGE acquisition to become negative. We also demonstrate that the positive associations we observe within species are not confounded by RM systems and ARGs co-localizing on the same MGE, or by lineages acquiring ARGs prior to RM systems.

In this study, we focused on pathogenic bacteria, as these species are frequently exposed to antibiotics in clinical settings and therefore provide a relevant context in which to study the relationship between ARGs and RM systems. However, as we focused solely on pathogens that have very distinct life cycles in comparison to non-pathogenic bacteria, caution should be exercised when extrapolating our findings beyond the species we investigated. Specifically, non-pathogens will likely be under reduced selection for the acquisition of ARGs and MGEs in general when compared with pathogens. Nevertheless, our dataset comprised a broad range of taxonomically and physiologically distinct species that occupy diverse ecological niches and are exposed to different antibiotic treatments in the clinic. Because of this, there is minimal overlap in their ARG content and little evidence of HGT between these species, even in those instances where RM systems are not predicted to form a barrier (Tables D and E in S1 Appendix, and S15 Fig). However, this does not detract from the suitability of this dataset for testing our general hypotheses relating to the relationships between RM systems and ARGs across species (Fig 2), as here we are not assuming HGT is occurring between the study species per se. To reveal how patterns of HGT between co-occurring species in microbial communities are mediated by RM, metagenomic studies will be crucial.

Inevitably, exchange of MGEs will not only depend on bacterial immune systems but also on MGE strategies to overcome bacterial defences [59,60]. Plasmids have been shown to carry many anti-defence systems in the leading region that enters the cell first during conjugation, allowing rapid gene expression and neutralization of host defences [61]. Of these anti-defences, anti-restriction proteins and protective methyltransferases are some of the most abundant [61], highlighting the importance of RM systems in the arms race between bacteria and MGEs. Furthermore, experimental study has shown the anti-restriction proteins and methyltransferases of conjugative plasmids allows them to overcome host RM systems [27] (additionally, the leading region of conjugative plasmids has recently been shown to be depleted in RM target sites [62]). Although our mathematical models did not include anti-restriction, we can speculate that if hosts increase RM system investment in response to rising MGE exposure, MGEs will, in turn, evolve stronger anti-restriction mechanisms. Such an evolutionary arms race could mediate the efficacy of RM systems and contribute further to positive associations between the presence of RM systems and mobile genes.

Here, we illustrate that ecological and evolutionary dynamics can lead to counter-intuitive patterns. We note that previous research has shown that RM systems can also promote prophage acquisition, through allowing phage exposed bacterial populations to reach densities that favor phages entering the lysogenic life cycle [63]. Our findings here add to a growing body of evidence that suggests the interplay between defence systems and MGEs is governed not just by molecular mechanisms, but also by the broader ecological context in which these interactions unfold.

In the present study, we specifically focused on interactions between RM systems and MGEs, as RM systems are highly ubiquitous and are known to pose a barrier to several forms of HGT and types of MGEs [14–27]. However, the rapid expansion of the known microbial defence repertoire over recent years [9,64] raises many questions that future research into defence and MGE evolutionary ecology could aim to answer. For instance, how might other defence mechanisms contribute to eco-evolutionary dynamics? An abortive infection system that arrests growth upon infection [65] could have different implications for evolutionary trajectories compared with systems that degrade MGEs, like RM or CRISPR-Cas. Moreover, how might the specificity of defences impact responses to diverse MGE infections? We hypothesize that the fitness benefit of a broader range defence system, like RM, will depend on exposure to a more diverse range of MGEs, that may span both positive and negative fitness effects. Finally, as single genomes frequently carry several defence systems [66], it is necessary to consider how defences interact [67], from additive effects [68,69], to synergistic [70–74] and even antagonistic interactions [75], to shape the co-evolution of bacteria and MGEs.

There is at present considerable interest in developing a better understanding of how bacterial defence systems interact with and regulate the acquisition of mobile genetic elements. Here, we demonstrate that although RM systems have been shown to be barriers to HGT in more simplistic experiments, in complex microbial communities the relationship between RM systems and gene acquisition is more complex. Our work highlights that if we wish to deepen our understanding of how horizontal transfer of genetic material impacts genomic evolution outside of the laboratory, and how defence systems can impact this, we must synthesize knowledge of the mechanistic nature of these processes with an understanding of the ecological and evolutionary dynamics at play.

## Materials and methods

### Mathematical modeling

#### Model overview.

To determine the ecological conditions under which selection may favor positive associations between defence investment and horizontally acquired genes, we developed a deterministic system of ordinary differential equations (ODEs) describing bacterial population dynamics structured by (i) RM investment and (ii) MGE carriage. We used MATLAB R2023a [76] for all of our simulations.

#### Sub-population structure.

Sub-populations $S_{n,i}$ were defined by RM investment strategy n∈0,r,R (zero, low, high) and MGE number i∈0,…,5, yielding a total of 18 coupled ODEs. High investment into RM results in a high reduction in likelihood of acquiring an MGE, low investment into RM results in a lower reduction in likelihood of acquiring an MGE, and zero investment into RM results in no reduction in likelihood of acquiring an MGE. We assumed an initial condition of one individual of each sub-population. For each simulation, once it had reached a steady state (typically by 4,000 time steps) we measured the relative abundances of every sub-population.

#### Reproduction and death.

Cells reproduced at baseline rate β, subject to logistic density dependence given as $\left(\text{1}-\frac{\sum S(t)}{K}\right)$ where K is carrying capacity. MGE carriage reduced growth multiplicatively as $(\text{1}-c_{\text{mge}}{)}^{i}\;$, meaning additional MGEs reduced the remaining growth potential by a fixed percentage, rather than an absolute value, which is most appropriate when modeling exponential growth [77]. Non-zero RM investment strategies imposed a fixed growth reduction $\left(\text{1}-c_{n}\right)$. Cells died at per-capita rate φ. The change d in sub-population $S_{n,i}$ per unit time t, given as $\frac{dS_{n,i}(t)}{dt}$ due to reproduction and deaths is therefore:


$$\begin{array}{c} \frac{dS_{n,i}(t)}{dt}=\left(\beta {\left(\text{1}-c_{mge}\right)}^{i}\left(\text{1}-c_{n}\right)\right)S_{n,i}(t)\left(\text{1}-\frac{\sum S(t)}{K}\right)-\varphi S_{n,i}(t)\left(\text{1}-\frac{\sum S(t)}{K}\right) \end{array}$$

#### Horizontal gene transfer.

The count of cells in a given sub-population $S_{n,i}$ could increase due to the loss of an MGE from a cell in the $S_{n,(i+\text{1})}$ population and due to the gain of an MGE in a cell in the $S_{n,(i-\text{1})}$ population, and decrease due to the loss or gain of an MGE. Copy number of MGEs was not explicitly modeled, and acquisition of an MGE by a bacterium did not require the loss of an MGE from another bacterium. All five MGEs were identical in their incurred fitness costs.

The acquisition of MGEs depended on number of recipient cells, the relative rate of HGT $\alpha_{h}$, the contact factor ϵ, and the prevalence of MGE-carrying cells in the system. The presence of an RM system reduced the chance of acquiring an MGE. However, RM systems do not limit the transfer of MGEs that originate from sub-populations with the same RM system, as these MGEs would be methylated. Thus, the acquisition of MGEs by sub-populations with an RM was divided into two components. MGEs from sub-populations with a different level of RM investment were acquired at a reduced rate. This rate was given by $\alpha_{n}$ where ${\;\alpha_{n}=\alpha }_{h}/R$ where R was the RM strength (which equaled a reduction in MGE acquisition by a factor of 2^1^ for low investment RM and a factor of 2^7^ for high investment RM).

To approximate the effect of higher RM system diversity without tracking additional sub-populations, we included the additional parameter μ that modified the probability that MGEs acquired from a population with the same RM investment strategy would be correctly methylated. We then conducted two versions of all simulations, with either low or high RM diversity. In low RM diversity simulations μ = 1, meaning that HGT between cells with the same RM investment level was never restricted, which simulated an RM richness of two (one high investment and one low investment RM system). In high RM diversity simulations, to approximate the effect of an RM richness of six, we set μ = 0.333. The modifier μ was never applied to the acquisition of MGEs by zero-RM investment sub-populations.

The decrease in the size of sub-population $S_{n,i}$ due to the gain of MGEs (both MGEs restricted and unrestricted by RM) was therefore given with:


$$\begin{array}{c} -\;{\mu \alpha }_{h}\epsilon \left(\frac{\sum_{n^{\prime }=n}\sum_{i=\text{1}}^{m}S_{n^{\prime },i}(t)}{T}\right)S_{n,i}(t)-\alpha_{n}\epsilon \left(\frac{\sum_{n^{\prime }\neq n}\sum_{i=\text{1}}^{m}S_{n^{\prime },i}(t)}{T}\right)S_{n,i}(t) \end{array}$$

where *T* is the total population size. The increase in size of sub-population $S_{n,i}$ due to the gain of MGEs in sub-population $S_{n,(i-\text{1})}$ was given with:


$$\begin{array}{c} {+\;\mu \alpha }_{h}\epsilon \left(\frac{\sum_{n^{\prime }=n}\sum_{i=\text{1}}^{m}S_{n^{\prime },i}(t)}{T}\right)S_{n,(i-\text{1})}(t){\;+\;\alpha }_{n}\epsilon \left(\frac{\sum_{n^{\prime }\neq n}\sum_{i=\text{1}}^{m}S_{n^{\prime },i}(t)}{T}\right)S_{n,(i-\text{1})}(t) \end{array}$$

Populations with MGEs could lose MGEs at a rate given by the intrinsic MGE clearance rate ʎ and the number of MGEs present, given by i. Therefore, a decrease in sub-population *S*_*n,i*_ due to loss of an MGE was given with:


$$\begin{array}{c} -i\text{ʎ}S_{n,i}(t) \end{array}$$

and an increase in sub-population *S*_*n,i*_ due to loss of an MGE from $S_{n,(i+\text{1})}$ was given with:


$$\begin{array}{c} +i\text{ʎ}S_{n,(i+\text{1})}(t) \end{array}$$

#### Model schematic and full equation.

**Fig 4:**
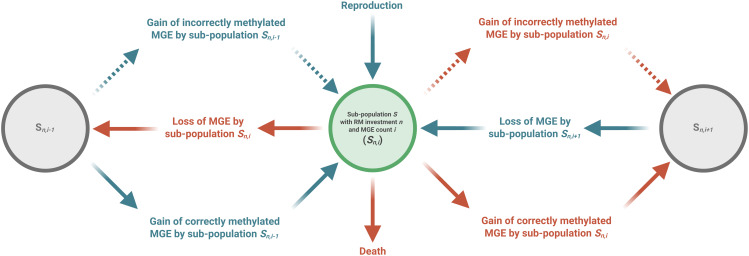
Schematic diagram showing the dynamics that influence the change in size of the focal sub-population ${𝐒}_{n,i}$ (central in green) where 𝐧 denotes RM investment level and i the count of MGEs. Blue arrows moving towards ${𝐒}_{n,i}$ indicate dynamics that increase the sub-population size of ${𝐒}_{n,i}$, whereas red arrows moving away from ${𝐒}_{n,i}$ indicate factors that decrease the sub-population size of ${𝐒}_{n,i}$. Dashed lines indicate where RM systems can limit the transfer of MGEs. Created in https://BioRender.com.


The full equation governing the change in a sub-population’s size (see Fig 4 for schematic diagram) is given with:

$$\begin{array}{c} \frac{dS_{n,i}(t)}{dt}=\left(\beta {\left(\text{1}-c_{mge}\right)}^{i}\left(\text{1}-c_{n}\right)\right)S_{n,i}(t)\left(\text{1}-\frac{\sum S(t)}{K}\right)-\varphi S_{n,i}(t)\left(\text{1}-\frac{\sum S(t)}{K}\right) \end{array}$$

$$\begin{array}{c} -\;{\mu \alpha }_{h}\epsilon \left(\frac{\sum_{n^{\prime }=n}\sum_{i=\text{1}}^{m}S_{n^{\prime },i}(t)}{T}\right)S_{n,i}(t)-\alpha_{n}\epsilon \left(\frac{\sum_{n^{\prime }\neq n}\sum_{i=\text{1}}^{m}S_{n^{\prime },i}(t)}{T}\right)S_{n,i}(t) \end{array}$$

$$\begin{array}{c} {+\;\mu \alpha }_{h}\epsilon \left(\frac{\sum_{n^{\prime }=n}\sum_{i=\text{1}}^{m}S_{n^{\prime },i}(t)}{T}\right)S_{n,(i-\text{1})}(t){\;+\;\alpha }_{n}\epsilon \left(\frac{\sum_{n^{\prime }\neq n}\sum_{i=\text{1}}^{m}S_{n^{\prime },i}(t)}{T}\right)S_{n,(i-\text{1})}(t) \end{array}$$

$$\begin{array}{c} -i\text{ʎ}S_{n,i}(t) \end{array}$$

$$\begin{array}{c} +i\text{ʎ}S_{n,i+\text{1}}(t) \end{array}$$

#### Parameter values.

Simulations were conducted across 11 baseline HGT rates (0–1, step size = 0.1) to assess how HGT exposure influences the relationship between RM investment and per-cell MGE load. To ensure our conclusions were not dependent on specific starting parameters, for all baseline HGT rates, we also ran all simulations across a range of MGE and RM costs (see Table 2 for specific values). The costs of the high investment RM system were informed by previous experimental work demonstrating that RM system costs are generally undetectable to low, but can be up to a 13% reduction in birth rate [78]. As the low investment RM system was defined as 64 times less effective at reducing HGT than the high investment RM system, its cost was scaled to be 64 times less than the respective high investment RM system’s cost. The cost of MGEs varied between a range of values consistent with previous experimental research and modeling of plasmid costs [79–81]. For each combination of fixed MGE and RM investment costs, we pooled the relative abundances of all sub-populations from all simulations across the range of relative rates of HGT simulated.

**Table 2:** Model parameters, symbols used to denote them, and the respective values used.

Parameter	Symbol	Value(s)
Reproductive rate	β	2 births per cell per timestep
Carrying capacity	K	1,000 cells
Contact factor	ϵ	0.9
Cost of one MGE	$c_{mge}$	5%, 10%, 15%, and 20% of birth rate
Cost of high investment RM	$c_{R}$	1%, 3%, 6%, 9%, and 12% of birth rate
Cost of low investment RM	$c_{r}$	0.016%, 0.047%, 0.093%, 0.141%, and 0.188% of birth rate
Death rate	φ	0.5
MGE clearance rate	ʎ	0.015
HGT exposure	$\alpha_{n}$	0 to 1 step size = 0.1

To ensure that our results were not specific to the difference in efficacy between the high and low RM investment strategies, we additionally ran simulations where we varied the factor by which the strong RM system reduced HGT (values spanning 2^3^–2^12^). In these simulations MGE cost was fixed at 15%, the cost of high and low investment RM system carriage was fixed at 3% and 0.04% respectively, and RM diversity was fixed at low.

### Bioinformatics methods

#### Study species.

As a key motivation for this research is to understand how bacterial defence systems may influence the HGT of ARGs, 14 study taxa (Table 1) were selected based on their listing as either of urgent or serious concern in the CDC’s 2019 Antibiotic Resistance Threats Report [82]. For each species, all complete genomes present within the National Centre for Biotechnology Information’s (NCBI) *RefSeq* database [83] were downloaded using the command line tool *ncbi-genome-download* [84] (genomes downloaded August 2022). For our initial global ‘between-species analysis’, all genomes from all 14 species were used (Table 1), but for our within-species phylogenetically controlled analyses we selected five of these species. These species were chosen as they are taxonomically diverse and span the full range of both ARG and RM system counts per genome seen in the full dataset. We also excluded species that lacked heterogeneity in their RM system counts (for example, *Shigella sonnei* and *Streptococcus flexneri* genomes were highly uniform in their RM system content and thus statistical power would be limited).

#### Detection of RM systems.

All bioinformatic analyses were conducted using the University of Exeter’s Cornwall-based High Performance Computing Cluster, known as Athena.

The package PADLOC (Prokaryotic Antiviral Defence LOCator) [85] was used to search all bacterial genomes for defence system genes using Hidden Markov Models (HMMs). The database used was the most up-to-date version of PADLOC-DB available at the time the research was conducted (August 2022), and all RM system gene HMMs used in this study were authored by L. J. Payne [85]. All genes classified in the PADLOC output as belonging to either a Type I, Type II, Type III, or Type IV RM system were then filtered so only hits where the fraction of the target sequence aligning to the HMM, and the fraction of the HMM aligning to the target sequence were both above 0.9, were retained. These values were chosen after adjusting them and conducting multiple sequence alignments in *Geneious Prime 2023.0.1* [86], using the Geneious alignment tool, with random samples of ~20 genes matching the same HMM, and observing that >95% of them would align, suggesting that they were homologs.

#### Validation of RM system functionality.

As annotated RM systems we detect might not always be functional, we conducted cross-validation of our pipeline by comparing predicted RM systems with independent evidence of methyltransferase activity. For each of the 14 bacterial species analyzed we collected all available genomes from the GenBank database for which complete motif modification profiles were also available (https://ftp.ncbi.nlm.nih.gov/pub/supplementary_data/basemodification.csv downloaded August 2025). Not all listed motif summaries and nucleotide sequence files were accessible for download. Note, to increase the size of our validation dataset, we additionally included *E. coli* genomes and epigenetic data, furthermore, no downloadable genomes for *M. tuberculosis* had available motif profiles, so this species was excluded from validation.

In total, we obtained 584 genomes with corresponding motif profiles spanning 14 bacterial species (Table A in S1 Appendix). We then applied our pipeline to detect RM systems in these genomes and compared the count of RM systems detected per genome, with the count of unique DNA modification patterns observed. For most modification patterns, a high proportion of motifs within each genome were methylated (median 97.7%). To conservatively exclude patterns unlikely to represent functional RM systems, we filtered out modification patterns with <95% motif modified within the respective genomes.

We constructed Bland–Altman plots to assess agreement between the number of unique modification patterns and annotated RM systems per genome [87]. We also calculated Pearson’s correlation coefficient to assess the relationship between these measures across genomes, both for total RM systems and stratified by RM system type. Finally, for each species, we tested whether the number of annotated RM systems (excluding Type IV systems, which do not methylate the genome) differed significantly from the number of unique modification patterns.

#### Detection of acquired ARGs.

Methods for the detection of acquired ARGs were informed by and adapted from the scripts used in Pursey *and colleagues* [88,89]. Specifically, the tool *ABRicate* [90] was used to search genomes for acquired antibiotic resistance genes, using the NCBI’s *AMRFinderPlus* database [91].

#### Within-species phylogeny construction.

In order to control for phylogenetic relatedness of genomes, rooted phylogenetic trees were created for each species. First, pairwise distance estimation was conducted using the function *dist* from the package *mash* [92], using a k-mer size of 21. Unrooted phylogenetic trees were created from the resulting pairwise distance matrices using the *nj* (neighbor joining) function of the R package *ape* [93]. These trees were then rooted to an outgroup’s *RefSeq* representative genome, using *ape::root*. Outgroups as close to the ingroup as possible were preferentially selected. Finally, chronograms were created from the rooted trees using *ape::chronos*, the method ‘relaxed’, and a lambda value of 1. Phylogenetic trees were plotted against presence/absence heatmaps of ARGs and RM systems using the tool *ggtree* [94] (see S7–S11 Figs).

#### Calculating diversity indices and probability recipients have RM systems that donors do not.

To test if RM diversity within a species was significantly different to the RM diversity between all species, we performed a Hutcheson *t* test [95] on the respective Shannon diversity indices [96] using the function *Hutcheson_t_test* of the R package *ecolTest* [97].

We also calculated the probability that a HGT event between two genomes of the same species, and two genomes of different species, will be able to be impeded by the recipient genome having an RM system that the donor genome does not.

For all RM systems observed in a species, we calculated the probability that the RM system will not be able to pose a barrier to HGT between conspecifics by adding together the probabilities that both donor and recipient will have the RM system, neither will have the RM system, and only the donor will have the RM system. We then found the mean of this value for all RM systems in the species and subtracted it from 1 to give the average probability that intraspecific HGT will be impeded by RM. We then did this for all pairwise combinations of different species to get the probabilities that RM will impede HGT between species. Note, this was done twice for each pairwise combination, once where species A is the recipient and B is the donor, and once where species A is the donor and B is the recipient.

#### Identification of co-occurrence of specific RM systems and specific ARGs.

To identify instances where a specific RM system and a specific ARG co-occurred more often than would be predicted by chance, the tool Coinfinder [98] was used, in conjunction with the phylogenetic trees. As all possible RM-ARG pairs were tested for co-occurrence, Bonferroni correction was applied to alpha values used to determine significant co-occurrences, adjusting for n hypotheses where n = ARG richness * RM system richness. This makes our estimations of co-occurrence highly conservative. The default Coinfinder setting of excluding rare elements (ARGs or RM systems) was not utilized.

#### Identification of co-localization of specific RM systems and specific ARGs.

To determine if co-occurring pairs were also co-localized, and thus likely to originate from the same HGT event, we calculated the genetic distance between the RM systems and the ARGs, as well as the frequency at which they were observed on the same contig. To identify pairs that were likely candidates for originating on the same MGE, we conservatively filtered the list of co-occurring pairs to include only those where the mean proximity was <100 kb, reducing our 146 pairs to 7 (of these 7 only 3 unique RM systems were observed). A random sample of genomes containing each of these 7 pairs were then selected for further investigation to determine if the contigs in question were potentially extra-chromosomal, or if the regions of DNA containing both RM system and ARG were potentially MGE in origin.

In *Streptococcus pyogenes*, there was one instance of an RM system co-localizing with two macrolide resistance genes and, based on inspection of the adjacent annotations, this region was suspected of being prophage in origin. To confirm this, the prophage search tool VirSorter2 [99] was run on all genomes possessing the co-localizing genes (test), as well as 20 random genomes without these genes (control). All prophage sequences identified from the test genomes were mapped to the largest prophage sequence that spanned the RM system-macrolide resistance gene-containing region using Geneious’ built in ‘map to reference’ tool, using the highest sensitivity settings, and iteratively mapping back to the assembly up to 25 times. To confirm this prophage was not present in the control genomes, the final contig produced from this ‘map to reference’ function was then used as a reference to map all suspected prophages from the control genomes.

Two other RM systems were also observed <100 kb from an ARG that they were significantly found to co-occur with, but in both instances, there was very limited evidence that this was due to origin on same MGE. In *P. aeruginosa*, a Type I RM system that was often located next to integron and integrative conjugative element-associated genes, and in close proximity to several ARGs. However, due to the high variability in the local sequence in this example and known function of integrons to facilitate incorporation of genetic material into the chromosome [100], it is probable that here co-localization is due to this being a ‘hotspot’ of acquired genes, rather than the RM system and ARGs being originally co-localized on the same MGE. In *Acinetobacter baumanii* a Type I system was also observed ~38 kb from a metallo-beta-lactamase, but this co-localization was only observed 5 times, despite the RM system and ARG being observed 237, and 229 times, respectively, and only in the same genome 57 times.

#### Bayesian mixed-effect modeling.

All statistical analyses were conducted in R v. 4.3.1 [101], on the University of Exeter’s RStudio pro server. The *tidyverse* packages [102] were used for data handling. To create figures the R packages *ggplot2* [102], *ggpubr* [103], *cowplot* [104], *RColorBrewer* [105], *gridExtra* [106], and *patchwork* [107] were used. To create tables *flextable* [108] and *ztable* [109] were used.

The R package *brms* was used to conduct all statistical analyses [110], using phylogenetically controlled Bayesian Poisson generalized linear models. All models utilized count of acquired ARGs as the response variable and count of RM systems as the explanatory variable. As we predicted genome length to be positively associated with both ARGs and defence systems, we controlled for it by including it as an additional fixed effect in our models. As *brms* requires a covariance matrix for phylogenetic control, the trees were converted to such using *ape::vcv* [93]. All models were run for 2,000 iterations. The default prior intercept used was a uniform distribution with a lower bound of 0 and an upper bound of the highest ARG count per genome from the respective dataset, this is uninformative yet confined to the bounds of what is possible. For *Streptococcus pyogenes*, *Neisseria gonorrhoeae*, and the ‘all taxa’ models, a slightly more informative prior was needed owing to divergent transitions, and a normal distribution with a mean of the actual ARG mean, a standard deviation (SD) of the real ARG SD, and the aforementioned bounds was used. The slope prior was a gaussian distribution with a mean of 0 and a standard deviation of 1. For the all-taxa analysis, there was no phylogenetic control, but a random group-level effect on species was used. Samples from the modeled posterior distributions were drawn using the *gather_draws* and *add_predicted_draws* functions of the package *tidybayes* [111]. For all models, an r-hat of <1.05 was observed indicating that between- and within-chain variance was equal, and thus chains had converged in their parameter estimates. Bulk and tail effective sample sizes (ESS) were high in all models; >2,000 for all parameters in the phylogenetically controlled within-species analyses, and >500 for all parameters in the ‘all taxa’ analyses.

#### Trait depth filtered modeling.

To calculate the trait depth of both RM systems and ARGs, the function *consentrait_depth* from the R package *castor* was used [112]. Phylogenetic trees used in trait depth estimation were the same used in the phylogenetic controlled within-species modeling (see “Within-species phylogeny construction”). Settings were informed from previous literature [113]. Specifically, for a clade to be considered positive in a trait, the minimum fraction of tips in the clade carrying the trait was 0.9, and all positive clades were weighted equally. Single tips exhibiting a trait were included, and the phylogenetic depth was taken to be half the length of their incoming edge. The mean trait depth was calculated at the level of the species. The trait depth filtered dataset (see S13 Fig), included only the RM systems in the upper tertile for trait depth, and ARGs in the lower tertile for trait depth. These data were then used in phylogenetically controlled Bayesian Poisson GLMs, in the same way as described in “Materials and methods: Bayesian mixed-effect modeling”.

#### Pairwise ARG Jaccard indices calculation and correlation with pairwise probability recipients have RM systems donors do not.

To quantify the pairwise similarities for ARG repertoires between different species, we calculated Jaccard indices. For each pair of ARG sets, the Jaccard index was computed as the number of shared ARGs (i.e., the intersection of the two sets) divided by the total number of unique genes present in either set (i.e., the union of the two sets). This metric ranges from 0 (no shared ARGs) to 1 (identical ARG sets), providing a measure of overlap between species-specific gene repertoires.

We wanted to test if there was a correlation between the pairwise Jaccard indices of ARGs (i.e., the similarity in ARG repertoire between any two species), and the probability that during an HGT event between these two species, a recipient will have an RM system the donor does not (i.e., the probability that RM will pose a barrier to HGT). We hypothesized that pairs of species that had a lower probability that RM would act as a barrier to HGT, would have more similar ARG repertoires (i.e., we predicted a negative correlation between these two metrics). To test this hypothesis, we log 10-transformed both metrics so that they would be normally distributed. We added 10% of the lowest non-zero value to all probabilities a donor will have an RM the recipient does not, in order to prevent infinite values from log transforming zeros. We then used the *ggpubr::stat_cor* function to calculate the Pearson correlation coefficient. To ensure our analysis was robust we also calculated the Pearson correlation coefficient with the zeros removed (just 2.7% of all values), and found our conclusions did not differ.

## Supporting information

S1 Fig The average count of MGEs per individual carried by sub-populations with different levels of RM investment once equilibrium had been reached. Simulations when population-level RM diversity is either A) low or B) high. Panels are divided into columns to denote simulations with different fixed MGE costs (as a percentage reduction in birth rate per MGE) and divided into rows to denote simulations with different fixed RM system investment costs (as a percentage reduction in birth rate, values denote the costs of the high RM investment strategy, low investment RM system costs are scaled linearly). Each panel includes data from all sub-populations and simulations across the range of relative HGT rates. The central horizontal line indicates the mean, the boxes indicate ± 1 standard deviation (SD) from the mean, the whiskers denote ±2 SD from the mean. The data underlying this Figure are available via Zenodo: https://doi.org/10.5281/zenodo.19387437.
(TIFF)

S2 Fig Facets represent simulations with different parameter values for the efficacy of defence in high RM investment sub-populations. Values in facet labels indicate the fold reduction in likelihood of acquiring an unmethylated MGE during an HGT event (higher values indicate greater protection against unmethylated MGEs). Low RM investment always causes a 2-fold reduction in unmethylated MGE acquisition. The cost incurred per MGE carried is fixed at 15%, the cost of carriage of high investment RM systems is fixed at 3%, and the cost of carriage of low investment RM is 0.04%. RM diversity is fixed at low. Each panel includes data from all simulations across the full range of relative HGT rates. All values are for once equilibrium has been reached. A) The average count of MGEs per individual carried by sub-populations with different levels of RM investment. B) The relative size of sub-populations with different levels of RM investment. The data underlying this Figure are available via Zenodo: https://doi.org/10.5281/zenodo.19387437.
(TIFF)

S3 Fig The count of individuals of sub-populations with different levels of RM investment once equilibrium had been reached. Simulations when population-level RM diversity is either A) low or B) high. Panels are divided into columns to denote simulations with different fixed MGE costs (as a percentage reduction in birth rate per MGE) and divided into rows to denote simulations with different fixed RM system investment costs (as a percentage reduction in birth rate, values denote the costs of the high RM investment strategy, low investment RM system costs are scaled linearly). Each panel includes data from all sub-populations and simulations across the range of relative HGT rates. The data underlying this Figure are available via Zenodo: https://doi.org/10.5281/zenodo.19387437.
(TIFF)

S4 Fig The mean count of defence systems per genome for each of the 14 species studied. The data underlying this Figure are available via Zenodo: https://doi.org/10.5281/zenodo.19387437.
(TIFF)

S5 Fig **A)** Bland–Altman plot showing agreement between annotated RM system counts and unique modification patterns per genome. The x-axis shows the per-genome mean of the two measures, and the y-axis shows their difference. Dashed horizontal lines indicate the limits of agreement (mean difference ± 1.96 × SD of the differences), representing the range within which 95% of differences are expected to lie. Points falling outside these limits are labeled by species. Points are slightly offset to improve visualization of overlapping data. **B, C)** Scatterplots showing the relationship between annotated RM system counts and unique methylation patterns per genome. Lines indicate linear fits from a linear model, with shaded ribbons showing the standard error. *R* denotes Pearson’s correlation coefficient and *p* the significance of the relationship. B) All Type I, II, and III RM systems combined. C) Data stratified by RM system type. **D)** Per-species ratios of annotated RM systems (excluding Type IV) to unique methylation patterns (defined as patterns for which >95% of motifs in the respective genome are methylated). Statistical significance of differences between annotated RM system counts and methylation pattern counts for each species was assessed using a Poisson generalized linear model (GLM) (n.s., *p* > 0.05; *** *p* < 0.001). Boxplots show the median (center line), interquartile range (box), and whiskers extending to 1.5× the interquartile range; points beyond this are outliers. The data underlying this Figure are available via Zenodo: https://doi.org/10.5281/zenodo.19387437.
(TIFF)

S6 Fig The mean count of antimicrobial resistance genes (ARGs) per genome for each of the 14 species studied. The data underlying this Figure are available via Zenodo: https://doi.org/10.5281/zenodo.19387437.
(TIFF)

S7 Fig *Pseudomonas aeruginosa* phylogenetic tree with presence of ARGs and RM systems. ARGs are shown in the left heatmap, whereas RM systems are shown in the right heatmap. For each heatmap, genes are arranged from most to least abundant, moving from left to right. Note, in order to aid visualization this tree shows a sub-sample of 200 genomes, not all genomes in the dataset. Where more than 10 different RM systems are observed in the species, the 10 most abundant are shown, and where more than 20 different ARGs are observed in the species, the 20 most abundant are shown. Branch lengths represent Mash genomic distances; the scale bar indicates 0.1 Mash distance units. The data and phylogenetic tree underlying this Figure are available via Zenodo: https://doi.org/10.5281/zenodo.19387437.
(TIFF)

S8 Fig *Acinetobacter baumanii* phylogenetic tree with presence of ARGs and RM systems. ARGs are shown in the left heatmap, whereas RM systems are shown in the right heatmap. For each heatmap, genes are arranged from most to least abundant, moving from left to right. Note, in order to aid visualization this tree shows a sub-sample of 200 genomes, not all genomes in the dataset. Where more than 10 different RM systems are observed in the species, the 10 most abundant are shown, and where more than 20 different ARGs are observed in the species, the 20 most abundant are shown. Branch lengths represent Mash genomic distances; the scale bar indicates 0.1 Mash distance units. The data and phylogenetic tree underlying this Figure are available via Zenodo: https://doi.org/10.5281/zenodo.19387437.
(TIFF)

S9 Fig *Enterococcus faecium* phylogenetic tree with presence of ARGs and RM systems. ARGs are shown in the left heatmap, whereas RM systems are shown in the right heatmap. For each heatmap, genes are arranged from most to least abundant, moving from left to right. Note, in order to aid visualization this tree shows a sub-sample of 200 genomes, not all genomes in the dataset. Where more than 10 different RM systems are observed in the species, the 10 most abundant are shown, and where more than 20 different ARGs are observed in the species, the 20 most abundant are shown. Branch lengths represent Mash genomic distances; the scale bar indicates 0.1 Mash distance units. The data and phylogenetic tree underlying this Figure are available via Zenodo: https://doi.org/10.5281/zenodo.19387437.
(TIFF)

S10 Fig *Streptococcus pyogenes* phylogenetic tree with presence of ARGs and RM systems. ARGs are shown in the left heatmap, whereas RM systems are shown in the right heatmap. For each heatmap, genes are arranged from most to least abundant, moving from left to right. Note, in order to aid visualization this tree shows a sub-sample of 200 genomes, not all genomes in the dataset. Where more than 10 different RM systems are observed in the species, the 10 most abundant are shown, and where more than 20 different ARGs are observed in the species, the 20 most abundant are shown. Branch lengths represent Mash genomic distances; the scale bar indicates 0.1 Mash distance units. The data and phylogenetic tree underlying this Figure are available via Zenodo: https://doi.org/10.5281/zenodo.19387437.
(TIFF)

S11 Fig *Neisseria gonorrhoea* phylogenetic tree with presence of ARGs and RM systems. ARGs are shown in the left heatmap, whereas RM systems are shown in the right heatmap. For each heatmap, genes are arranged from most to least abundant, moving from left to right. Note, in order to aid visualization this tree shows a sub-sample of 200 genomes, not all genomes in the dataset. Where more than 10 different RM systems are observed in the species, the 10 most abundant are shown, and where more than 20 different ARGs are observed in the species, the 20 most abundant are shown. Branch lengths represent Mash genomic distances; the scale bar indicates 0.1 Mash distance units. The data and phylogenetic tree underlying this Figure are available via Zenodo: https://doi.org/10.5281/zenodo.19387437.
(TIFF)

S12 Fig An example of the region of the *Streptococcus pyogenes* prophage Φ1207.3 where two macrolide resistance genes (mef(A) and msr(D)) co-localize with a Type II RM system (MTase_II is the methyltransferase M.SpyI, and REase_II_SU_1 and REase_II_SU_2 are the two subunits of the restriction enzyme). (PDF)

S13 Fig Comparison of models without trait depth filtering (see Fig 3), to those with trait depth filtering “trait depth filtered”. Trait depth filtering refers to a model produced from a dataset where only the RM systems in the upper tertile for trait depth, and ARGs in the lower tertile for trait depth are retained (see Materials and methods: Trait depth filtered modeling). Distributions are draws from posterior distributions of the effect of RM system count on ARG count per genome, after controlling for phylogeny and genome length. The black horizontal line indicates the 95% of draws that fall closest to the mean. Effect size is the effect each additional RM system has on the count of ARGs in a genome. Note X axis scale is not uniform across species. The data underlying this Figure are available via Zenodo: https://doi.org/10.5281/zenodo.19387437.
(TIFF)

S14 Fig Kernel density of trait depths for RM systems and ARGs per species. Higher values indicate that the RM system or ARG was acquired more distantly in the clades evolutionary history. The data underlying this Figure are available via Zenodo: https://doi.org/10.5281/zenodo.19387437.
(TIFF)

S15 Fig The relationship between the pairwise probabilities a recipient has an RM system the donor does not, and the logged pairwise Jaccard indices (a measure of similarity) for ARG repertoire. *R* denotes Pearson’s correlation coefficient and *p* denotes the *p* value for the statistical significance of the correlation. The data underlying this Figure are available via Zenodo: https://doi.org/10.5281/zenodo.19387437.
(TIFF)

S1 Appendix Eco-evolutionary feedbacks drive the co-occurrence of restriction-modification systems and antimicrobial resistance genes in bacteria: Supplementary Information. **Table A in S1 Appendix.** Summary data for the genomes used to verify our RM system detection pipeline. ‘Genomes’ indicate the count of genomes used, ‘Type X RM systems’ indicates the total count of annotated RM systems of the respective type, ‘Total Type I, II, & III RM’ indicates the sum of Type I-III RM systems (those that methylate the host genome), ‘Modification patterns’ indicates the count of unique modification patterns where >95% of motifs in the respective genome are modified. ‘*P* value’ indicates the *P* value derived from a Poisson generalized linear model (GLM) testing whether the per genome counts of ‘Total Type I, II, and III RM’ and ‘Modification patterns’ are statistically different for the respective species (with false discovery rate correction for multiple testing applied). ‘Estimate’ indicates the regression coefficient from the Poisson GLM on the log scale, representing the direction and magnitude of the difference between counts. ‘Lower CI’ and ‘Upper CI’ indicate the bounds of the 95% confidence interval for the estimate on the log scale. The data underlying this Table are available via Zenodo: https://doi.org/10.5281/zenodo.19387437. **Table B in S1 Appendix.** Statistics output from the Bayesian models utilized in the phylogenetically controlled within-species analyses. ‘Estimate’ denotes the median value drawn from the posterior distribution of the effect of the explanatory variable (Effect) on the response variable (ARG count per genome). ‘lwr 95% CI’ denotes the lower 95% credibility interval of the estimate, ‘upr 95% CI’ denotes the upper 95% credibility interval of the estimate, R-hat is a quantification of the Markov chain Monte Carlo chain convergence, Bulk ESS is the effective sample size for rank normalized values using split chains, Tail ESS is the minimum of the effective sample sizes for 5% and 95% quantiles. The data underlying this Table are available via Zenodo: https://doi.org/10.5281/zenodo.19387437. **Table C in S1 Appendix.** Shannon diversity indices for the RM system content of each species, and the P value for the Hutcheson t *t*est conducted to test if the respective species’ diversity index is significantly lower than the Shannon diversity index of all species combined. The data underlying this Table are available via Zenodo: https://doi.org/10.5281/zenodo.19387437. **Table D in S1 Appendix.** The pairwise probabilities that an HGT event between a donor of one species (rows) and a recipient of another species (columns) will be restricted by RM (due to the recipient having an RM system that the donor does not). Values indicate the mean probabilities for all RM systems present in the recipient species. ‘Conspecific donor’ indicates that the donor is the same species as the recipient. The data underlying this Table are available via Zenodo: https://doi.org/10.5281/zenodo.19387437. **Table E in S1 Appendix.** Jaccard indices of the ARG repertoire for each pair of species in the dataset. Values fall between 0 and 1, where 0 indicates the respective species share no ARGs, and 1 indicates both species possess an identical repertoire of ARGs. The data underlying this Table are available via Zenodo: https://doi.org/10.5281/zenodo.19387437. **Table F in S1 Appendix**. Pairs of RM systems and ARGs that co-occur significantly more than expected by chance (using Bonferroni corrected alpha values), where the effect size of the co-occurrence is large (observed co-occurrence ≥ 10* expected co-occurrence) and excluding rare pairs that co-occur in fewer than 0.5% of the respective species’ genomes. The data underlying this Table are available via Zenodo: https://doi.org/10.5281/zenodo.19387437.
(DOCX)

## Abbreviations

ARGs: antimicrobial resistance genes
HGT: horizontal gene transfer
LOA: limits of agreement
MGEs: mobile genetic elements
NCBI: National Centre for Biotechnology Information
ODEs: ordinary differential equations
RM: restriction-modification
SD: standard deviation
