# Supplementary figures and images for: Eco-evolutionary feedbacks drive the co-occurrence of restriction-modification systems and antimicrobial resistance genes in bacteria
Source: PLoS Biol. 2026 Jun 15;24(6):e3003842. doi: 10.1371/journal.pbio.3003842 (PMC13293514; doi:10.1371/journal.pbio.3003842)

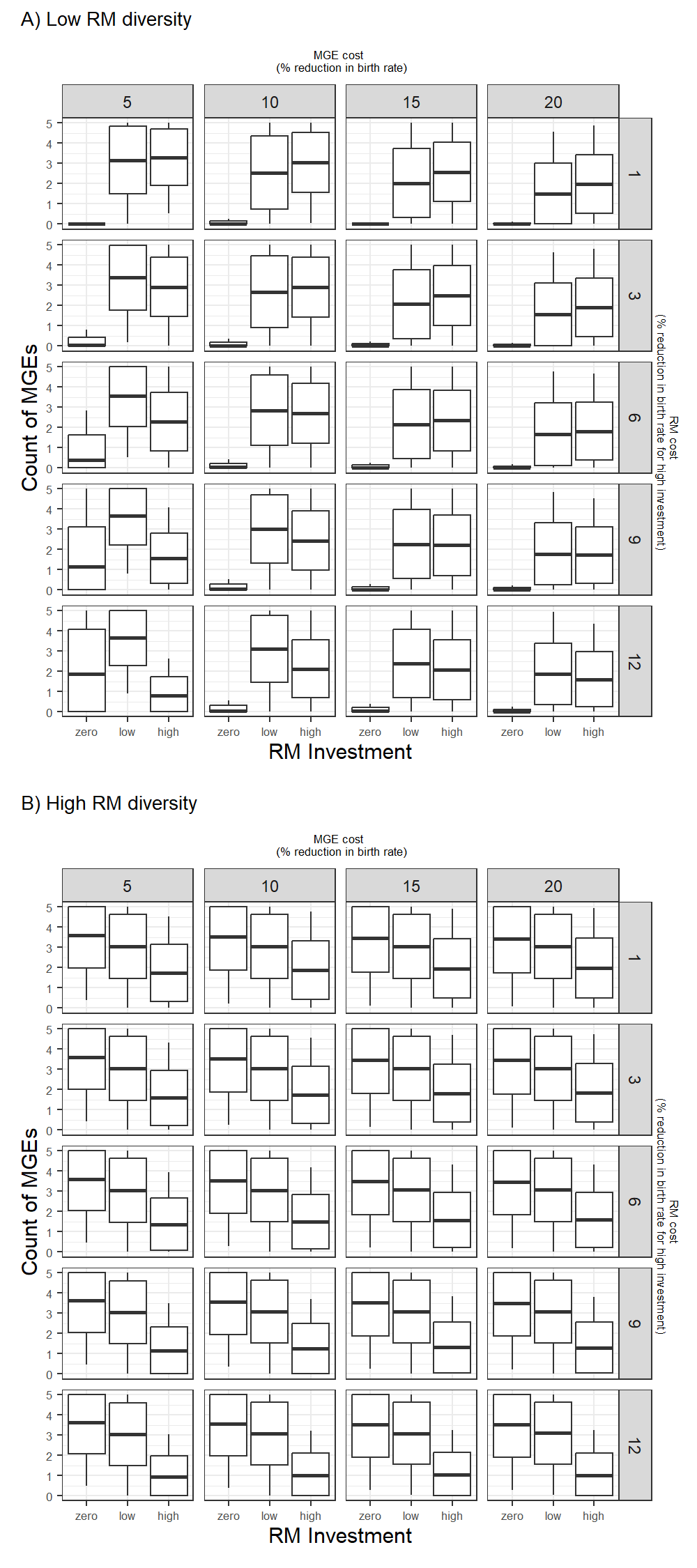

Supplement: S1 Fig — Simulations when population-level RM diversity is either A) low or B) high. Panels are divided into columns to denote simulations with different fixed MGE costs (as a percentage reduction in birth rate per MGE) and divided into rows to denote simulations with different fixed RM system investment costs (as a percentage reduction in birth rate, values denote the costs of the high RM investment strategy, low investment RM system costs are scaled linearly). Each panel includes data from all sub-populations and simulations across the range of relative HGT rates. The central horizontal line indicates the mean, the boxes indicate ± 1 standard deviation (SD) from the mean, the whiskers denote ±2 SD from the mean. The data underlying this Figure are available via Zenodo: https://doi.org/10.5281/zenodo.19387437. (TIFF) [file pbio.3003842.s001.tiff]

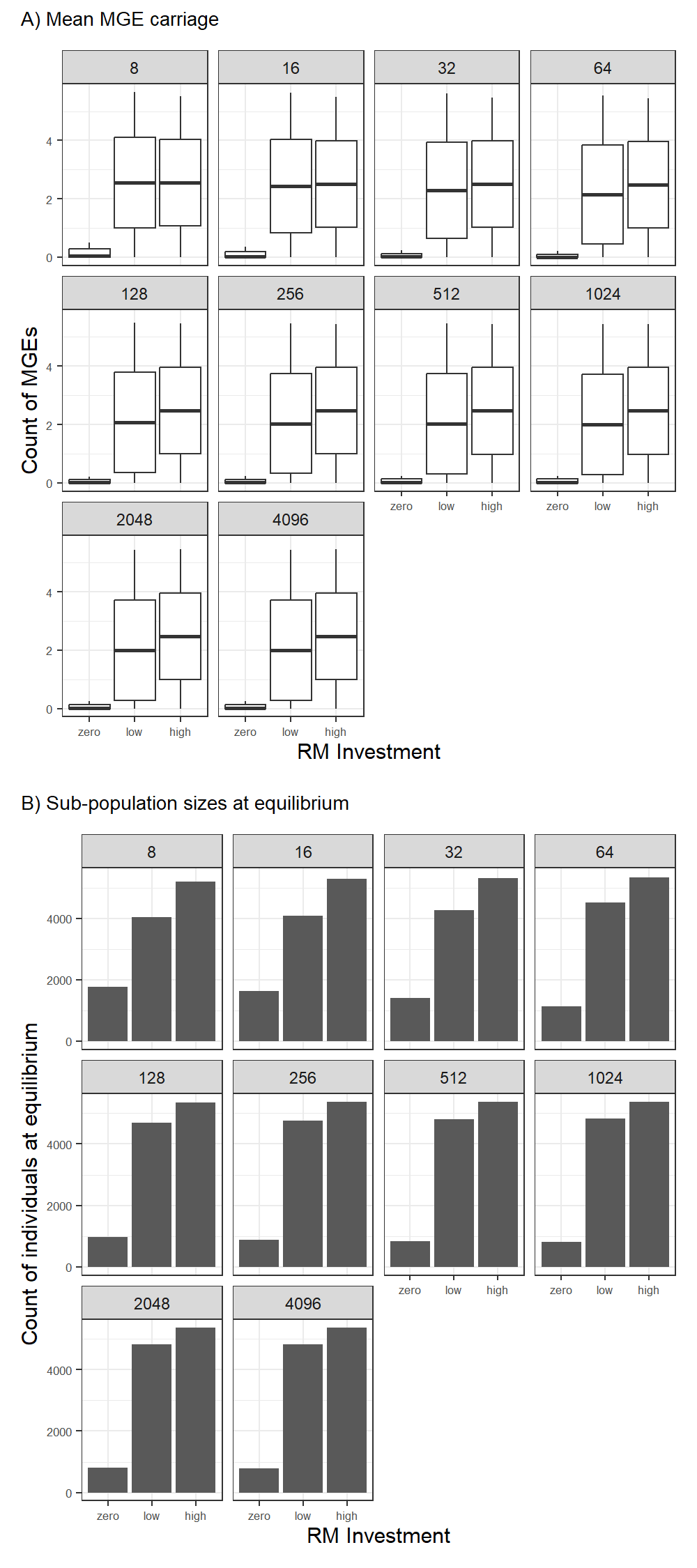

Supplement: S2 Fig — Values in facet labels indicate the fold reduction in likelihood of acquiring an unmethylated MGE during an HGT event (higher values indicate greater protection against unmethylated MGEs). Low RM investment always causes a 2-fold reduction in unmethylated MGE acquisition. The cost incurred per MGE carried is fixed at 15%, the cost of carriage of high investment RM systems is fixed at 3%, and the cost of carriage of low investment RM is 0.04%. RM diversity is fixed at low. Each panel includes data from all simulations across the full range of relative HGT rates. All values are for once equilibrium has been reached. A) The average count of MGEs per individual carried by sub-populations with different levels of RM investment. B) The relative size of sub-populations with different levels of RM investment. The data underlying this Figure are available via Zenodo: https://doi.org/10.5281/zenodo.19387437. (TIFF) [file pbio.3003842.s002.tiff]

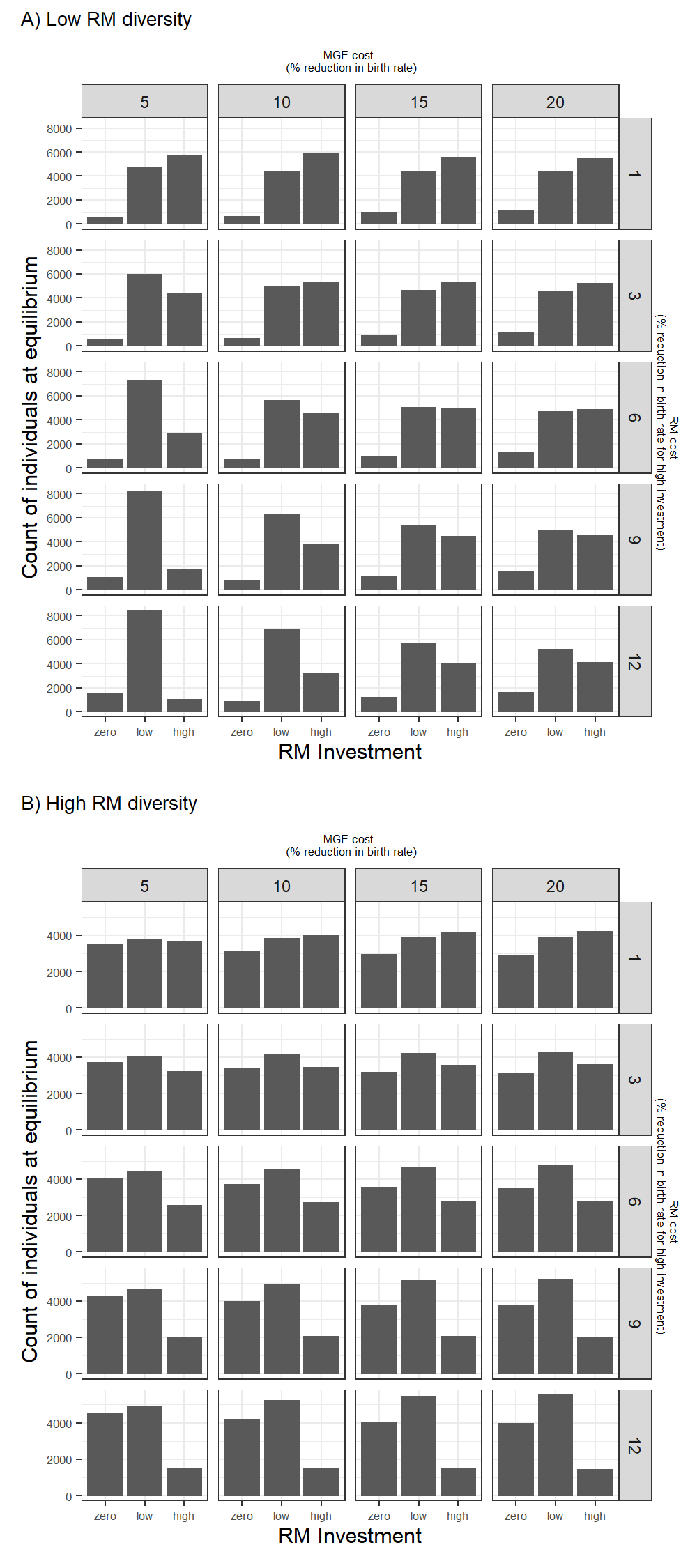

Supplement: S3 Fig — Simulations when population-level RM diversity is either A) low or B) high. Panels are divided into columns to denote simulations with different fixed MGE costs (as a percentage reduction in birth rate per MGE) and divided into rows to denote simulations with different fixed RM system investment costs (as a percentage reduction in birth rate, values denote the costs of the high RM investment strategy, low investment RM system costs are scaled linearly). Each panel includes data from all sub-populations and simulations across the range of relative HGT rates. The data underlying this Figure are available via Zenodo: https://doi.org/10.5281/zenodo.19387437. (TIFF) [file pbio.3003842.s003.tiff]

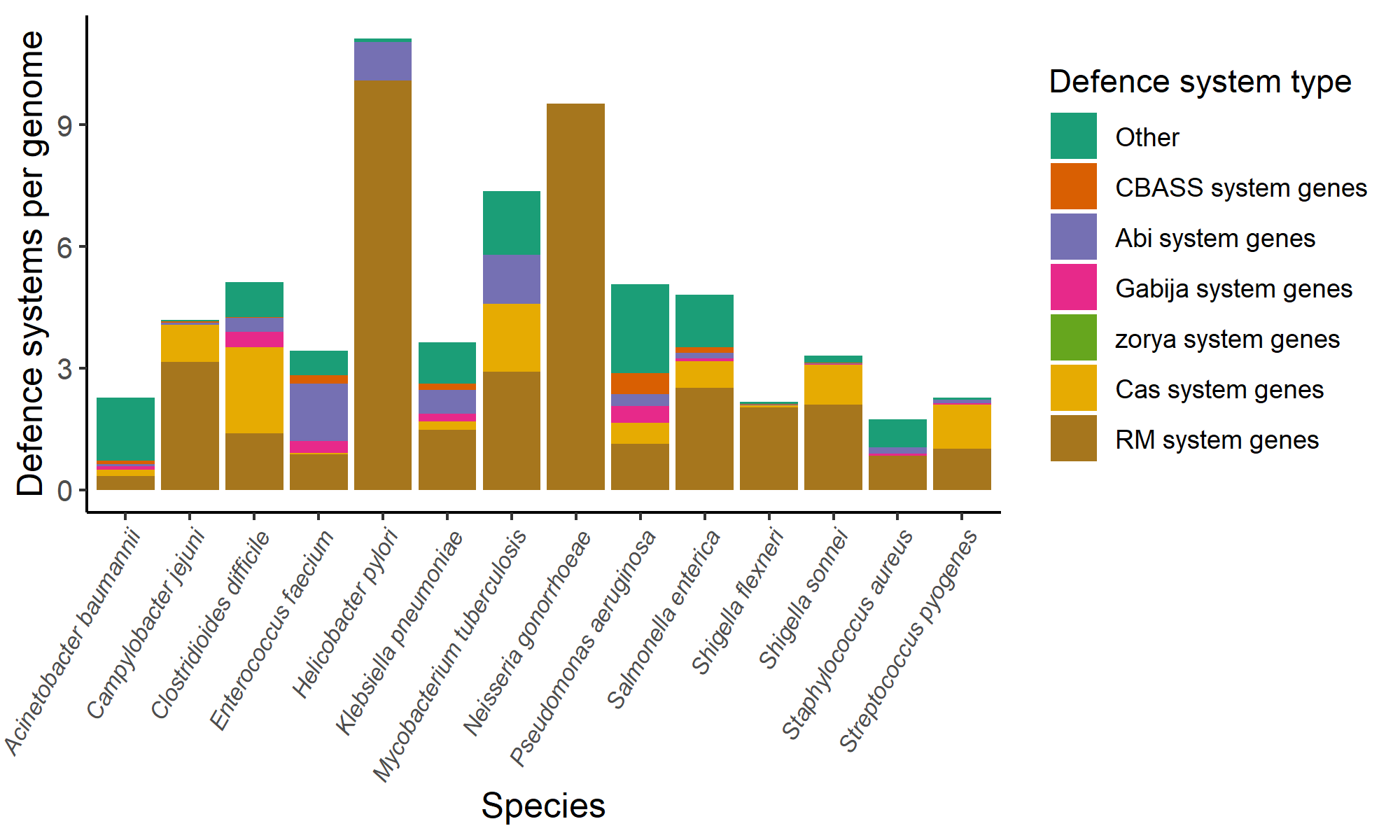

Supplement: S4 Fig — The data underlying this Figure are available via Zenodo: https://doi.org/10.5281/zenodo.19387437. (TIFF) [file pbio.3003842.s004.tiff]

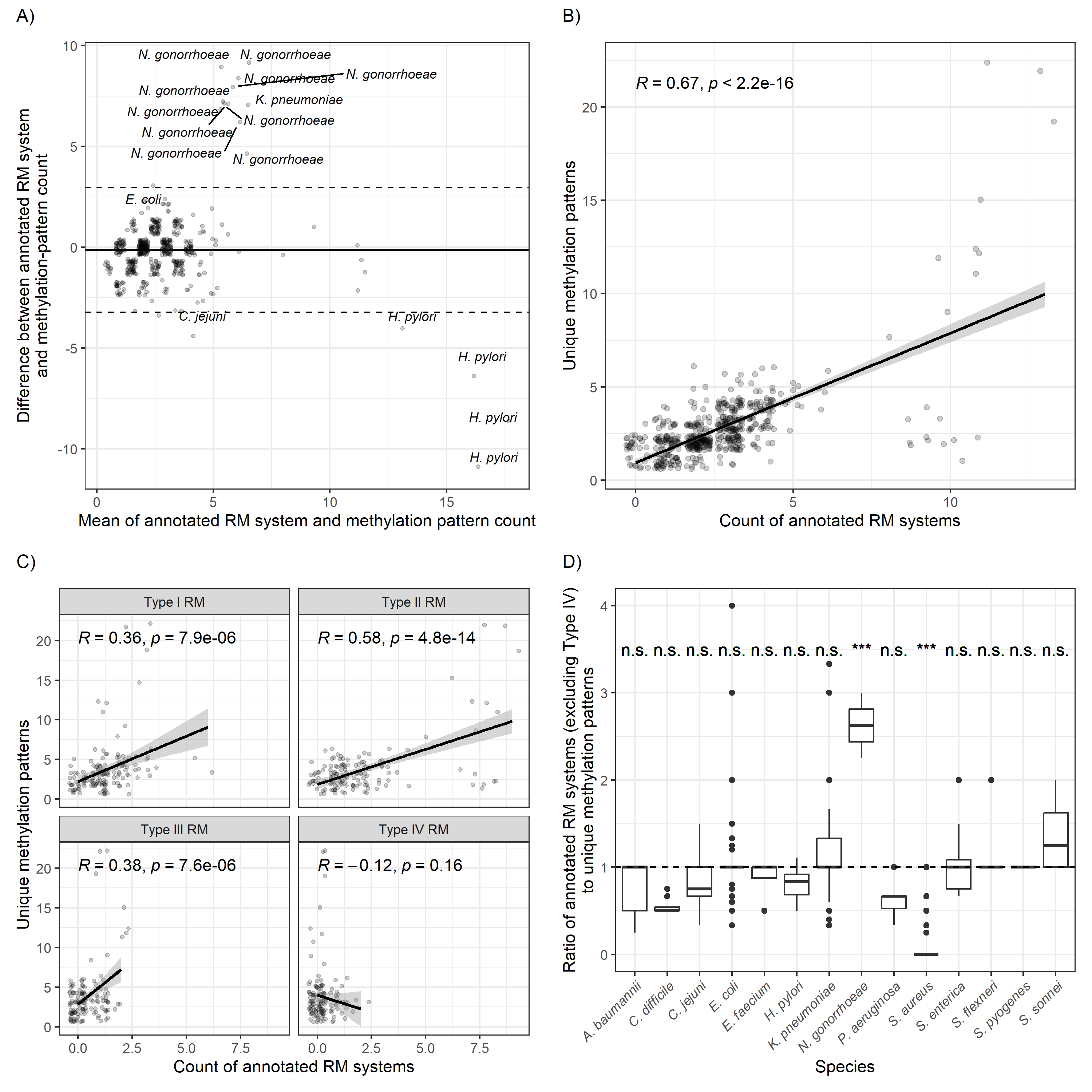

Supplement: S5 Fig — A) Bland–Altman plot showing agreement between annotated RM system counts and unique modification patterns per genome. The x-axis shows the per-genome mean of the two measures, and the y-axis shows their difference. Dashed horizontal lines indicate the limits of agreement (mean difference ± 1.96 × SD of the differences), representing the range within which 95% of differences are expected to lie. Points falling outside these limits are labeled by species. Points are slightly offset to improve visualization of overlapping data. B, C) Scatterplots showing the relationship between annotated RM system counts and unique methylation patterns per genome. Lines indicate linear fits from a linear model, with shaded ribbons showing the standard error. R denotes Pearson’s correlation coefficient and p the significance of the relationship. B) All Type I, II, and III RM systems combined. C) Data stratified by RM system type. D) Per-species ratios of annotated RM systems (excluding Type IV) to unique methylation patterns (defined as patterns for which >95% of motifs in the respective genome are methylated). Statistical significance of differences between annotated RM system counts and methylation pattern counts for each species was assessed using a Poisson generalized linear model (GLM) (n.s., p > 0.05; *** p < 0.001). Boxplots show the median (center line), interquartile range (box), and whiskers extending to 1.5× the interquartile range; points beyond this are outliers. The data underlying this Figure are available via Zenodo: https://doi.org/10.5281/zenodo.19387437. (TIFF) [file pbio.3003842.s005.tiff]

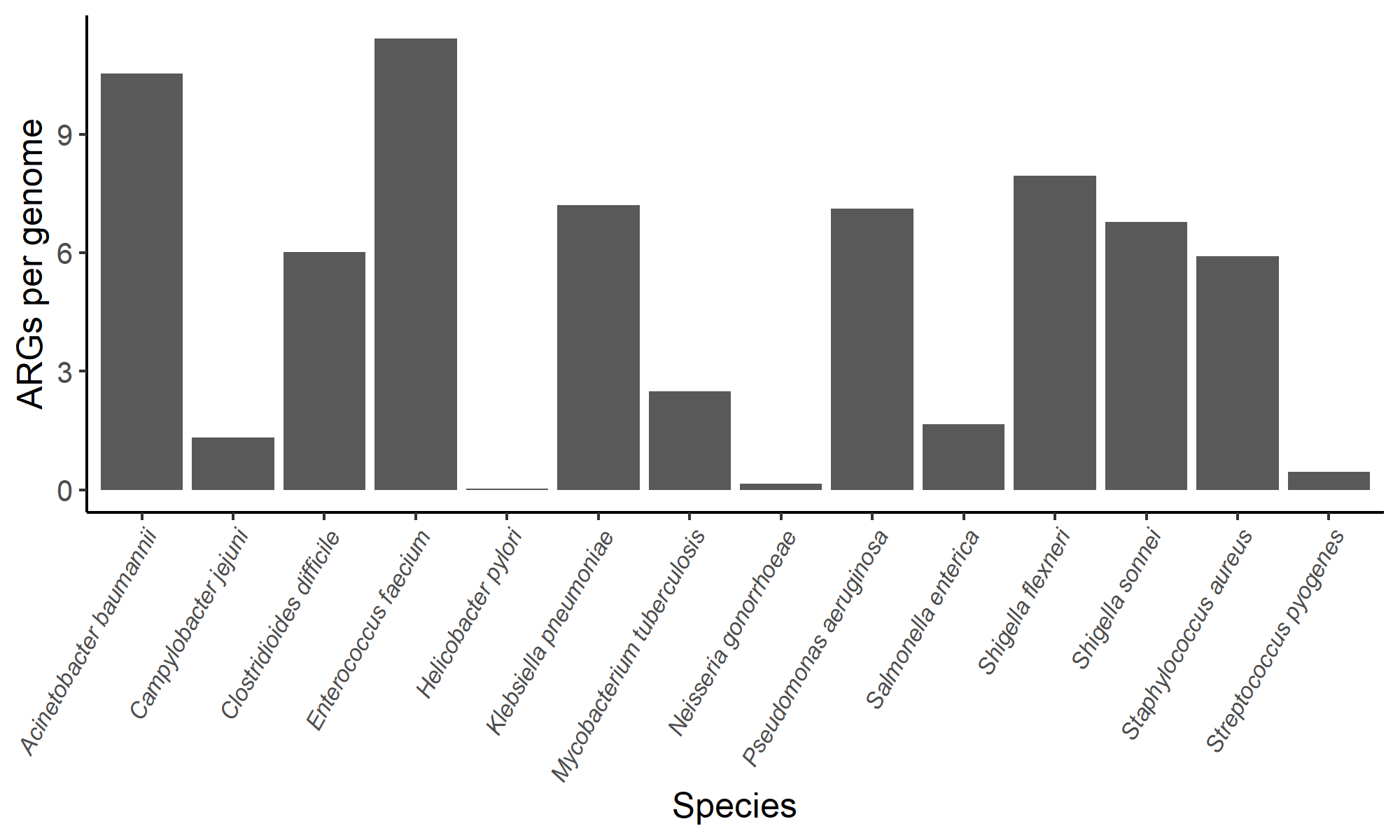

Supplement: S6 Fig — The data underlying this Figure are available via Zenodo: https://doi.org/10.5281/zenodo.19387437. (TIFF) [file pbio.3003842.s006.tiff]

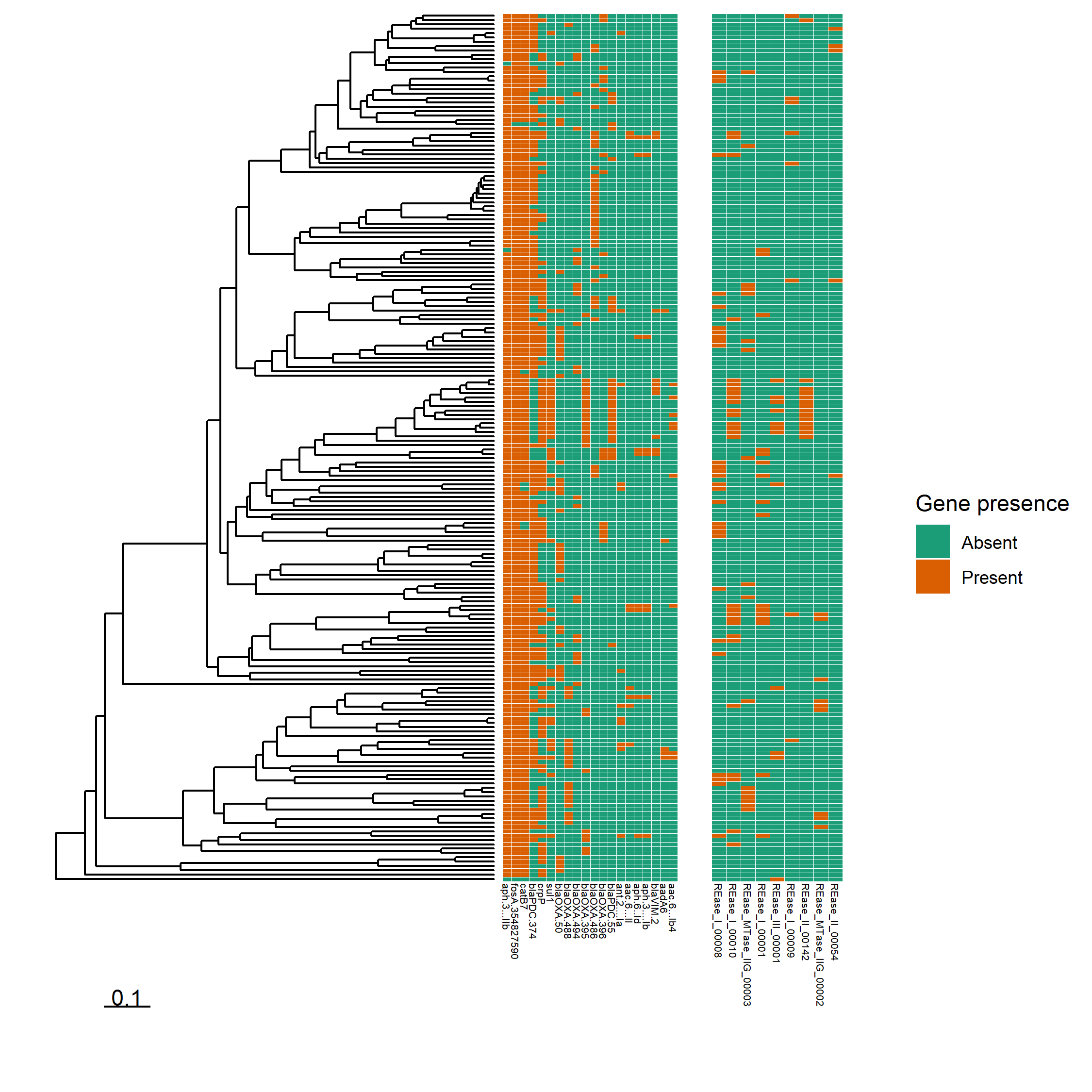

Supplement: S7 Fig — ARGs are shown in the left heatmap, whereas RM systems are shown in the right heatmap. For each heatmap, genes are arranged from most to least abundant, moving from left to right. Note, in order to aid visualization this tree shows a sub-sample of 200 genomes, not all genomes in the dataset. Where more than 10 different RM systems are observed in the species, the 10 most abundant are shown, and where more than 20 different ARGs are observed in the species, the 20 most abundant are shown. Branch lengths represent Mash genomic distances; the scale bar indicates 0.1 Mash distance units. The data and phylogenetic tree underlying this Figure are available via Zenodo: https://doi.org/10.5281/zenodo.19387437. (TIFF) [file pbio.3003842.s007.tiff]

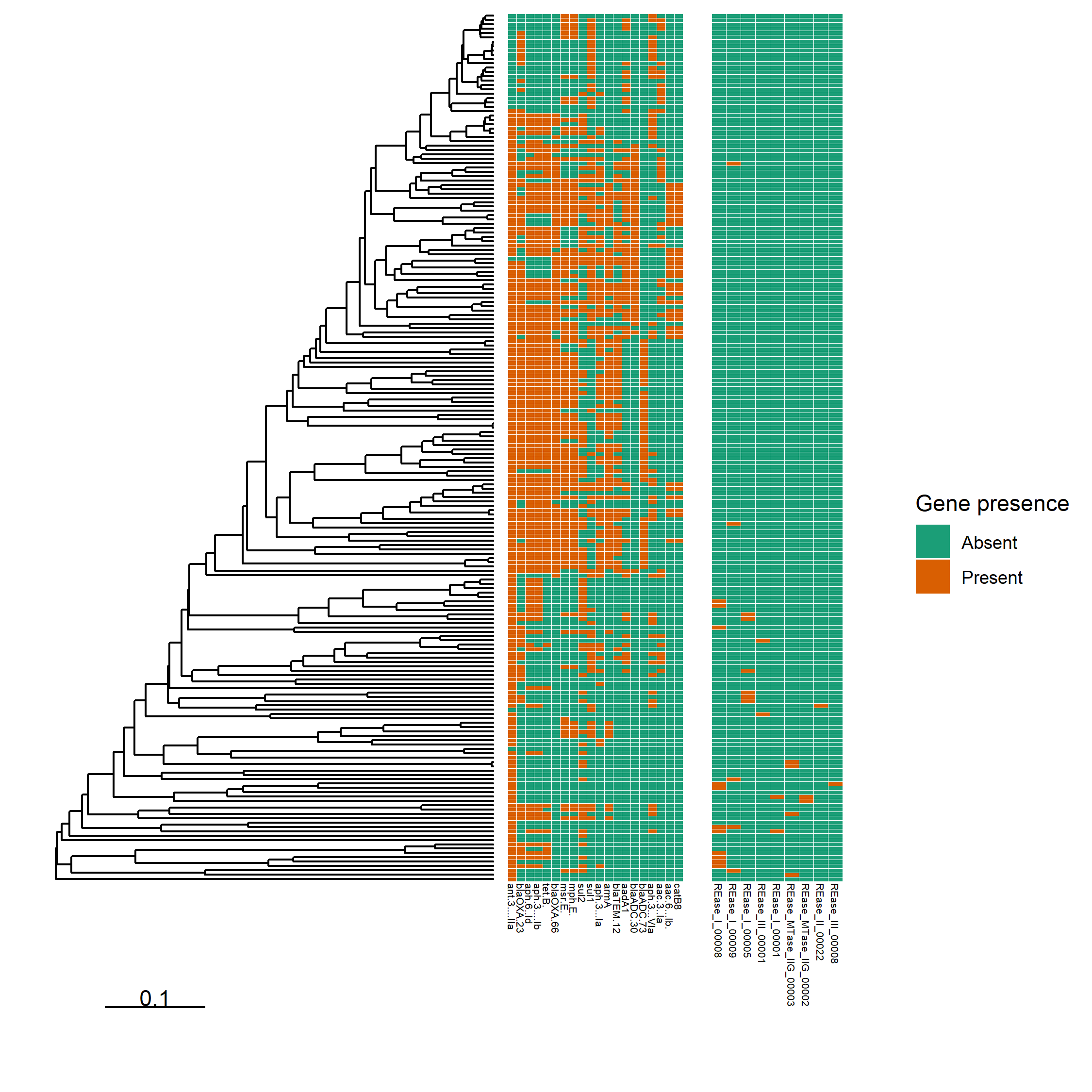

Supplement: S8 Fig — ARGs are shown in the left heatmap, whereas RM systems are shown in the right heatmap. For each heatmap, genes are arranged from most to least abundant, moving from left to right. Note, in order to aid visualization this tree shows a sub-sample of 200 genomes, not all genomes in the dataset. Where more than 10 different RM systems are observed in the species, the 10 most abundant are shown, and where more than 20 different ARGs are observed in the species, the 20 most abundant are shown. Branch lengths represent Mash genomic distances; the scale bar indicates 0.1 Mash distance units. The data and phylogenetic tree underlying this Figure are available via Zenodo: https://doi.org/10.5281/zenodo.19387437. (TIFF) [file pbio.3003842.s008.tiff]

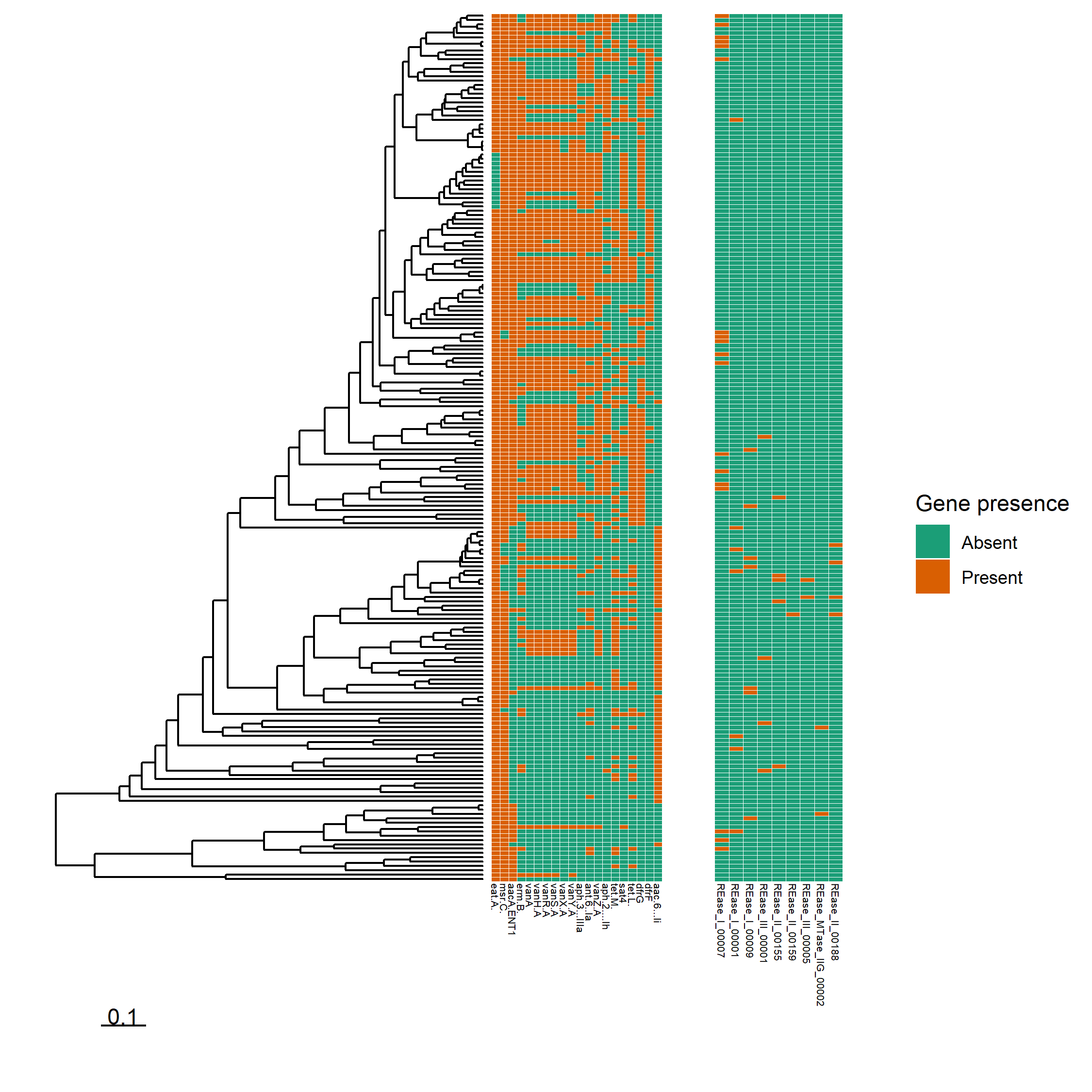

Supplement: S9 Fig — ARGs are shown in the left heatmap, whereas RM systems are shown in the right heatmap. For each heatmap, genes are arranged from most to least abundant, moving from left to right. Note, in order to aid visualization this tree shows a sub-sample of 200 genomes, not all genomes in the dataset. Where more than 10 different RM systems are observed in the species, the 10 most abundant are shown, and where more than 20 different ARGs are observed in the species, the 20 most abundant are shown. Branch lengths represent Mash genomic distances; the scale bar indicates 0.1 Mash distance units. The data and phylogenetic tree underlying this Figure are available via Zenodo: https://doi.org/10.5281/zenodo.19387437. (TIFF) [file pbio.3003842.s009.tiff]

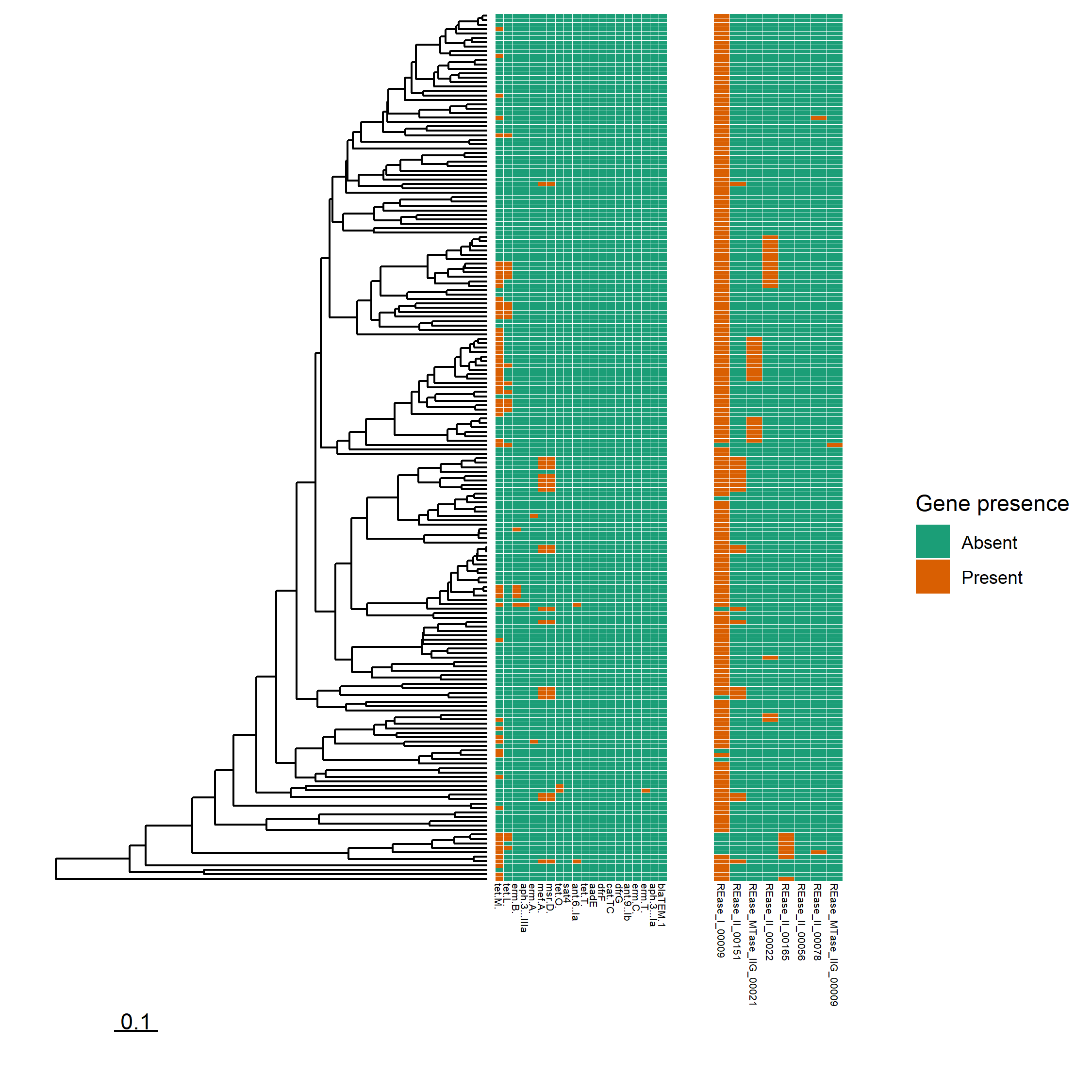

Supplement: S10 Fig — ARGs are shown in the left heatmap, whereas RM systems are shown in the right heatmap. For each heatmap, genes are arranged from most to least abundant, moving from left to right. Note, in order to aid visualization this tree shows a sub-sample of 200 genomes, not all genomes in the dataset. Where more than 10 different RM systems are observed in the species, the 10 most abundant are shown, and where more than 20 different ARGs are observed in the species, the 20 most abundant are shown. Branch lengths represent Mash genomic distances; the scale bar indicates 0.1 Mash distance units. The data and phylogenetic tree underlying this Figure are available via Zenodo: https://doi.org/10.5281/zenodo.19387437. (TIFF) [file pbio.3003842.s010.tiff]

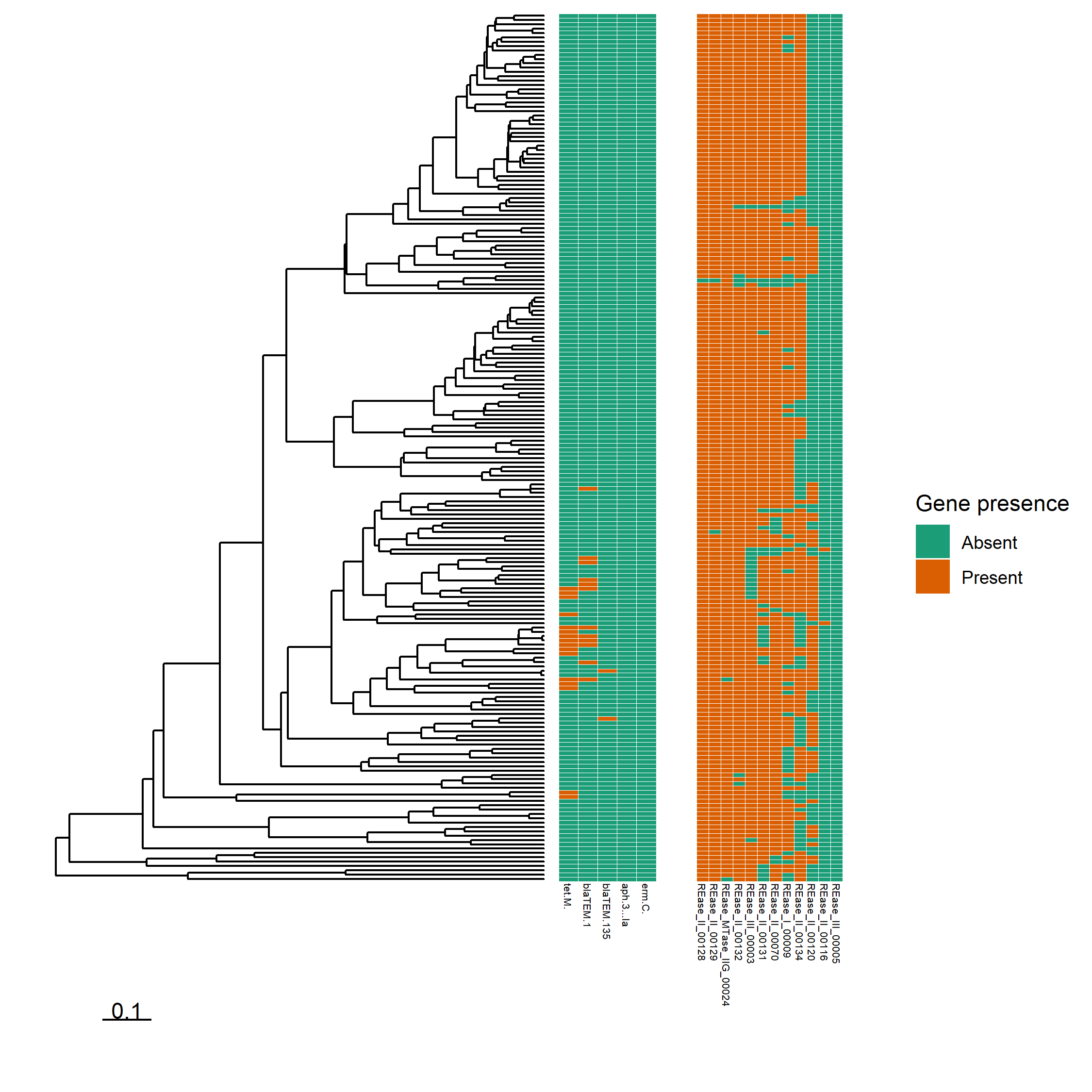

Supplement: S11 Fig — ARGs are shown in the left heatmap, whereas RM systems are shown in the right heatmap. For each heatmap, genes are arranged from most to least abundant, moving from left to right. Note, in order to aid visualization this tree shows a sub-sample of 200 genomes, not all genomes in the dataset. Where more than 10 different RM systems are observed in the species, the 10 most abundant are shown, and where more than 20 different ARGs are observed in the species, the 20 most abundant are shown. Branch lengths represent Mash genomic distances; the scale bar indicates 0.1 Mash distance units. The data and phylogenetic tree underlying this Figure are available via Zenodo: https://doi.org/10.5281/zenodo.19387437. (TIFF) [file pbio.3003842.s011.tiff]

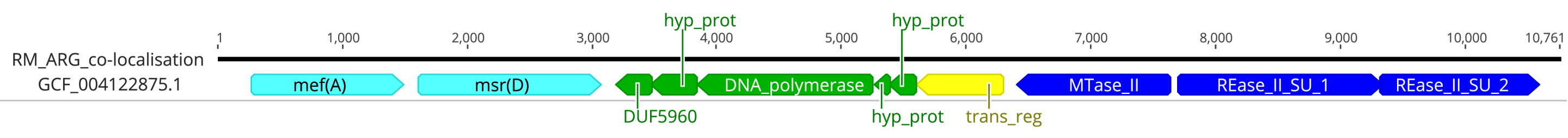

Supplement: S12 Fig — (PDF) [file pbio.3003842.s012.pdf]

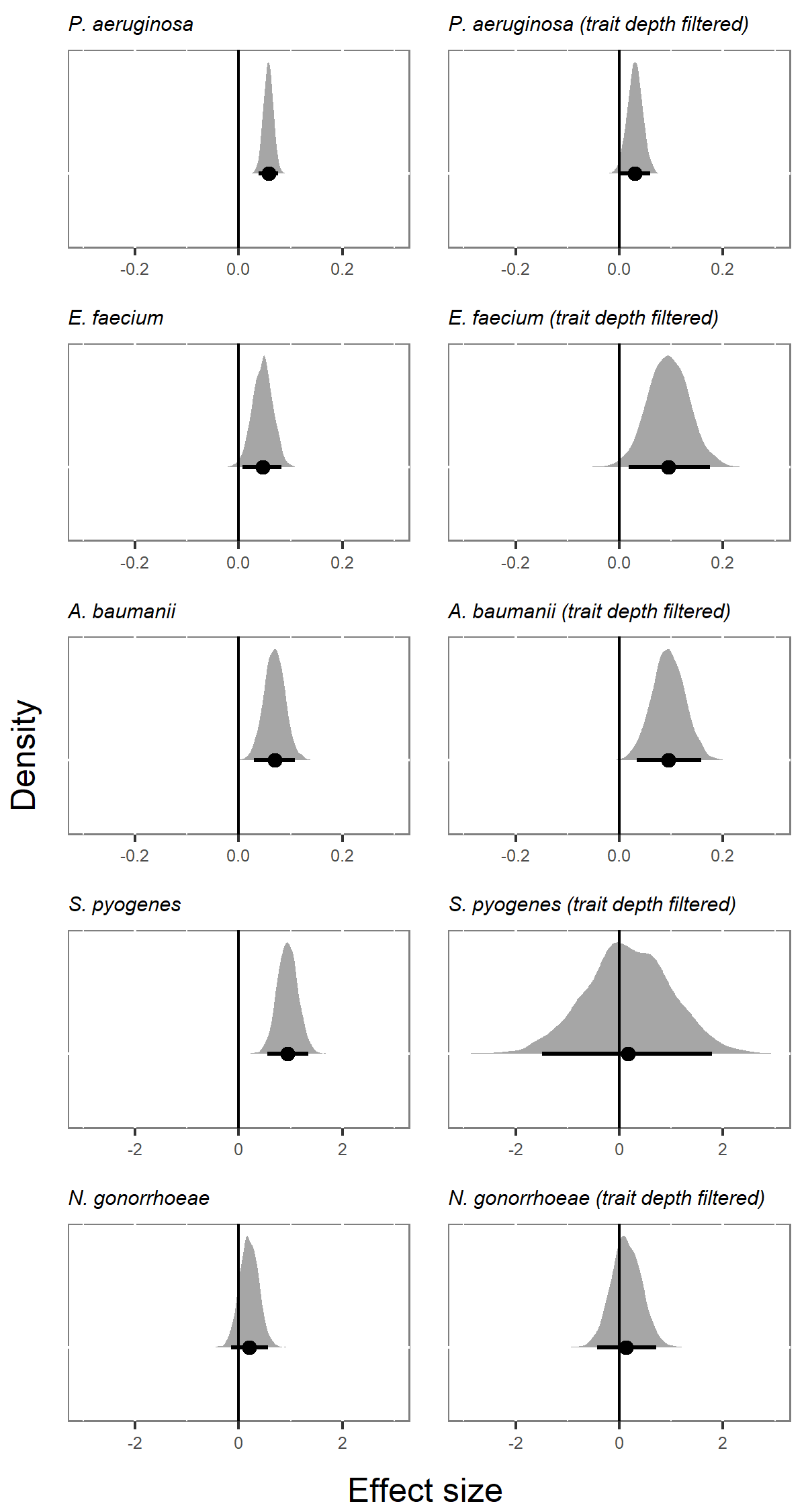

Supplement: S13 Fig — Trait depth filtering refers to a model produced from a dataset where only the RM systems in the upper tertile for trait depth, and ARGs in the lower tertile for trait depth are retained (see Materials and methods: Trait depth filtered modeling). Distributions are draws from posterior distributions of the effect of RM system count on ARG count per genome, after controlling for phylogeny and genome length. The black horizontal line indicates the 95% of draws that fall closest to the mean. Effect size is the effect each additional RM system has on the count of ARGs in a genome. Note X axis scale is not uniform across species. The data underlying this Figure are available via Zenodo: https://doi.org/10.5281/zenodo.19387437. (TIFF) [file pbio.3003842.s013.tiff]

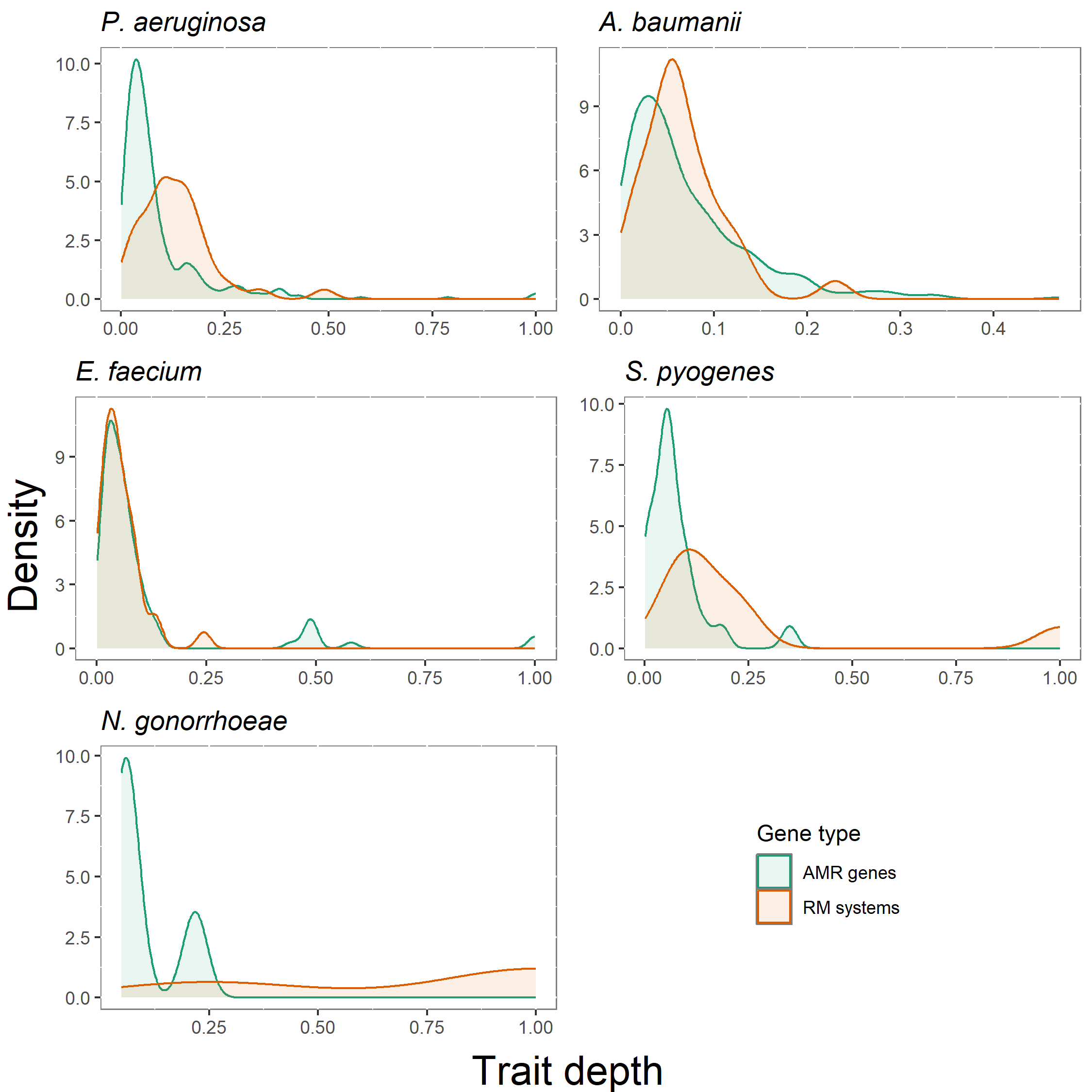

Supplement: S14 Fig — Higher values indicate that the RM system or ARG was acquired more distantly in the clades evolutionary history. The data underlying this Figure are available via Zenodo: https://doi.org/10.5281/zenodo.19387437. (TIFF) [file pbio.3003842.s014.tiff]

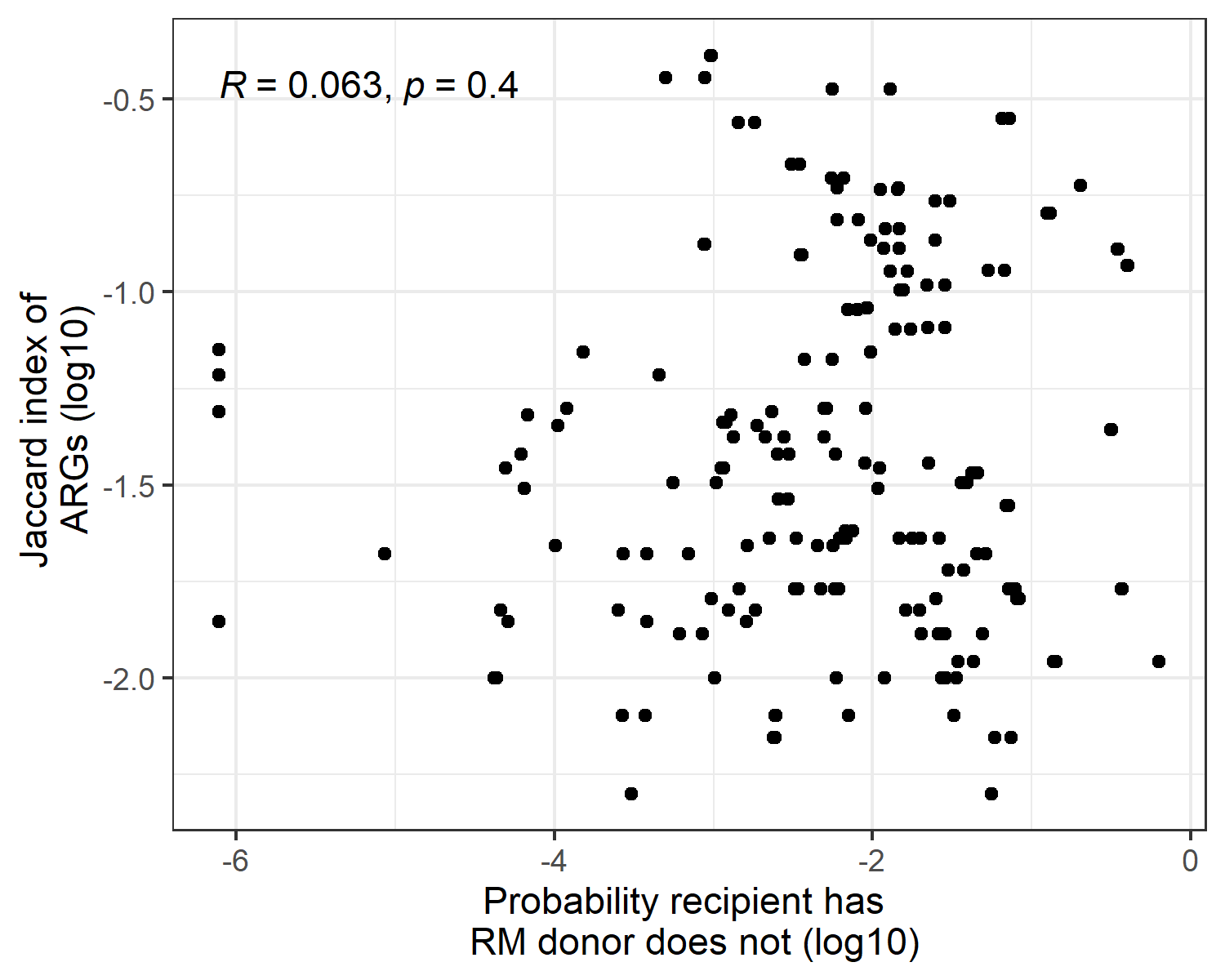

Supplement: S15 Fig — R denotes Pearson’s correlation coefficient and p denotes the p value for the statistical significance of the correlation. The data underlying this Figure are available via Zenodo: https://doi.org/10.5281/zenodo.19387437. (TIFF) [file pbio.3003842.s015.tiff]
