# Supplementary material for: Eco-evolutionary feedbacks drive the co-occurrence of restriction-modification systems and antimicrobial resistance genes in bacteria
Source: PLoS Biol. 2026 Jun 15;24(6):e3003842. doi: 10.1371/journal.pbio.3003842 (PMC13293514; doi:10.1371/journal.pbio.3003842)
Supplement: S1 Appendix — Table A in S1 Appendix. Summary data for the genomes used to verify our RM system detection pipeline. ‘Genomes’ indicate the count of genomes used, ‘Type X RM systems’ indicates the total count of annotated RM systems of the respective type, ‘Total Type I, II, & III RM’ indicates the sum of Type I-III RM systems (those that methylate the host genome), ‘Modification patterns’ indicates the count of unique modification patterns where >95% of motifs in the respective genome are modified. ‘P value’ indicates the P value derived from a Poisson generalized linear model (GLM) testing whether the per genome counts of ‘Total Type I, II, and III RM’ and ‘Modification patterns’ are statistically different for the respective species (with false discovery rate correction for multiple testing applied). ‘Estimate’ indicates the regression coefficient from the Poisson GLM on the log scale, representing the direction and magnitude of the difference between counts. ‘Lower CI’ and ‘Upper CI’ indicate the bounds of the 95% confidence interval for the estimate on the log scale. The data underlying this Table are available via Zenodo: https://doi.org/10.5281/zenodo.19387437. Table B in S1 Appendix. Statistics output from the Bayesian models utilized in the phylogenetically controlled within-species analyses. ‘Estimate’ denotes the median value drawn from the posterior distribution of the effect of the explanatory variable (Effect) on the response variable (ARG count per genome). ‘lwr 95% CI’ denotes the lower 95% credibility interval of the estimate, ‘upr 95% CI’ denotes the upper 95% credibility interval of the estimate, R-hat is a quantification of the Markov chain Monte Carlo chain convergence, Bulk ESS is the effective sample size for rank normalized values using split chains, Tail ESS is the minimum of the effective sample sizes for 5% and 95% quantiles. The data underlying this Table are available via Zenodo: https://doi.org/10.5281/zenodo.19387437. Table C in S1 Appendix. Shannon [file pbio.3003842.s016.docx]

S1 Appendix: Eco-evolutionary feedbacks drive the co-occurrence of restriction-modification systems and antimicrobial resistance genes in bacteria: Supplementary Information

**Table A.** Summary data for the genomes used to verify our RM system detection pipeline. ‘Genomes’ indicate the count of genomes used, ‘Type X RM systems’ indicates the total count of annotated RM systems of the respective type, ‘Total Type I, II, & III RM’ indicates the sum of Type I-III RM systems (those that methylate the host genome), ‘Modification patterns’ indicates the count of unique modification patterns where >95% of motifs in the respective genome are modified. ‘*P* value’ indicates the *P* value derived from a Poisson generalised linear model (GLM) testing whether the per genome counts of ‘Total Type I, II, & III RM’ and ‘Modification patterns’ are statistically different for the respective species (with false discovery rate correction for multiple testing applied). ‘Estimate’ indicates the regression coefficient from the Poisson GLM on the log scale, representing the direction and magnitude of the difference between counts. ‘Lower CI’ and ‘Upper CI’ indicate the bounds of the 95% confidence interval for the estimate on the log scale. The data underlying this Table are available via Zenodo: https://doi.org/10.5281/zenodo.19387437.

| Species | Genomes | Type I RM systems | Type II RM systems | Type III RM systems | Type IV RM systems | Total Type I, II, & III RM | Modification patterns | *P* value | Estimate | Lower CI | Upper CI |
| --- | --- | --- | --- | --- | --- | --- | --- | --- | --- | --- | --- |
| *A. baumannii* | 11 | 14 | 1 | 0 | 0 | 15 | 23 | 0.389 | < 0.001 | -1.826 | 0.371 |
| *C. difficile* | 10 | 12 | 2 | 0 | 0 | 14 | 21 | 0.205 | < 0.001 | -2.566 | 0.116 |
| *C. jejuni* | 13 | 15 | 31 | 3 | 0 | 49 | 63 | 0.389 | < 0.001 | -2.672 | 0.519 |
| *E. coli* | 241 | 239 | 295 | 45 | 77 | 579 | 575 | 0.859 | < 0.001 | -0.380 | 0.185 |
| *E. faecium* | 6 | 6 | 0 | 1 | 0 | 7 | 5 | 0.977 | < 0.001 | -1.479 | 1.312 |
| *H. pylori* | 9 | 17 | 70 | 14 | 0 | 101 | 134 | 0.119 | < 0.001 | -7.005 | -0.328 |
| *K. pneumoniae* | 129 | 129 | 172 | 26 | 4 | 327 | 302 | 0.965 | 0.080 | -0.310 | 0.470 |
| *N. gonorrhoeae* | 10 | 10 | 76 | 9 | 0 | 95 | 22 | < 0.001 | 7.300 | 5.180 | 9.420 |
| *P. aeruginosa* | 13 | 19 | 3 | 1 | 0 | 23 | 32 | 0.119 | < 0.001 | -2.754 | -0.107 |
| *S. aureus* | 48 | 5 | 8 | 0 | 44 | 13 | 83 | < 0.001 | < 0.001 | -2.047 | -1.184 |
| *S. enterica* | 69 | 62 | 86 | 71 | 26 | 219 | 222 | 0.976 | < 0.001 | -0.692 | 0.510 |
| *S. flexneri* | 16 | 16 | 16 | 0 | 0 | 32 | 31 | 0.977 | 0.062 | -0.910 | 1.035 |
| *S. pyogenes* | 4 | 4 | 0 | 0 | 0 | 4 | 4 | 1.000 | < 0.001 | -1.386 | 1.386 |
| *S. sonnei* | 5 | 0 | 13 | 0 | 0 | 13 | 8 | 0.859 | 0.600 | -1.379 | 2.579 |

**Table B.** Statistics output from the Bayesian models utilised in the phylogenetically controlled within-species analyses. ‘Estimate’ denotes the median value drawn from the posterior distribution of the effect of the explanatory variable (Effect) on the response variable (ARG count per genome). ‘lwr 95% CI’ denotes the lower 95% credibility interval of the estimate, ‘upr 95% CI’ denotes the upper 95% credibility interval of the estimate, R-hat is a quantification of the Markov chain Monte Carlo chain convergence, Bulk ESS is the effective sample size for rank normalized values using split chains, Tail ESS is the minimum of the effective sample sizes for 5% and 95% quantiles. The data underlying this Table are available via Zenodo: https://doi.org/10.5281/zenodo.19387437.

| Model | Effect | Estimate | lwr 95% CI | upr 95% CI | R-hat | Bulk ESS | Tail ESS |
| --- | --- | --- | --- | --- | --- | --- | --- |
| *P. aeruginosa* | Intercept | -2.135 | -2.577 | -1.707 | 1.003 | 5,862 | 2,753 |
| *P. aeruginosa* | RM count | 0.05711 | 0.039 | 0.075 | 1.001 | 7,416 | 3,076 |
| *P. aeruginosa* | Genome length (mb) | 0.5892 | 0.531 | 0.647 | 1.002 | 7,828 | 3,079 |
| *P. aeruginosa* | Phylogeny | 0.2691 | 0.246 | 0.293 | 1.001 | 3,603 | 3,486 |
| *A. baumannii* | Intercept | -1.47 | -2.375 | -0.539 | 1.001 | 2,093 | 2,473 |
| *A. baumannii* | RM count | 0.06892 | 0.030 | 0.109 | 1.001 | 4,173 | 3,518 |
| *A. baumannii* | Genome length (mb) | 0.7375 | 0.626 | 0.846 | 1.000 | 4,682 | 3,515 |
| *A. baumannii* | Phylogeny | 0.5305 | 0.494 | 0.568 | 1.001 | 2,067 | 2,958 |
| *E. faecium* | Intercept | -0.5981 | -1.156 | -0.057 | 1.000 | 4,077 | 3,204 |
| *E. faecium* | RM count | 0.04608 | 0.008 | 0.083 | 1.000 | 7,153 | 3,082 |
| *E. faecium* | Genome length (mb) | 0.6829 | 0.546 | 0.822 | 1.001 | 7,128 | 3,007 |
| *E. faecium* | Phylogeny | 0.4891 | 0.441 | 0.541 | 1.000 | 2,022 | 2,825 |
| *S. pyogenes* | Intercept | -9.622 | -12.436 | -6.936 | 1.000 | 5,164 | 3,189 |
| *S. pyogenes* | RM count | 0.9411 | 0.557 | 1.337 | 1.002 | 3,918 | 3,206 |
| *S. pyogenes* | Genome length (mb) | 5.046 | 3.581 | 6.512 | 1.000 | 5,255 | 3,001 |
| *S. pyogenes* | Phylogeny | 2.102 | 1.858 | 2.381 | 1.001 | 1,997 | 2,885 |
| *N. gonorrhoeae* | Intercept | -1.917 | -5.589 | 1.858 | 1.001 | 7,315 | 3,153 |
| *N. gonorrhoeae* | RM count | 0.2002 | -0.148 | 0.561 | 1.000 | 5,863 | 3,578 |
| *N. gonorrhoeae* | Genome length (mb) | 0.1639 | -0.732 | 1.061 | 1.003 | 13,216 | 2,748 |
| *N. gonorrhoeae* | Phylogeny | 2.191 | 1.680 | 2.814 | 1.000 | 2,586 | 3,391 |
| *All taxa random effect on species* | Intercept | 0.9793 | 0.032 | 2.110 | 1.008 | 531 | 621 |
| *All taxa random effect on species* | RM count | 0.07413 | 0.057 | 0.091 | 1.001 | 1,549 | 1,621 |
| *All taxa random effect on species* | Species identity | 2.028 | 1.362 | 3.104 | 1.006 | 486 | 917 |
| *All taxa no random effect on species* | Intercept | 2.16 | 2.144 | 2.175 | 1.001 | 4,256 | 3,292 |
| *All taxa no random effect on species* | RM count | -0.4218 | -0.433 | -0.411 | 1.001 | 1,175 | 1,487 |

**Table C.** Shannon diversity indices for the RM system content of each species, and the P value for the Hutcheson t-test conducted to test if the respective species’ diversity index is significantly lower than the Shannon diversity index of all species combined. The data underlying this Table are available via Zenodo: https://doi.org/10.5281/zenodo.19387437.

| *Species* | *Shannon diversity index* | *P value* |
| --- | --- | --- |
| *Campylobacter jejuni* | 2.178 | > 0.001 |
| *Helicobacter pylori* | 2.897 | > 0.001 |
| *Pseudomonas aeruginosa* | 2.238 | > 0.001 |
| *Acinetobacter baumannii* | 0.943 | > 0.001 |
| *Neisseria gonorrhoeae* | 2.284 | > 0.001 |
| *Klebsiella pneumoniae* | 2.298 | > 0.001 |
| *Shigella flexneri* | 0.4 | > 0.001 |
| *Shigella sonnei* | 0.638 | > 0.001 |
| *Staphylococcus aureus* | 1.489 | > 0.001 |
| *Streptococcus pyogenes* | 0.374 | > 0.001 |
| *Enterococcus faecium* | 1.258 | > 0.001 |
| *Clostridioides difficile* | 1.276 | > 0.001 |
| *Mycobacterium tuberculosis* | 0.689 | > 0.001 |
| *Salmonella enterica* | 1.936 | > 0.001 |
| *All species combined* | 3.44 |  |

**Table D.** The pairwise probabilities that an HGT event between a donor of one species (rows) and a recipient of another species (columns) will be restricted by RM (due to the recipient having an RM system that the donor does not). Values indicate the mean probabilities for all RM systems present in the recipient species. ‘Conspecific donor’ indicates that the donor is the same species as the recipient. The data underlying this Table are available via Zenodo: https://doi.org/10.5281/zenodo.19387437.

| *Recipient →   Donor ↓* | *Acinetobacter baumannii* | *Campylobacter jejuni* | *Clostridioides difficile* | *Enterococcus faecium* | *Helicobacter pylori* | *Klebsiella pneumoniae* | *Mycobacterium tuberculosis* | *Neisseria gonorrhoeae* | *Pseudomonas aeruginosa* | *Salmonella enterica* | *Shigella flexneri* | *Shigella sonnei* | *Staphylococcus aureus* | *Streptococcus pyogenes* |
| --- | --- | --- | --- | --- | --- | --- | --- | --- | --- | --- | --- | --- | --- | --- |
| *Acinetobacter baumannii* |  | 0.942 | 0.864 | 0.370 | 0.999 | 0.656 | 1.000 | 1.000 | 0.601 | 0.990 | 0.978 | 0.978 | 0.929 | 0.973 |
| *Campylobacter jejuni* | 0.926 |  | 0.985 | 0.932 | 0.995 | 0.963 | 1.000 | 0.999 | 0.957 | 0.998 | 0.998 | 0.998 | 0.992 | 0.996 |
| *Clostridioides difficile* | 0.857 | 0.988 |  | 0.870 | 1.000 | 0.929 | 1.000 | 1.000 | 0.917 | 0.998 | 0.995 | 0.995 | 0.985 | 0.994 |
| *Enterococcus faecium* | 0.369 | 0.946 | 0.875 |  | 0.999 | 0.685 | 1.000 | 1.000 | 0.635 | 0.991 | 0.980 | 0.980 | 0.935 | 0.975 |
| *Helicobacter pylori* | 0.951 | 0.991 | 0.990 | 0.955 |  | 0.975 | 0.999 | 0.991 | 0.971 | 0.993 | 0.998 | 0.998 | 0.995 | 0.994 |
| *Klebsiella pneumoniae* | 0.651 | 0.970 | 0.931 | 0.681 | 0.999 |  | 1.000 | 0.999 | 0.798 | 0.994 | 0.988 | 0.989 | 0.964 | 0.985 |
| *Mycobacterium tuberculosis* | 0.988 | 0.999 | 0.998 | 0.989 | 0.999 | 0.994 |  | 1.000 | 0.993 | 0.997 | 1.000 | 1.000 | 0.999 | 1.000 |
| *Neisseria gonorrhoeae* | 0.944 | 0.994 | 0.989 | 0.949 | 0.991 | 0.971 | 1.000 |  | 0.967 | 0.992 | 0.998 | 0.998 | 0.994 | 0.992 |
| *Pseudomonas aeruginosa* | 0.597 | 0.966 | 0.920 | 0.631 | 0.999 | 0.799 | 1.000 | 1.000 |  | 0.994 | 0.986 | 0.987 | 0.958 | 0.984 |
| *Salmonella enterica* | 0.975 | 0.997 | 0.995 | 0.977 | 0.994 | 0.987 | 0.997 | 0.993 | 0.985 |  | 0.998 | 0.999 | 0.997 | 0.999 |
| *Shigella flexneri* | 0.971 | 0.998 | 0.994 | 0.974 | 1.000 | 0.985 | 1.000 | 1.000 | 0.983 | 0.999 |  | 0.999 | 0.997 | 0.999 |
| *Shigella sonnei* | 0.971 | 0.998 | 0.994 | 0.974 | 1.000 | 0.986 | 1.000 | 1.000 | 0.983 | 1.000 | 0.999 |  | 0.997 | 0.999 |
| *Staphylococcus aureus* | 0.921 | 0.993 | 0.984 | 0.928 | 1.000 | 0.961 | 1.000 | 1.000 | 0.954 | 0.999 | 0.997 | 0.997 |  | 0.997 |
| *Streptococcus pyogenes* | 0.966 | 0.996 | 0.993 | 0.969 | 0.996 | 0.982 | 1.000 | 0.994 | 0.980 | 0.999 | 0.999 | 0.999 | 0.997 |  |
| *Conspecific donor* | 0.310 | 0.986 | 0.967 | 0.423 | 0.971 | 0.825 | 0.987 | 0.939 | 0.766 | 0.990 | 0.992 | 0.992 | 0.987 | 0.991 |


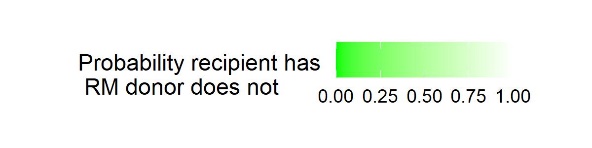


**Table E**. Jaccard indices of the ARG repertoire for each pair of species in the dataset. Values fall between 0 and 1, where 0 indicates the respective species share no ARGs, and 1 indicates both species possess an identical repertoire of ARGs. The data underlying this Table are available via Zenodo: https://doi.org/10.5281/zenodo.19387437.

|  | *Acinetobacter baumannii* | *Campylobacter jejuni* | *Clostridioides difficile* | *Enterococcus faecium* | *Helicobacter pylori* | *Klebsiella pneumoniae* | *Mycobacterium tuberculosis* | *Neisseria gonorrhoeae* | *Pseudomonas aeruginosa* | *Salmonella enterica* | *Shigella flexneri* | *Shigella sonnei* | *Staphylococcus aureus* | *Streptococcus pyogenes* |
| --- | --- | --- | --- | --- | --- | --- | --- | --- | --- | --- | --- | --- | --- | --- |
| *Acinetobacter baumannii* |  |  |  |  |  |  |  |  |  |  |  |  |  |  |
| *Campylobacter jejuni* | 0.007 |  |  |  |  |  |  |  |  |  |  |  |  |  |
| *Clostridioides difficile* | 0.011 | 0.146 |  |  |  |  |  |  |  |  |  |  |  |  |
| *Enterococcus faecium* | 0.011 | 0.114 | 0.160 |  |  |  |  |  |  |  |  |  |  |  |
| *Helicobacter pylori* | 0.013 | 0.050 | 0.070 | 0.021 |  |  |  |  |  |  |  |  |  |  |
| *Klebsiella pneumoniae* | 0.129 | 0.019 | 0.028 | 0.044 | 0.016 |  |  |  |  |  |  |  |  |  |
| *Mycobacterium tuberculosis* | 0.010 | 0.015 | 0.049 | 0.031 | 0.133 | 0.010 |  |  |  |  |  |  |  |  |
| *Neisseria gonorrhoeae* | 0.005 | 0.017 | 0.035 | 0.021 | 0.091 | 0.010 | 0.071 |  |  |  |  |  |  |  |
| *Pseudomonas aeruginosa* | 0.117 | 0.011 | 0.016 | 0.017 | 0.013 | 0.189 | 0.008 | 0.008 |  |  |  |  |  |  |
| *Salmonella enterica* | 0.136 | 0.023 | 0.042 | 0.036 | 0.023 | 0.335 | 0.017 | 0.024 | 0.186 |  |  |  |  |  |
| *Shigella flexneri* | 0.081 | 0.008 | 0.017 | 0.013 | 0.014 | 0.130 | 0.014 | 0.015 | 0.080 | 0.275 |  |  |  |  |
| *Shigella sonnei* | 0.104 | 0.007 | 0.022 | 0.023 | 0.022 | 0.184 | 0.021 | 0.045 | 0.113 | 0.360 | 0.411 |  |  |  |
| *Staphylococcus aureus* | 0.017 | 0.090 | 0.101 | 0.282 | 0.050 | 0.032 | 0.048 | 0.038 | 0.034 | 0.042 | 0.029 | 0.038 |  |  |
| *Streptococcus pyogenes* | 0.010 | 0.125 | 0.197 | 0.172 | 0.067 | 0.023 | 0.061 | 0.154 | 0.015 | 0.032 | 0.035 | 0.046 | 0.214 |  |


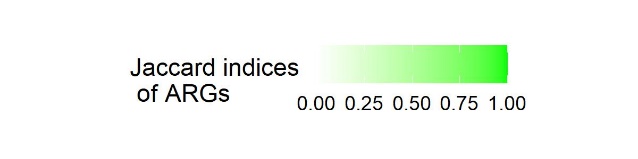


**Table F**. Pairs of RM systems and ARGs that co-occur significantly more than chance (using Bonferroni corrected alpha values), where the effect size of the co-occurrence is large (observed co-occurrence >= 10* expected co-occurrence) and excluding rare pairs that co-occur in fewer than 0.5% of the respective species’ genomes. The data underlying this Table are available via Zenodo: https://doi.org/10.5281/zenodo.19387437.

| Species | RM hmm name | ARG name | RM system n | ARG n | obs/exp | P | ARG description | resistance | mean proximity (bp) | proportion on same contig |
| --- | --- | --- | --- | --- | --- | --- | --- | --- | --- | --- |
| *S. pyogenes* | REase_II_00151 | mef(A) | 19 | 19 | Inf | 7.41e-29 | macrolide efflux MFS transporter Mef(A) | macrolide | 9,257 | 1.00 |
| *S. pyogenes* | REase_II_00151 | msr(D) | 19 | 19 | Inf | 7.41e-29 | ABC-F type ribosomal protection protein Msr(D) | macrolide | 7,756 | 1.00 |
| *P. aeruginosa* | REase_I_00002 | dfrB5 | 84 | 84 | 46.00 | 2.71e-51 | trimethoprim-resistant dihydrofolate reductase DfrB5 | trimethoprim | 1,005,859 | 0.26 |
| *P. aeruginosa* | REase_I_00002 | blaOXA-4 | 84 | 51 | 45.00 | 2.64e-59 | carbapenem-hydrolyzing class D beta-lactamase OXA-4 | carbapenem | 1,648,450 | 0.16 |
| *P. aeruginosa* | REase_I_00002 | aac(3)-Id | 84 | 75 | 44.00 | 2.07e-50 | aminoglycoside N-acetyltransferase AAC(3)-Id | gentamicin | 1,090,488 | 0.25 |
| *A. baumanii* | REase_I_00005 | blaADC-182 | 80 | 52 | 40.00 | 1.97e-48 | class C beta-lactamase ADC-182 | cephalosporin | 388,574 | 0.22 |
| *P. aeruginosa* | REase_I_00003 | blaPDC-36 | 59 | 42 | 40.00 | 3.04e-60 | class C beta-lactamase PDC-36 | cephalosporin | 1,918,323 | 0.12 |
| *P. aeruginosa* | REase_I_00002 | cmlA6 | 84 | 51 | 40.00 | 6.82e-51 | chloramphenicol efflux MFS transporter CmlA6 | chloramphenicol | 2,100,532 | 0.02 |
| *A. baumanii* | REase_I_00005 | blaTEM-150 | 80 | 41 | 34.00 | 1.72e-42 | class A beta-lactamase TEM-150 | beta-lactam | 2,060,794 | 0.09 |
| *P. aeruginosa* | REase_MTase_IIG_00002 | blaVEB-9 | 155 | 40 | 29.00 | 6.60e-29 | class A extended-spectrum beta-lactamase VEB-9 | cephalosporin | 158,144 | 0.48 |
| *A. baumanii* | REase_I_00005 | blaOXA-51 | 80 | 56 | 27.00 | 6.59e-28 | OXA-51 family carbapenem-hydrolyzing class D beta-lactamase OXA-51 | carbapenem | 853,974 | 0.04 |
| *P. aeruginosa* | REase_MTase_IIG_00002 | dfrB2 | 155 | 30 | 26.00 | 5.14e-28 | trimethoprim-resistant dihydrofolate reductase DfrB2 | trimethoprim | 144,640 | 0.54 |
| *P. aeruginosa* | REase_I_00002 | aph(3')-XV | 84 | 64 | 25.00 | 4.46e-25 | aminoglycoside O-phosphotransferase APH(3')-XV | amikacin kanamycin | 5,298,421 | 0.16 |
| *P. aeruginosa* | REase_II_00142 | aac(6')-29a | 225 | 51 | 24.50 | 7.04e-46 | aminoglycoside N-acetyltransferase AAC(6')-29a | aminoglycoside | 1,293,581 | 0.04 |
| *P. aeruginosa* | REase_I_00002 | aadA2 | 84 | 137 | 22.50 | 1.24e-40 | ANT(3'')-Ia family aminoglycoside nucleotidyltransferase AadA2 | streptomycin | 975,494 | 0.27 |
| *P. aeruginosa* | REase_I_00009 | blaOXA-56 | 281 | 42 | 20.00 | 5.94e-34 | OXA-10 family oxacillin-hydrolyzing class D beta-lactamase OXA-56 | beta-lactam | 3,737,765 | 0.15 |
| *P. aeruginosa* | REase_I_00002 | tet(G) | 84 | 192 | 19.33 | 6.64e-50 | tetracycline efflux MFS transporter Tet(G) | tetracycline | 3,155,011 | 0.28 |
| *P. aeruginosa* | REase_II_00142 | blaOXA-9 | 225 | 38 | 19.00 | 1.42e-36 | oxacillin-hydrolyzing class D beta-lactamase OXA-9 | beta-lactam | 3,154,473 | 0.28 |
| *P. aeruginosa* | REase_I_00002 | floR2 | 84 | 177 | 19.00 | 1.44e-50 | chloramphenicol/florfenicol efflux MFS transporter FloR2 | chloramphenicol florfenicol | 3,229,409 | 0.08 |
| *P. aeruginosa* | REase_II_00142 | cmlB | 225 | 37 | 18.50 | 1.18e-35 | chloramphenicol efflux MFS transporter CmlB1 | chloramphenicol | 3,594,717 | 0.05 |
| *A. baumanii* | REase_I_00005 | dfrA1 | 80 | 100 | 18.00 | 2.52e-32 | trimethoprim-resistant dihydrofolate reductase DfrA14 | trimethoprim | 2,062,830 | 0.08 |
| *A. baumanii* | REase_I_00005 | sat2_gen | 80 | 92 | 18.00 | 1.46e-33 | streptothricin N-acetyltransferase Sat2 | streptothricin | 2,062,606 | 0.08 |
| *P. aeruginosa* | REase_II_00142 | aac(6')-29b | 225 | 56 | 17.67 | 3.68e-49 | aminoglycoside N-acetyltransferase AAC(6')-29b | aminoglycoside | 2,566,367 | 0.02 |
| *P. aeruginosa* | REase_MTase_IIG_00002 | blaOXA-846 | 155 | 108 | 16.50 | 4.67e-59 | OXA-50 family oxacillin-hydrolyzing class D beta-lactamase OXA-846 | beta-lactam | 1,040,772 | 0.76 |
| *P. aeruginosa* | REase_I_00009 | rmtD1 | 281 | 31 | 15.00 | 4.62e-26 | 16S rRNA (guanine(1405)-N(7))-methyltransferase RmtD1 | aminoglycoside | 3,831,774 | 0.13 |
| *P. aeruginosa* | REase_I_00009 | blaSPM-1 | 281 | 28 | 14.00 | 8.33e-25 | subclass B1 metallo-beta-lactamase SPM-1 | carbapenem | 3,630,133 | 0.25 |
| *P. aeruginosa* | REase_I_00009 | blaVEB-9 | 281 | 40 | 13.00 | 1.26e-18 | class A extended-spectrum beta-lactamase VEB-9 | cephalosporin | 2,312,364 | 0.58 |
| *P. aeruginosa* | REase_II_00142 | blaCARB-2 | 225 | 76 | 12.75 | 3.42e-40 | PSE family carbenicillin-hydrolyzing class A beta-lactamase CARB-2 | beta-lactam | 786,362 | 0.06 |
| *P. aeruginosa* | REase_I_00009 | aac(3)-Ic | 281 | 36 | 12.50 | 1.26e-18 | aminoglycoside N-acetyltransferase AAC(3)-Ic | gentamicin | 2,040,155 | 0.16 |
| *A. baumanii* | REase_I_00009 | blaADC-191 | 187 | 37 | 12.50 | 1.09e-20 | class C beta-lactamase ADC-191 | cephalosporin | 2,676,636 | 0.04 |
| *A. baumanii* | REase_I_00008 | blaADC-222 | 237 | 30 | 12.00 | 2.91e-19 | class C beta-lactamase ADC-222 | cephalosporin | 757,849 | 0.08 |
| *A. baumanii* | REase_I_00008 | blaOXA-95 | 237 | 29 | 12.00 | 1.36e-19 | OXA-51 family carbapenem-hydrolyzing class D beta-lactamase OXA-95 | carbapenem | 2,146,325 | 0.08 |
| *A. baumanii* | REase_I_00008 | blaADC-26 | 237 | 108 | 11.83 | 2.62e-48 | class C extended-spectrum beta-lactamase ADC-26 | cephalosporin | 2,096,475 | 0.07 |
| *P. aeruginosa* | REase_MTase_IIG_00003 | blaPDC-34 | 397 | 183 | 11.80 | 1.85e-121 | class C beta-lactamase PDC-34 | cephalosporin | 800,446 | 0.08 |
| *P. aeruginosa* | REase_I_00002 | aac(6')-Il | 84 | 213 | 11.75 | 9.15e-35 | aminoglycoside N-acetyltransferase AAC(6')-Il | amikacin;kanamycin tobramycin | 1,006,579 | 0.26 |
| *A. baumanii* | REase_I_00005 | blaOXA-65 | 80 | 129 | 11.33 | 2.65e-26 | OXA-51 family carbapenem-hydrolyzing class D beta-lactamase OXA-65 | carbapenem | 786,501 | 0.03 |
| *P. aeruginosa* | REase_I_00002 | blaVIM-2 | 84 | 231 | 11.25 | 4.04e-31 | subclass B1 metallo-beta-lactamase VIM-2 | carbapenem | 594,149 | 0.16 |
| *P. aeruginosa* | REase_MTase_IIG_00002 | tet(A) | 155 | 84 | 11.00 | 2.04e-24 | tetracycline efflux MFS transporter Tet(A) | tetracycline | 155,749 | 0.36 |
| *P. aeruginosa* | REase_III_00001 | blaGES-14 | 347 | 36 | 10.33 | 9.01e-23 | carbapenem-hydrolyzing class A beta-lactamase GES-14 | carbapenem | 20,557 | 0.19 |
| *P. aeruginosa* | REase_I_00009 | aadA7 | 281 | 60 | 10.25 | 2.86e-29 | ANT(3'')-Ia family aminoglycoside nucleotidyltransferase AadA7 | streptomycin | 3,737,764 | 0.15 |
| *A. baumanii* | REase_I_00005 | cmlB1 | 80 | 176 | 10.25 | 2.31e-29 | chloramphenicol efflux MFS transporter CmlB1 | chloramphenicol | 899,322 | 0.02 |
