# Supplementary material for: Eco-evolutionary feedbacks drive the co-occurrence of restriction-modification systems and antimicrobial resistance genes in bacteria
Source: PLoS Biol. 2026 Jun 15;24(6):e3003842. doi: 10.1371/journal.pbio.3003842 (PMC13293514; doi:10.1371/journal.pbio.3003842)
Supplement: Attachment — Submitted filename: Responses_to_reviewers.docx [file pbio.3003842.s019.docx]

EDITOR’S SUMMARY

In light of the reviews, which you will find at the end of this email, we would like to invite you to revise the work to thoroughly address the reviewers' reports. As you will see below, the reviewers think the topic is important and the results potentially interesting, yet some concerns have raised during revision. Reviewer 1 mentions that the study requires clarifications and corrections, specifically regarding the model, suggesting additional simulations. Reviewer 2 thinks the study requires certain aspects to be further discussed in depth to support the conclusions. For example, the reviewer suggests to use RNAseq data to distinguish between functional and non-functional RM systems, to statistically analyse the influence of other defense systems, and additional model simulations including MGE counter-defenses. We agree with all reviewer concerns and would require some additional experimental revisions to address them, as we consider that this would strengthen the work.

IMPORTANT: after discussion with the Academic Editor, we think 1) you should respond to all points; and 2) address in some way R2's point 1 regarding assumption of RM functionality, as this is central to the validity of the study. It could be RNAseq as suggested by the reviewer, or some other suitable approach accessible to the you.

RESPONSE TO EDITOR’S SUMMARY

We would like to sincerely thank the two independent reviewers for providing valuable and constructive feedback, as well as the PLOS Biology editors and Academic editor for their role is assessing the suitability of this publication for PLOS Biology. Based on this feedback we have made many changes and additions to the initial submission, which in our opinion have significantly improved the manuscript.

To address reviewer 2’s first concern, the revised manuscript contains an analysis validating the functionality of annotated RM systems. While utilizing RNA-seq was a helpful suggestion, limitations in available datasets led us to instead leverage the wealth of information on bacterial methylomes available from REBASE. Using 584 genomes spanning all study species we revealed that the count of unique methylation signatures per genome (indicative of functional methyltransferases) was highly similar to the count of RM systems our pipeline identified (when excluding low coverage signatures unlikely to be associated with functional RM systems).

As suggested by reviewer 1, we also now present data on the relative frequencies of sub-populations for all simulations, providing important context to our original findings, and additional insights into the relative fitness of different RM investment levels across a range of ecological variables. Reviewer 1 also highlighted several instances where our description of how the modelling was implemented was unclear, which we have now rectified. As suggested by reviewer 2, we have also run several additional models where we vary the efficacy of RM substantially and show that positive associations between RM investment and MGEs occur even when escape of RM is very rare.

We now better justify our focus on pathogenic bacteria, acknowledge the limitations of this choice, and note that our findings may not fully generalize to non-pathogens. Both reviewers correctly pointed out that our statement that RM systems are more likely to pose a barrier to inter than intraspecific HGT events is confounded by encounter rate. We now clarify that we meant per potential HGT event, not in absolute terms.

Although we have implemented the majority of changes suggested by reviewers, for a small number of comments we respectfully differ from the reviewers’ assessment. We believe that trait depth remains an informative and useful metric, and we present our reasoning below. We stand by our decision to focus solely on RM systems, as their well-established mechanisms and the extensive experimental evidence they restrict HGT in the lab allows us to formulate testable hypotheses and construct meaningful mathematical models. This is currently not possible for many of the recently discovered defenses where mechanisms of action and protection ranges are often still unclear, or impact on HGT has not been demonstrated. Finally, our modelling framework is necessarily simplified in order to allow testing of our specific hypotheses. We recognize that anti-RM systems are an important component of the interplay between RM and MGEs; however, the influence of the eco-evolutionary role of anti-RM remains beyond the scope of this current manuscript.

Please see below for detailed point-by-point responses to all reviewer comments (note, all line numbers refer to the document with changes tracked). We believe the revised manuscript is a notable improvement over the initial submission and that it will be of high interest to the PLOS Biology readership.

REVIEWERS' COMMENTS
------------------------------------
Reviewer #1:

Authors test the hypothesis that stronger selection for restriction-modification (RM) systems at higher exposure to costly MGEs results in the observed higher number of MGEs in bacteria with more RM systems. Their findings come from the model simulations, on one side, and from the bioinformatics analysis of >40,000 bacterial genomes, on the other. The topic and the goals of this study are interesting and important, but at this point there are many things in the presented manuscript that need to be clarified, justified or, potentially, corrected.

***************

MODEL

(I kindly ask Authors to comment on what I didn't understand correctly, if there is anything, and to also clarify the same in the manuscript.) According to the described model, I understood that a population of bacteria consists of subpopulations characterized by all possible combinations of the following two characteristics:
i) RM system presence & influence (n denotes the scenario), which can be: (1) RM system absence with no (0) effect on the host cells reproduction rate and the likelihood of acquiring an MGE, (2) presence of a hypothetical RM system 1 with low inhibitory effect on the host cells reproduction rate and the likelihood of acquiring an MGE, (3) presence of a hypothetical RM system 2 (with a different target sequence) with high inhibitory effect on the host cells reproduction rate and the likelihood of acquiring an MGE;
ii) MGE presence (i denotes the number of different MGEs present in a cell, not the copy number of the same MGE), where the number of MGEs in a cell can be 0, 1, 2, 3, 4 or 5.

In a compartmental (ODE) model, cell number S_n,i of a given sub-population:
1) increases by division of those cells (where the reproduction rate is negatively affected by RM system and MGEs presence);
2) increases by MGE loss in Sn,i+1 cells (with the same RM system; one MGE is lost completely, not transferred);
3) increases by MGE gain in Sn,i-1 cells (where methylated and unmethylated MGEs are gained with different rates);
4) decreases by cell death (complete elimination from the population);
5) decreases by MGE loss (one MGE is lost completely);
6) decreases by MGE gain (where methylated and unmethylated MGEs are gained with different rates).

I suggest adding a similar conceptual description of the modelled population in the main text, or providing in another way (maybe with and illustration) this information that is needed for the interpretation and understanding of the results.

Regarding point i) the reviewer has understood correctly the structure of the sub-populations, and regarding point ii) the reviewer has understood correctly that i denotes the number of MGEs carried, not the copy number of a single MGE. MGE copy number is not explicitly included in our model; instead we assume that if a cell has an MGE, it can transfer it to another cell without loss of the MGE.

We thank the reviewer for the clear and concise description of the factors that influence the change in size of a given sub-population. We have included a similar conceptual description in the Mathematical modelling methods section of the main text (see lines 424-426 main text).

It seems that the terms corresponding to rates under 3) and 5) are missing from the model (eq. in Supplementary document, lines 40-42)?

This error has been corrected.

(Supplementary document S2.1, 1st paragraph) I assume it should read 'i' MGEs and having RM system 'n'.

This error has been corrected.

Why did you choose this particular dependence of the reproduction rate on the number and costs of MGEs? The strong nonlinear decay of this rate with the number of MGEs needs additional justification.

We model the costs associated with MGE carriage as a proportional reduction in host growth rate (a multiplicative model). Here, the addition of an MGE reduces the remaining growth potential by a fixed percentage, rather than by reducing by an absolute value. Such multiplicative modelling of the costs incurred by MGE carriage is appropriate under exponential growth (1). Multiplicative modelling of costs also prevents the occurrence of negative growth rates, which could occur when using an additive approach. We have added justification for this approach to the main text (see lines 456-459).

(Main text, L369) Here authors state that cells in the model can acquire 5 "identical" MGEs and this needs to be clarified. Are different MGEs/ARGs considered, which can also replicate in the host cell, so for another cell to acquire an MGE, it is not needed that the first cell looses its MGE? Or are the copy numbers of the same MGE considered? In that case, dynamics of the MGE number should probably also be described by the model.

We agree that this is an important point and the text required clarification. The five MGEs that can be gained are identical in the fitness costs they impose on their host, but they should be considered distinct MGEs, not copies of the same MGE. The copy number of these individual, distinct MGEs is not explicitly modelled, and a cell cannot increase the count of MGEs it carries through MGE replication within the cell. Cells gain MGEs only through horizontal transfer from another cell with an MGE. Importantly, this horizontal transfer does not lead to the loss of an MGE in the donor cell. We have added some additional text to improve clarity on these aspects of the modelling approach (see lines 425-428 main text).

(Main text, L160-166) Please explain more directly how you define and vary the population level RM diversity in the model. By setting initial conditions so that there are different numbers of cells with 0, low and high RM investment and examining the state of the system at the end of a simulation?

We have revised the text in the Mathematical modelling methods section to more clearly explain how we have implemented increasing RM diversity in our simulations (see lines 469-476 main text).

In the low RM diversity simulations, the population consists of three subpopulations differing in their investment in RM (zero, low, and high). HGT between cells with the same RM investment level is unrestricted, as the transferred MGE carries the matching methylation signature, corresponding to a population level RM richness of two (one high investment and one low investment RM system).

In the high RM diversity simulations, we modelled the effect of increased RM richness without explicitly adding more sub-populations (as this would make the model intractable). To do this, we introduced a parameter controlling the probability that an MGE bears the appropriate protective methylation signature during HGT between cells with the same RM investment level. This probability is 1 in low-diversity simulations (unrestricted HGT) and 0.333 in high-diversity simulations, representing an increase in RM richness from two (one low and one high investment RM system) to six (three high and three low investment RM systems).

Does the finding that "bacteria with high RM investment also had a higher mean count of MGEs" depends on which RM systems are most prevalent in the low diversity scenario? E. g. if high RM investment cells comprise the majority of the population?

This is an important point, and we would like to clarify what our simulation results are showing. Here, we are quantifying the average count of MGEs per bacterium, for each level of RM investment. Therefore, the relative abundance of sub-populations with different levels of RM investment will not impact our inference here.

However, we recognize that inclusion of the relative abundances of sub-populations at equilibrium would provide important context, particularly relating to how the fitness of different RM investment levels varies across a gradient of RM and MGE costs. We have now presented and described these data in Figure S1.2 and in lines 169-186.

I wonder if simulating the scenarios of no diversity (all cells in the population are of the same RM investment level) would be helpful in interpreting the results. Also, I would like to know what are the relative sizes of the sub-populations at the end of the simulations - e. g. is the number of MGEs that end up in no-RM cells low because these cells loose in the competition, or because they win so their number is actually large.

In our simulations, RM systems do not limit the transfer of MGEs between cells with the same level of RM investment. A recipient’s RM system only functions to limit MGE acquisition when the donor cell has no RM system, or a different RM system. When recipient and donor cells match in their RM status, the MGE will be protected from restriction by the recipient endonucleases, due to being pre-methylated by the donor methyltransferases. This means that when simulating scenarios where all cells are of the same type, RM systems are essentially non-functional, and simulations reach equilibrium with the same average count of MGEs, regardless of the RM system investment level. We ran these models to check this and found identical results regardless of which sub-population was being modelled, but we feel that the inclusion of these results would not be informative.

As previously stated, we agree with the reviewer that the relative sizes of sub-populations at the end of the simulations can provide useful context to the reader, and this data has been visualized in Figures S1.2.

In response to the reviewer’s question regarding whether the number of MGEs that end up in no-RM cells is low because these cells lose in the competition, or because they win so their number is actually large, we would like to clarify that we are plotting the average count of MGEs per individual bacterium for each level of RM investment. Therefore, the relative abundance of sub-populations of different RM investments will not impact our inference here. Analyzing our results in this way allows us to answer the specific question “How does variation in rates of HGT in different ecological contexts influence the association between RM systems and MGEs?”

(Main text, L167-169) How do the "simulations suggest high exposure to costly MGEs can drive an increased investment into RM-mediated genome defence" when cells in the model cannot change their RM status (acquire new RM systems) in response to the varying MGE costs?

This is an important point that needs clarification. Although investment into RM can indeed increase through acquisition of a novel RM system through HGT or mutation, this is not what we are modelling. Instead, we model the change in population allele frequencies. When we say “… can drive increased investment into RM-mediated genome defence” the intended meaning is that high exposure to costly MGEs can cause populations to evolve to invest more into RM mediated genome defence, as measured through a corresponding change in allele frequencies (i.e. following classical population genetics). We have clarified this in the revised manuscript (lines 153-154 main text).

Generally, "investment in RM systems" can be misleading and it could be replaced by e. g. "the cost of maintaining an active RM system". Specifically, one may conclude that cells choose if they are going to keep an RM system or not and that would need to be further discussed.

We have chosen the term “investment” as we felt that it best captured our intended meaning, specifically the link between the cost and the payoff. E.g., in our simulations increasing investment into RM increases both the costs and the benefit (i.e. efficacy of defense) provided. We are happy to change the terminology for improved clarity, but feel that “cost of maintaining an active RM system” may lead readers to incorrectly deduce that only costs, and not benefits, are varying between different sub-populations in the simulations.

***************

BIOINFORMATICS

(Main text, L234-236) Does this hypothesis assume the equal probability for all species to come into contact with each other, i. e. live together in the same environment?

Our intention here was to state that as it is less likely heterospecifics will share RM systems than conspecifics, RM systems are more likely to pose a barrier to HGT between heterospecifics, *per HGT opportunity*. We recognize this was not clear, and we have altered the stated hypothesis to make it clear this is per HGT opportunity (see line 272). We also now address the fact that HGT opportunities may occur less frequently between heterospecifics than conspecifics, and therefore in absolute terms, RM may more often function as a barrier to HGT between conspecifics (see lines 287-291).

(Main text, L313-316) If the species selected for the analysis do not share the same environment, and are therefore typically not expected to share any MGEs via HGT, as authors mention, such a dataset seems inappropriate for a statistical analysis aiming to clarify the relationship between acquired RM systems and ARGs between species ("understanding the role RM plays in regulating HGT of ARGs").

It is important to note that the quoted lines are our explanation for why we do not see sharing of ARGs between two of our study species, even when the likelihood RM systems pose a barrier to HGT between these species is low (analysis relating to Figures S8, and Tables S4-S5). This analysis highlights that factors beyond the barrier effect of RM, such as the overlap of ecological niches, will be crucial for whether HGT occurs across species boundaries. It is precisely because this analysis tempers our view of the importance of RM in regulating HGT that we feel it is a necessary inclusion in the paper.

However, we must clarify that our statement that HGT between the study species is likely limited by differences in ecology, does not detract from the key hypotheses tested in Figure 2. Here, we hypothesize that a species with more RM systems will have fewer horizontally acquired genes; this is not dependent on HGT occurring between the specific study species in our dataset *per se*. We are hypothesizing that a species with more RM systems will be less likely to acquire mobile genes from the other species in the environment it inhabits, compared with a species with fewer RM systems. There is no assumption that the HGT is occurring between the study species in this analysis. We have added a sentence to clarify this meaning (see lines 365-370).

Please add the information from Supplementary document L72-75 ("The trait depth filtered dataset"), on the dataset used in the analysis, to the Methods section in the main text.

We have moved the trait depth filtered modelling methods to the main text (see lines 620-632).

------------------------------------

Reviewer #2:

This manuscript presents a synthesis of mathematical modelling and comparative genomics to investigate the eco-evolutionary dynamics underlying the co-occurrence of restriction-modification (RM) systems and antimicrobial resistance genes (ARGs).

The authors show that population-level RM diversity critically modulates the direction of association between RM systems and horizontally acquired ARGs, offering a robust explanation for previously paradoxical genomic patterns. Their large-scale analysis of >40,000 bacterial pathogens' genomes lends empirical support to the model predictions, distinguishing between-species negative correlations from within-species positive associations. The study rules out genetic linkage as a major explanatory factor and instead highlights how imperfect barriers and shared methylation facilitate ARG dissemination among RM-compatible strains.

Overall, the work provides insight into the eco-evolutionary forces shaping genome defense and resistance, with important implications for AMR spread.

General assessment
The general results of the study very interesting, especially because it is taking into consideration eco-evolutionary interactions and infers how they influence genome architecture.

However, some major aspects need to be discussed and addressed in greater depth in order to fully justify the inferred conclusions.

 Here are some conceptual aspects (major points):

1.      Assumption of RM Functionality: The study assumes that all annotated RM systems are functional without any direct validation. Given that many RM genes are cryptic or degraded, this could overestimate their barrier effect. While bioinformatic annotations alone cannot distinguish functional RM systems from degraded or non-functional remnants, RNAseq data on a small subsample could give at least a clue about what fraction of RM systems are expressed and what are non-expressed pseudogenes.

We thank the reviewer for this useful suggestion and agree that the lack of validation of RM system functionality weakened our initial submission. As we could not readily access sufficient transcriptomic datasets for all model species, we have addressed this concern using the extensive methylome data in the REBASE PacBio database.

Specifically, we downloaded all genomes for the fourteen species used in the study for which REBASE reports unique methylation signatures (584 genomes, spanning all study species). For each genome we compared the count of unique methylation signatures, representative of unique and active methyltransferases, with the count of RM systems our pipeline detected. When including all unique methylation signatures, regardless of the proportion of the respective motifs that were modified in the genome, our pipeline identified (across all species) 0.54 RM systems per unique modification signature. As we identified fewer RM systems with our pipeline than there were unique methylation signatures, it is probable that many of these genomes possessed functional orphan methyltransferases that methylated the genome but were not identified as RM systems by our pipeline (due to lacking a cognate endonuclease). We conducted this analysis again, but we removed methylation signatures for which <50% of motifs were methylated, as these were unlikely to be associated with functional RM systems due to the autoimmunity that would arise at such low levels of genome methylation. Here our pipeline identified 0.91 RM systems per unique methylation signature.

We recognize that methyltransferase functionality does not directly translate to validation of RM system functionality, however, as endonuclease activity could not be validated at a comparable scale, we have treated this evidence of methyltransferase activity as a practical if imperfect proxy for RM system functionality. The high similarity between RM systems detected and unique methylation signatures per genome (when considering signatures with >50% motifs modified) provides additional confidence that we are not notably under or overestimating the count of RM systems per genome with our detection pipeline. For the results of this analysis see lines 215-233 and Figure S3, and for the complete methods see lines 514-524.

2.      Trait Depth Inference: The use of trait depth as a proxy for acquisition timing lacks resolution and may not reliably disentangle the temporal order of RM and ARG acquisition, especially in recombining lineages. Trait depth is inferred from phylogenetic distribution and assumes vertical inheritance, but in recombining lineages, horizontal gene transfer can blur this signal. If either RM systems or ARGs have been frequently gained or lost through recombination, their phylogenetic placement may not reflect true acquisition timing, leading to inaccurate inferences about which trait arose first. A recent study ([https://eur03.safelinks.protection.outlook.com/?url=https%3A%2F%2Fdoi.org%2F10.1073%2Fpnas.2316302121&data=05%7C02%7Cjw1235%40exeter.ac.uk%7Cb47c800d906646650b3108ddc52d53c2%7C912a5d77fb984eeeaf321334d8f04a53%7C0%7C0%7C638883520644449163%7CUnknown%7CTWFpbGZsb3d8eyJFbXB0eU1hcGkiOnRydWUsIlYiOiIwLjAuMDAwMCIsIlAiOiJXaW4zMiIsIkFOIjoiTWFpbCIsIldUIjoyfQ%3D%3D%7C0%7C%7C%7C&sdata=MES%2FsFs%2F%2F3MoOwBsQvx0n97yCTF1JIlN2iTBTWxdzbY%3D&reserved=0](https://eur03.safelinks.protection.outlook.com/?url=https%3A%2F%2Fdoi.org%2F10.1073%2Fpnas.2316302121&data=05|02|jw1235@exeter.ac.uk|b47c800d906646650b3108ddc52d53c2|912a5d77fb984eeeaf321334d8f04a53|0|0|638883520644449163|Unknown|TWFpbGZsb3d8eyJFbXB0eU1hcGkiOnRydWUsIlYiOiIwLjAuMDAwMCIsIlAiOiJXaW4zMiIsIkFOIjoiTWFpbCIsIldUIjoyfQ%3D%3D|0|||&sdata=MES%2FsFs%2F%2F3MoOwBsQvx0n97yCTF1JIlN2iTBTWxdzbY%3D&reserved=0)) showed that in many of the analyzed species the rate of homologous recombination is exceptionally high, indicating that the assumption of solely vertical inheritance is an oversimplification.

We appreciate the reviewer’s points regarding the limitations of using trait depth to discern the order of acquisition of RM and ARGs. However, we respectfully argue that, despite the limitations, trait depth remains an informative metric for this analysis.

The concern that trait depth is blurred by recombination between lineages is valid; however, this will be occurring for both ARGs and RM systems, meaning that comparisons remain useful. The referenced paper does provide evidence of high levels of homologous recombination in many bacterial species, and this is likely to complicate our ability to reconstruct genomic evolutionary histories with very high degrees of precision. However, it does not offer any evidence that suggests the trait depth of genes that can mobilize is a meaningless metric.

Furthermore, here we are calculating the mean trait depth of a specific RM system or ARG, rather than calculating trait depth of the trait in question at every point it appears within a tree. In order for a clade to be considered positive for a trait, we require >90% of the tips in the clade to have the trait. This means that for us to erroneously conclude ‘clade Y is positive for trait X due to vertical inheritance’, homologous recombination would have to occur between >90% of the tips in clade Y. This is possible when such homologous recombination or HGT events occur with a very high frequency; however, if this is the case, it would also be likely that homologous recombination would cause trait X to be transferred outside of clade Y, lowering the mean trait depth (when <90% of a clade is positive for a trait, each occurrence is recorded as having a trait depth of half the distance from the tip to closest node).

Being able to reliably infer the phylogenetic relationships of lineages and evolutionary histories of traits in taxa that experience frequent horizontal gene flow presents a longstanding and complex problem for those studying genomic evolution. We recognize that the use of trait depth has limitations, and is an imperfect approximation of when traits were acquired for taxa with high levels of HGT. However, we stand by our use of trait depth to understand the likelihood one trait was acquired before another in a lineage’s evolutionary history.

3.      Limited Consideration of Other Defense Systems: The analysis focuses exclusively on RM systems, ignoring potentially confounding or interacting effects from co-occurring systems like CRISPR-Cas or abortive infection systems. Since other systems have also been annotated (Fig. S2), a statistical analysis of their influence on the probability of ARGs could also be included and would vastly improve the impact of the paper (even if no significant effect is found).

We recognize it is likely that beyond RM, other defenses will shape the coevolution of bacteria and MGEs. However, we have chosen to focus solely on RM for three key reasons. Firstly, we have a clear mechanistic understanding of how RM systems regulate many forms of HGT, as well as a large body of experimental evidence demonstrating these effects in the laboratory (although we also have this for CRISPR-Cas, bioinformatic analysis of the relationship between MGEs and CRISPR has already been thoroughly explored (2)). For many more recently discovered defense systems, it is unclear exactly how resistance is conferred, which signals of infection trigger the defense phenotype, and which categories of MGE they provide resistance against. Many bacterial defense systems, including many abortive infection systems, are known to be triggered by detection of phage proteins/structural components (3–6), which means they are unlikely to act against non-phage MGEs, and thus are unlikely to regulate HGT beyond transduction. For defenses where mechanism or breadth of resistance is unknown, it would be difficult to usefully interpret the results of any bioinformatic analyses that attempt to reveal whether they influence acquisition of mobile genes. Secondly, as we understand how RM systems work and how MGEs can escape them, we are able to develop mathematical models that can provide insight into defense-MGE eco-evolutionary dynamics. This is not possible for defenses for which we are lacking a clear mechanistic understanding, or experimental validation of the breadth of resistance. Thirdly, as evidenced by our analysis in Figure S2, RM systems are the most abundant bacterial defense system across our study species. We believe that this ubiquity, combined with their ability to restrict infection of many diverse forms of non-self-DNA, makes it likely that they are a particularly important defense system for bacteria-MGE coevolution.

We recognize that by focusing solely on RM, we are not providing a complete picture of how the defensome regulates the coevolution of bacteria and MGEs. However, this is a tradeoff we make willingly, in order to more fully investigate the complex eco-evolutionary dynamics that unfold between RM carrying bacteria, and mobile genetic elements, outside of the laboratory.

4.      Simplified Modeling Framework: The mathematical model does not include key MGE counter-defenses (e.g., anti-restriction systems), which limits its capacity to fully capture real-world eco-evolutionary dynamics. While the authors speculate about their potential impact, some qualitative example simulations of a model, which includes them would be highly beneficial. Also, showing prevalence of anti-restriction systems and RM systems would imply something about their evolutionary importance.

First, in our modelling framework, we have modelled the effect of varying RM efficacy (this implicitly models the effect of anti-RM, since they cause RM efficacy to be reduced). This has been carried out in response to this and other feedback we received, and has allowed us to better understand the relationship between defense investment and MGE count (see Figure S1.3).

Explicitly modelling anti-RM systems would go beyond the scope of this study since it is not required to address our key aim, which is to test specific hypotheses of how RM cost, diversity, and efficacy, combined with variation in exposure to HGT, can drive associations, and dissociations, between RM defense systems and mobile genes. Hence, explicit inclusion of anti-RM would not be well justified. Moreover, doing so would increase the model complexity, reducing its tractability and therefore our ability to obtain a meaningful understanding of the causal relationships of interest. We feel that future studies that aim to explore the evolution of anti-RM activity and their impact on the eco-evolutionary dynamics of RM-MGE interactions would be better suited to add this layer of complexity to our or alternative models.

We acknowledge that it would be possible to identify anti-RM systems in the genomic data, using a tool like AntiDefenseFinder (7). However, we have chosen not to pursue this because the scale of available data on anti-RM is much lower than for RM; AntiDefenseFinder currently contains hidden Markov models for 26 anti-RM genes, while PADLOC contains hidden Markov models for 268 endonucleases and 251 methyltransferases. As this order of magnitude difference likely reflects a bias in available data, rather than a real-world bias in ubiquity of RM vs anti-RM, we believe our false negative rate for anti-RM identification would be too high for us to draw meaningful inferences. Anti-defence genes (including anti-RM) are being discovered at a high rate, and we expect that it will be possible in the near future to consider these genes in bioinformatics analyses to better understand how they shape bacterial genome evolution.

5.      Bacterial pathogens only: Pathogenic life style is ecologically very distinct from other non-pathogenic life styles and often shows specific genomic signatures (pathogenicity islands carrying virulence genes, genome reduction, elevated rates of HGT, proliferation of IS elements, pseudogene accumulation, altered GC-content… ). I understand that spread of ARGs in bacterial pathogens is of high medical importance, however in my opinion it deserves explanation in the introduction (not just Materials and Methods). Furthermore, the results should be interpreted more carefully because the findings may not hold for non-pathogenic bacteria.

We agree that the decision to focus solely on bacterial pathogens requires justification in the introduction, and that we should better acknowledge that this may limit the extent to which our results are generalizable to non-pathogens. This has now been included, see lines 123-126 of the Introduction, and lines 355-361 of the Conclusion and outlook.

And some minor comments:

L109 I would strongly suggest using other wording of the sentence and avoid putting the word imperfect in the brackets. It blurs the intention of the authors.

Wording has been changed to improve clarity.

L119 I agree that RM systems can influence homologous recombination rates in Neisseria meningitidis. However, the relevance of the statement here seems to me rather questionable. The paper focusses on HGT of MGEs and suddenly introduces homologous recombination. Is it a special case of homologous recombination within MGEs? If so, please explain. If not, I would leave the sentence out.

This was originally included as homologous recombination between MGEs and the chromosome is relevant to how genetic material move horizontally, however, we have removed the sentence as we agree this concept isn’t central to the hypotheses tested in the manuscript.

L125 Why is the word greater in italics?

This formatting was used for emphasis but has been removed.

L154 I think it would be good to mention here what are the selection coefficients for low and high investments into RM.

A range of selection coefficients of the subpopulations was used across simulations to ensure robustness of our results. To clarify this, we now direct the reader to this information in the revised version of the manuscript (lines 140-141).

L168 "This is likely because RM systems are an imperfect barrier" I think this is an important result and I would highly appreciate having this explored in more detail in the model. So instead of having to say "likely" you could confidently say at what level of imperfection this eco-evolutionary dynamics drives both increased investment into  RM system as well as increased MGE acquisition.

We thank the reviewer for this insightful suggestion. We have now explored this with our mathematical modelling. In the original models, high RM investment led to 128-fold reduction in the probability of acquiring an unmethylated MGE. We have now run simulations with a range of values for the efficacy of the high investment RM system, ranging from 8 to 4096.

Interestingly, we find that the positive association between investment into RM and average count of MGEs remains consistent across this range of RM efficacy (although this relationship was negligible when the efficacy of the high investment RM system was low). We propose that this occurs because even when the high investment RM system causes a very high reduction in probability of acquiring an unmethylated MGE, it remains inactive against methylated MGEs that originate from other high RM investment cells. This shows that even when escape of an RM system is very rare, once it occurs MGEs can still spread freely within the sub-population that carries the same system.

For additional methods see lines 477-480, for results see lines 162-168 and Figure S1.3.

L193 This is really interesting! Any idea why?

We agree that these species-specific biases in the distributions of RM types are interesting! Previous analysis of how RM systems have shaped the evolution of *Salmonella enterica* found that Type III RM systems were ubiquitous, likely because they were acquired early on in the evolutionary history of the species (9). We also note that in the analyzed *Salmonella enterica* genomes, moderate counts of Type I, Type II, and Type IV RM systems were also observed, which could suggest selection for a diversity of RM strategies in this species rather than specifically selection for Type III systems. This could potentially be a response to carriage of counter defenses by phages/parasitic MGEs, although this is purely speculatory.

The high counts of Type IV RM systems in *Mycobacterium tuberculosis* are intriguing. Again, this species also has relatively high counts of Type II RM systems. We can speculate that in tandem, Type II and Type IV systems present a powerful barrier to phage/parasitic MGE infection. As Type II RM cleaves DNA when an unmethylated copy of the target motif is recognized, selection can drive carriage of broad spectrum methyltransferases by phages/MGEs to evade host defenses. The carriage of Type IV RM systems that cleave methylated DNA will then be selected for, to confer protection against these methylated phages/MGEs. Such an arms race would lead to a distribution of RM types like we see in *Mycobacterium tuberculosis*; high counts of RM systems that cleave unmethylated DNA, and high counts of systems that cleave only methylated DNA.

We have included some text in the main document where we consider both of these explanations (see lines 208-214).

L215 Could you highlight the selected species in the phylogenetic tree by some arrows and labels or so? Would be nice to see where they sit. However, in the document I received the PDF image quality was rather low so maybe it is somehow highlighted but I could not notice it.

The included phylogenetic trees display individual genomes of a single species, rather than the phylogenetic relationships between the species included in our dataset. A tree is included for all species for which a within-species analysis was conducted. We believe the image quality reduction was caused by the PDF generation. Submitted images were high-resolution.

L240 for the five species we conducted intraspecific bioinformatic analyses for,

This redundancy has been corrected.

L244 I find this claim problematic. While in the within species comparison one expects that various strains might co-occur within the same habitat, for between species comparison this is highly speculative. And your claim assumes that the probability of cooccurrence is the same within and between species, which I find rather unlikely (as for example the ecology of Enterococcus faecium, Streptococcus pyogenes are very different). As a result, the rate of ARG transfer is likely to drop with

We believe this comment by the reviewer may have been cut off, but we understand and appreciate the key point they are making; We acknowledge that previously our phrasing here was unclear and thank the reviewer for highlighting this oversight. Our intention was to state that per HGT opportunity, it is more probable that RM systems will function as a barrier between heterospecifics than conspecifics, as on average, it is more likely during an interspecific HGT opportunity that recipients will possess an RM system the donor does not. We have now clarified this and added additional context stating that in absolute terms, variation in encounter rates may lead RM to function more frequently as a barrier to intraspecific HGT (see lines 287-291 main text).

L312 Again, you used pathogenic bacteria only. While it the logic behind this decision is straightforward - pathogens are heghly exposed to antimicrobials, thus they evolve and transfer ARGs often - it needs to be clearly explained in the text.

We have now included acknowledgment of this in the text, and the caution about generalizing our findings to non-pathogens (see lines 355-361).

L316 This is kind of puzzling to me. Here you do mention that co-occurrence of different species does influence the probability of RM mediated HGT. Needs clarification with regards to my comment L244.

We acknowledge that this statement could have caused confusion with how the statement in lines 244 was originally presented. We hope that our changes to this sentence and additional sentences about the importance of encounter rates here have rectified this.

L355 Indeed!

We are glad the reviewer agrees with this statement.

L455 Keep past tense.

This has been corrected.

L456 Minus is not a verb as far as I know. Subtract is.

This has been corrected

L475 Have you considered potential episomes? Some MGEs may integrate into the chromosome.

In our analysis of whether significantly co-occurring ARGs and RM systems could be on/originated on the same MGE, we were indeed interested in both instances of colocalization on an MGE, and colocalization in the chromosome (due to originating on an MGE that has integrated). As in this analysis we are simply identifying instances where the ARG and RM system are within 100kb of each other, regardless of contig length or whether the contig is chromosomal or not, an integrated MGE carrying an RM system and ARG would still have been identified (evidenced by the identification of the conjugative prophage Φ1207.3 in the *S. pyogenes* genomes).

Table S2: What is meant by "ran on species"?

This was short for “random effect on species”. We have changed this to improve clarity.

Fig. 1 Both the main text and the figure caption mention an average count of MGEs. How is it calculated? Is it an average count throughout the simulations? Has equilibrium been reached in some of the simulations? I found this in the model description in SI but I think it would be good to briefly mention these aspects also in the main text or the figure label.

We have altered this legend (and Figure S1.1) so it is now clear that it is average count per individual once equilibrium has been reached.

Fig. 3 What exactly is meant by Predicted effect size?

We have added the following text to the figure legend: “The predicted effect is the change in count of ARGs per additional RM system, as predicted by a Bayesian Generalized Linear Mixed Model (GLMM).”.

Fig. S2 Where are the defense systems (S2.1) ARGs (S2.2) located? Are they all on MGEs? I would find it helpful to have these data presented in a main figure and described thoroughly in the main text. Also, better interconnection between the model and bioinformatic results would be beneficial.

In order to attain the necessary sample size required to investigate the relationship between count of RM systems and ARGs per genome in phylogenetically controlled models, we analyzed all genomes available from RefSeq for our chosen study species. Unfortunately, as a large proportion of these genomes are not complete/closed genomes, we were unable to reliably discern, in a high throughput manner, where ARGs or RM systems were located.

Fig. S6 A legend indicating what the two colors mean is missing. Also, the figure caption in SI is too brief. What are the plots showing?

Thank you for noticing this oversight. We have now added a legend to the figure and added more information to the accompanying text.

References

1.	Wong A. Epistasis and the Evolution of Antimicrobial Resistance. Front Microbiol [Internet]. 2017 Feb 17 [cited 2025 Nov 12];8. Available from: https://www.frontiersin.org/journals/microbiology/articles/10.3389/fmicb.2017.00246/full

2.	Pursey E, Dimitriu T, Paganelli FL, Westra ER, Houte SV. CRISPR-Cas is associated with fewer antibiotic resistance genes in bacterial pathogens. Philosophical Transactions of the Royal Society B. 2021;377:20202464.

3.	Stokar-Avihail A, Fedorenko T, Hör J, Garb J, Leavitt A, Millman A, et al. Discovery of phage determinants that confer sensitivity to bacterial immune systems. Cell. 2023 Apr 27;186(9):1863-1876.e16.

4.	Gao LA, Wilkinson ME, Strecker J, Makarova KS, Macrae RK, Koonin EV, et al. Prokaryotic innate immunity via pattern recognition of conserved viral proteins. Science. 2022 Aug 12;377(6607):eabm4096.

5.	Zhang T, Tamman H, Coppieters ’t Wallant K, Kurata T, LeRoux M, Srikant S, et al. Direct activation of a bacterial innate immune system by a viral capsid protein. Nature. 2022 Dec;612(7938):132–40.

6.	Huang J, Zhu K, Gao Y, Ye F, Li Z, Ge Y, et al. Molecular basis of bacterial DSR2 anti-phage defense and viral immune evasion. Nat Commun. 2024 May 10;15(1):3954.

7.	Tesson F, Huiting E, Wei L, Ren J, Johnson M, Planel R, et al. Exploring the diversity of anti-defense systems across prokaryotes, phages, and mobile genetic elements. bioRxiv. 2024 Aug 21;2024.08.21.608784.

8.	Koskella B. Resistance gained, resistance lost: An explanation for host–parasite coexistence. PLOS Biology. 2018 Sep 24;16(9):e3000013.

9.	Roer L, Hendriksen RS, Leekitcharoenphon P, Lukjancenko O, Kaas RS, Hasman H, et al. Is the Evolution of Salmonella enterica subsp. enterica Linked to Restriction-Modification Systems? mSystems. 2016;
