# Supplementary material for: Eco-evolutionary feedbacks drive the co-occurrence of restriction-modification systems and antimicrobial resistance genes in bacteria
Source: PLoS Biol. 2026 Jun 15;24(6):e3003842. doi: 10.1371/journal.pbio.3003842 (PMC13293514; doi:10.1371/journal.pbio.3003842)
Supplement: Attachment — Submitted filename: Responses_to_reviewers_resubmission_2.docx [file pbio.3003842.s021.docx]

------------------------------------------------------------------------
REVIEWERS' COMMENTS
------------------------------------------------------------------------


Reviewer #1:

I appreciate the authors' careful consideration in responding to my questions and comments, and their effort in thoroughly revising the manuscript.

We thank the reviewer for contributing their time to review the manuscript, and for providing useful feedback that has helped to strengthen this study.

In the first round of revisions Reviewer #1 suggested some changes to the description of the mathematical modelling methodology that we implemented, and which helped improve the clarity and flow of the section. We would like to highlight that we have now made some additional improvements to the Supplementary Information file’s mathematical modelling methods section. Previously, in this section, the full equation governing the change in sub-populations over time was presented as a single equation. Now, we present the equation’s major components in sequence, alongside a brief description of the presented terms, and only after all component equations are explained do we present the full equation. We believe this significantly improves the accessibility and clarity of this section. We additionally fixed some small typographical errors that were still present in the text of this section and in the equation itself.
------------------------------------------------------------------------

Reviewer #2:

After carefully reviewing the revised manuscript, I must say that while the dataset and overall framing are potentially impactful (and I appreciate the effort of the authors to address the reviewer feedback), several key assumptions remain insufficiently supported. Below I describe my main concerns in more detail.

REBASE cross-validation
While the idea of using the REBASE database of sequenced methylation patterns for confirmation of functionality of predicted RM systems can be useful, the data reported by the authors raise more concerns though.
First, methylation patterns differ among different types of RM systems. While type I and II systems do prevent self cleavage by methylating nearly all of their recognition sites, this is not true for type III and IV. Type III systems methylate only one strand of the DNA at their asymmetric recognition site. Therefore, many (about 50%) predicted sites remain unmethylated.

The reviewer’s concern is based on an incorrect understanding of how the proportion of methylated motifs of Type III systems is reported in the utilized dataset. It is true that Type III RM systems hemimethylate DNA, however, this is accounted for in the calculation; if one strand of a Type III RM recognition motif or its complement is methylated, this is recorded as a methylated site. Therefore, if all occurrences of a Type III RM motif are hemimethylated, this is reported as 100% of detected sites are methylated, not 50%.

Moreover, type IV systems work in an oposite way - restriction endonucleases cleave only methylated DNA, thus their recognition site must be unmethylated!
The pooling of all the RM system types into one category and expecting that their functionality is confirmed by methylation thus seems like a questinable assumption.

We agree with the referee that methylation patterns of genomes cannot be used to infer functionality of Type IV RM systems (because they don’t encode methyltransferases). We have therefore removed Type IV RM systems from the total count of annotated RM systems and reanalyzed our data. As expected, given that Type IV RM systems are rare (they make up only 7% of the annotated RM systems in our original dataset), these changes did not impact the conclusions of our validation.

Type I and II with only 50% sites methylated (selected cutoff in their analysis) would most likely lead to self cleavage, while methylation of 50% or more in type IV would also lead to self cleavage, pointing towards pseduogenised REases.

50% was chosen as a cutoff to conservatively exclude only motifs with dubiously low genome methylation coverage. If we observe the actual distribution of methylation coverage, for the vast majority of methylation patterns >95% of motif occurrences are modified (Figure 1, median percentage methylated is 97.7%). However, we agree with the reviewer that for RM Types I, II, and III, 50% of sites methylated is likely to result in self-cleavage, thus we have now increased the cutoff for inclusion in our analyses to 95% (although as can be seen in Figure 1, very few methylation patterns have coverage between 50% and 95%, so this change had limited impact on our analyses).

**Figure 1**. The distribution of the proportion of motifs modified, for all unique methylation patterns used in the cross-validation analysis.

Only in the case of type III systems this value would be a reasonable expectation.

As previously stated, the reviewer’s statement regarding how methylation coverage is reported for hemimethylated motifs is incorrect.

The data presented by the authors question their approach even more. Even when pooled together, one would expect that in clades with higher proportion of type III and IV systems, the ratio of detected RM systems to the count of modification patterns, should be higher (fewer modification patterns) than in clades with predominant type I and II (more modification patterns).

However, the data show the oposite. The largest RM:modification-pattern ratio is Neisseria gonorrhoeae (2.11 filtered), yet in their main Table 1, N. gonorrhoeae has 0 type IV systems. Conversely, species with very large type IV totals in the main dataset (e.g., S. aureus, S. enterica) show ratios < 1 in the REBASE subset (filtered 0.504 and 0.833, respectively), i.e., more methylation patterns than RM calls, not the inflation one would expect from abundant type IV systems.

We now utilize a Poisson GLM to test whether the total count of annotated Type I, II, and III RM systems differs significantly from the count of unique modification patterns per genome, for each species (Figure S3D). We find that for twelve out of fourteen species this difference is not statistically significant. For the two species the reviewer focuses on, this difference is significant.

For *N. gonorrhoeae* it is well known that the MTases and RM systems exhibit phase variation ^1,2^, meaning that at any one time, some MTases will be switched off (although they are still functional) and others will be switched on. The high degree of phase variation in this species means that observing more annotated systems than unique methylation patterns is expected.

For *S. aureus*, the decoupling observed between the count of annotated RM systems (Types I, II, and III only) and unique methylation patterns is intriguing and seemingly reflects the presence of methyltransferases in *S. aureus* genomes that are not being annotated as part of functional RM systems. As *S. aureus* carries many Type IV RM systems, and Type IV RM systems are known to co-occur and act synergistically with other non-RM methylation-dependent systems such as BREX ^3,4^, it is possible the higher than expected counts of methylation patterns in *S. aureus* are due to the presence of such non-RM methylation-dependent defenses, which would not be annotated by our pipeline.

We acknowledge that variation between counts of annotated RM systems and methylation signatures is expected when drawing on a large heterogeneous, public PacBio dataset, due to phase variation of methyltransferases, non-RM methylation-dependent defenses, as well as orphan methyltransferases and methylome detection biases. Our intention here was not to suggest we can explain all variation in methylomes through RM system annotation, but rather to offer independent evidence of RM functionality, as requested. Nevertheless, RM counts inferred by our pipeline closely matched the count of unique methylation patterns at the genome scale for twelve out of fourteen species.

Moreover, the major type IV outlier in the main dataset, Mycobacterium tuberculosis (they state >50% of all type IV systems are in M. tuberculosis; Table 1) is not included in the REBASE validation table even though many sequence isolates with annotated RM methylation patterns are present in REBASE. Thus, the validation does not test the regime where type IV inflation/decoupling from methylation should be most acute. Instead, Table S1 has E. coli, which is not included in the main bioinformatic analysis. Such selection is rather puzzling and does not imply a very rigorous approach.

We admit our explanation of how genomic and PacBio data were chosen and accessed for this validation was insufficiently clear in the original manuscript. We have now rectified this in the main text (lines 502-510).

Briefly, we utilized a publicly available file that details all GenBank submitted PacBio data that has been processed through PacBio’s RS_Modification_and_Motif_Analysis pipeline (downloadable from <https://ftp.ncbi.nlm.nih.gov/pub/supplementary_data/basemodification.csv>). From this file, we downloaded all genomes for which we could also obtain the respective motif_summary.csv and base_modification.csv files, for the fourteen species studied in the manuscript. Although in this file fourteen *M. tuberculosis* genomes have corresponding methylome data in the form of motif summary files, thirteen are archived and unavailable for public download. We exclude the final *M. tuberculosis* genome for which we can obtain methylome data as we cannot make any statistical inference from a single datapoint.

We decided to include *E. coli* in our validation pipeline as it enabled us to increase the size of our validation dataset considerably (241 additional genomes with corresponding methylome data), allowing more robust validation of the functionality of the RM systems our pipeline annotates. We are grateful that the reviewer has highlighted that we did not convey this reasoning in the submitted version of the methodology. This has now been rectified.

High variance in the RM-systems : methylation-patterns ratio also sharply limits what that test can legitimately validate. The filtered ratios range from 0.504 (S. aureus) to 2.110 (N. gonorrhoeae) (∼4-fold range), with similarly broad unfiltered ratios (0.236-1.25). That alone implies that even if the grand mean ratio is near 1 (the authors report 0.909 when filtering motifs by arbitrary >50% methylation) any inference about functionality is highly heterogeneous across taxa.

As previously stated, to empirically test the reviewers’ concerns, we have conducted a Poisson GLM analysis to validate whether the filtered counts of methylation patterns differ significantly from the count of annotated RM system per genome, and found that for only two species out of fourteen (the two highlighted by the reviewer, *N. gonorrhoeae* and *S. aureus*) there is a statistically significant difference (Figure S3D).

Such heterogeneity can be, apart from detection biases (insensitivity of PacBio SMRT sequencing to 5mC methylation), also caused by non-RM-related methylation, e.g., immunity related BREX/DISARM/MADS-like MTases and regulatory methylation signals (Dam, CcrM, Dcm, Phase-variable Type III Mod MTases etc…).

We thank the reviewer for the useful contributions about what drives this observed heterogeneity and agree with their reasoning. Although investigating the diversity of factors that influence variation in methylomes between species is an interesting question, it is not the focus of the present study that is being reviewed.

Taken together, I think one can make use of the curated REBASE PacBio database for confirmation of functionality of the predicted RM systems but with substantially more focus on the details mentioned above. The MTases there are classified based on the type, percentage of methylated sites, the recognition sequence and the type of modification.
From that data one can exclude for example Dam and Dcm MTases, which are well annotated there but cannot be included as a proxy for REase activity. Also, for such analysis I would appreciate if the authors could report (i) a per-genome scatterplot of RM count vs methylation-pattern count with a correlation (Spearman is fine), (ii) a comparison of the two measurement methods using Bland-Altman-style difference plot, and (iii) the analysis stratified by RM types), because type IV is expected to decouple from MTase-linked methylation signatures.

We thank the reviewer for these useful suggestions. We have now conducted the per-genome scatterplot with correlation analysis for Type I, II, and III RM systems combined (R = 0.67, P < 0.001; Figure S3B), as well as all RM types separately (Type I: R = 0.36; Type II: R = 0.58; Type III: R = 0.38; all P < 0.001; Figure S3C). As expected, no correlation was observed for Type IV systems (R = −0.12, P = 0.16), which provides a useful negative control for this analysis.

We also now include a Bland–Altman difference plot, demonstrating strong agreement between these measures, with 95.04% of genomes falling within the limits of agreement (LOA) (Figure S3A), with the majority of genomes that fall outside the LOA belonging to *N. gonorrhoeae*, as expected.

We have considered the suggestion to exclude methylation patterns from the analysis that belong to the Dam and Dcm methylases. However, in the obtained motif summary data, we cannot know if motifs are methylated due to a methyltransferase such as Dam/Dcm, or due to an RM system that also recognizes the same motif. For example, there are 2400 entries on REBASE for nucleases belonging to Type II RM systems that cleave the same motif that Dam methylates. Exclusion of methylome data for these well-known methyltransferases could thus introduce biases by excluding methylation patterns belonging to functional RM systems.

Another observation which is puzzling to me, is that in the REBASE cross-validation, Table S1 reveals that several species are supported by very small numbers of methylome-profiled genomes (e.g., 4 S. pyogenes, 6 E. faecium). The manuscript states that the authors used 'all publicly available genomes' for which 'complete motif modification profiles' could be obtained from REBASE (584 genomes total), but it does not specify the practical filters that lead to such limited per-species coverage (e.g., requirement for closed/RefSeq-complete assemblies, accession matching, or specific SMRT outputs), nor provide an accession list and download date to allow readers to verify completeness and representativeness. A very brief search showed that for example in S. pyogenes, the REBASE PacBio database contains 55 complete genomes with annotated methylomes, with variable methylation patterns.
Because these small Ns can affect the inferred RM:motif ratios for individual taxa, the authors should explicitly document the REBASE query/criteria, provide a list of included accessions, and discuss how limited and uneven methylome availability impacts the strength of this validation.

We have clarified in response to a previous comment that we miscommunicated in the methodology exactly which genomes and methylomes were used in the cross-validation analysis. However, in the reviewer’s initial comment that suggested that we attempt to validate the functionality of annotated RM systems, it was suggested that this be done for “a small subsample” of genomes. We believe 584 bacterial genomes spanning fourteen species clearly exceeds the reviewer’s own suggestion for sample size.

Species selection for GLMMs
This detailed examination of the reported measures brought me back to their initialy reported findings. The selection of species for the within-species phylogeny-controlled GLMMs (Fig. 3) currently appears insufficiently justified and potentially prone to selection bias. The authors state that they excluded several taxa because they lacked "heterogeneity in RM system counts," yet their own Table S3 indicates substantial within-species diversity of RM system content (high Shannon indices) in multiple excluded species, which makes it unclear why these taxa were deemed uninformative.

Here we quote the methods section from the submitted manuscript that describes species selection for GLMMs:

“These species were chosen as they are taxonomically diverse and span the full range of both ARG and RM system counts per genome seen in the full dataset. We also excluded species that lacked heterogeneity in their RM system counts (for example, *Shigella sonnei* and *Streptococcus flexneri* genomes were highly uniform in their RM system content and thus statistical power would be limited).”

Species selection was done to ensure taxonomic diversity (these five species represent five different families and span three phyla) as well as RM content diversity (that is, picking species with a range of mean counts of RM systems, *not* picking species with high within-species RM diversity). We believe it is clear in the second quoted sentence that we chose to **exclude** two species that lacked variation in RM system content across genomes, not that we chose to **include** all species for which RM content of genomes varied above a certain threshold.

Because Shannon diversity of RM identities need not map onto variance in total RM counts per genome, the appropriate way to support the exclusion criterion would be to report, for all 14 species, the empirical dispersion of RM counts per genome (e.g. a violin/boxplot) and ideally estimate species-specific RM slopes in a hierarchical (partial-pooling) model rather than presenting results for a pre-selected subset.

As previously stated, species were not selected based solely on the criterion of within-species RM diversity.

We do not follow what the proposed advantages are of pooling all genomes and nesting all within-species phylogenetic trees within the same covariance structure to attempt to construct a single highly complex model. Such a model would suffer issues relating to differences in phylogenetic depth between species and could bias more extreme within-species slopes towards the global slope. Conducting analyses within-species is perfectly valid, more interpretable, allows estimates to be specific to the respective species, and avoids many issues relating to fitting such highly complex models.

Without this, it remains possible that some excluded species would show null or even opposite within-species associations, and the scope of the "within-species" claim should be restricted to solely the five species that were actually analyzed.

As stated previously, our findings may not generalize beyond the species analysed (lines 356-358). Moreover, a partially pooled model would face the same limitation, as inference would still be restricted to the included taxa.

Varying RM efficacy
I appreciate that the authors performed a model robustness analysis by varying RM efficacy (i.e., the assumed strength of RM-mediated protection during HGT) and showed that the qualitative association of interest is stable across a range of efficacies (the new supplemental analysis/figure). This also directly addresses my concern about unmodelled anti-restriction/anti-RM mechanisms: while they do not explicitly model particular anti-RM genes or pathways, sweeping the efficacy parameter effectively captures the net impact of partial RM evasion or inhibition and tests whether the conclusions depend on near-perfect restriction.

We are pleased the reviewer has found our modelling of varying RM efficacies useful for interpreting how robust our modelling findings are, and for indirectly modelling the effect of anti-restriction.

Trait depth inference
The authors' defense of trait depth is based on the assumption that (i) extensive HGT sufficient to make a trait appear "deep" (>0.9 clade positivity) would also tend to disseminate the trait beyond that clade, and (ii) recombination should blur RM and ARG signals in broadly comparable ways.
Both assumptions are questionable given empirical evidence that gene flow can be highly structured, with exchange occurring far more frequently within biologically coherent populations than between them, producing sharp gene-flow-defined clusters even among close relatives. ([https://eur03.safelinks.protection.outlook.com/?url=https%3A%2F%2Fdoi.org%2F10.1016%2Fj.cell.2019.06.033&data=05%7C02%7Cjw1235%40exeter.ac.uk%7C4c607c9aa6f34596f39108de5d905c33%7C912a5d77fb984eeeaf321334d8f04a53%7C0%7C0%7C639051071805652822%7CUnknown%7CTWFpbGZsb3d8eyJFbXB0eU1hcGkiOnRydWUsIlYiOiIwLjAuMDAwMCIsIlAiOiJXaW4zMiIsIkFOIjoiTWFpbCIsIldUIjoyfQ%3D%3D%7C0%7C%7C%7C&sdata=VcvHGb9uzW9GPzvzZVcXFykr7l6vzCoj0cyPOZtpgMY%3D&reserved=0](https://eur03.safelinks.protection.outlook.com/?url=https%3A%2F%2Fdoi.org%2F10.1016%2Fj.cell.2019.06.033&data=05|02|jw1235@exeter.ac.uk|4c607c9aa6f34596f39108de5d905c33|912a5d77fb984eeeaf321334d8f04a53|0|0|639051071805652822|Unknown|TWFpbGZsb3d8eyJFbXB0eU1hcGkiOnRydWUsIlYiOiIwLjAuMDAwMCIsIlAiOiJXaW4zMiIsIkFOIjoiTWFpbCIsIldUIjoyfQ%3D%3D|0|||&sdata=VcvHGb9uzW9GPzvzZVcXFykr7l6vzCoj0cyPOZtpgMY%3D&reserved=0))
In such a regime, a horizontally acquired trait can plausibly sweep to near-fixation within a population/clade (meeting the ≥0.9 criterion) while remaining rare outside it, so deep trait depth does not necessarily indicate old/vertical inheritance.
Moreover, phage-defense repertoires can turn over extremely rapidly via mobile genetic elements, thus effectively decoupling defense presence from deeper phylogenetic structure and making within-clade saturation compatible with recent gain and fast turnover. ([https://eur03.safelinks.protection.outlook.com/?url=https%3A%2F%2Fdoi.org%2F10.1126%2Fscience.abb1083&data=05%7C02%7Cjw1235%40exeter.ac.uk%7C4c607c9aa6f34596f39108de5d905c33%7C912a5d77fb984eeeaf321334d8f04a53%7C0%7C0%7C639051071805677372%7CUnknown%7CTWFpbGZsb3d8eyJFbXB0eU1hcGkiOnRydWUsIlYiOiIwLjAuMDAwMCIsIlAiOiJXaW4zMiIsIkFOIjoiTWFpbCIsIldUIjoyfQ%3D%3D%7C0%7C%7C%7C&sdata=r7Az1hpj%2FOLFLjCpAl4dYRblwZBGOgUN2bYk8wG8uuM%3D&reserved=0](https://eur03.safelinks.protection.outlook.com/?url=https%3A%2F%2Fdoi.org%2F10.1126%2Fscience.abb1083&data=05|02|jw1235@exeter.ac.uk|4c607c9aa6f34596f39108de5d905c33|912a5d77fb984eeeaf321334d8f04a53|0|0|639051071805677372|Unknown|TWFpbGZsb3d8eyJFbXB0eU1hcGkiOnRydWUsIlYiOiIwLjAuMDAwMCIsIlAiOiJXaW4zMiIsIkFOIjoiTWFpbCIsIldUIjoyfQ%3D%3D|0|||&sdata=r7Az1hpj%2FOLFLjCpAl4dYRblwZBGOgUN2bYk8wG8uuM%3D&reserved=0)).
Together, these observations imply that trait depth may primarily reflect population structure and within-population sweeps rather than acquisition timing, and that differential mobility/turnover of RM-linked defenses versus ARGs could systematically bias the intended "recent vs deep" comparisons.

The consenTRAIT metric we use to calculate trait depth is a widely used and accepted methodology for estimating the evolutionary time since a trait was acquired in microorganisms (see all citations of the landmark paper by Martiny *et al* 2013 ^5^). We agree that under the specific conditions the reviewer describes namely, rates of HGT high enough to cause a mobile gene to reach fixation, but populations so structured the gene is not mobilized outside of the population, it would overestimate the true time since acquisition. However, given that here we are still considering within-species HGT, and HGT between even different but closely related species remains high ^6^, it is improbable that such high within-species structuring of HGT is generalizable across species. Furthermore, in the reviewer’s own example, the mobile gene which has reached fixation must have originally been transferred **into** the structured population, casting doubt on the suggested inability of the gene to be transferred **out** of the structured population.

Ultimately, we accept that, like all bioinformatic tools and metrics, trait depth represents an imperfect but useful approximation, and the reviewer’s conclusion that it is not fit for purpose is clearly not a view widely reflected in the literature. Furthermore, we would like to restate that in our study we use trait depth to attempt to validate the robustness of our analyses, specifically when considering just high trait depth RM systems and low trait depth ARGs. The results are presented in an SI figure (Figure S6), show a null result, and are not central to the main conclusions of the paper.

Overall assessment
Overall, while the revised manuscript addresses some technical points, the revised analyses and responses introduce (and in several places leave unresolved) substantial concerns about the biological interpretation and the validity of key inferential steps. Most notably the use of methylome patterns as support for RM functionality, the ad hoc species selection for within-species effect estimation, and the reliance on trait depth as a proxy for temporal depth under structured gene flow and rapid defense turnover.
At the end of the day, these discrepancies undermine confidence in the central claims, and I therefore cannot recommend the article for publication in its current form. While I still find the idea of using eco-evolutionary dynamics and mathematical modelling to interpret patterns in HGT compelling, resolving the resulting doubts would require extensive additional analyses and reframing beyond what I would consider feasible as a minor or moderate revision. For this reason, I refrain from commenting on the authors' other responses.

We appreciate the time and effort spent by the referee to scrutinize our manuscript. Their feedback has helped us to further improve the manuscript, for which we are grateful.

References

1.	Seib, K. L., Jen, F. E.-C., Scott, A. L., Tan, A. & Jennings, M. P. Phase variation of DNA methyltransferases and the regulation of virulence and immune evasion in the pathogenic Neisseria. *Pathog Dis* **75**, ftx080 (2017).

2.	Sánchez-Busó, L., Golparian, D., Parkhill, J., Unemo, M. & Harris, S. R. Genetic variation regulates the activation and specificity of Restriction-Modification systems in Neisseria gonorrhoeae. *Sci Rep* **9**, 14685 (2019).

3.	Picton, D. M. *et al.* The phage defence island of a multidrug resistant plasmid uses both BREX and type IV restriction for complementary protection from viruses. *Nucleic Acids Research* https://doi.org/10.1093/nar/gkab906 (2021) doi:10.1093/nar/gkab906.

4.	Makarova, K. S., Wolf, Y. I. & Koonin, E. V. Comparative genomics of defense systems in archaea and bacteria. *Nucleic Acids Res* **41**, 4360–4377 (2013).

5.	Martiny, A. C., Treseder, K. & Pusch, G. Phylogenetic conservatism of functional traits in microorganisms. *ISME J* **7**, 830–838 (2013).

6.	Soucy, S. M., Huang, J. & Gogarten, J. P. Horizontal gene transfer: building the web of life. *Nat Rev Genet* **16**, 472–482 (2015).
