# Supplementary material for: Eco-evolutionary feedbacks drive the co-occurrence of restriction-modification systems and antimicrobial resistance genes in bacteria
Source: PLoS Biol. 2026 Jun 15;24(6):e3003842. doi: 10.1371/journal.pbio.3003842 (PMC13293514; doi:10.1371/journal.pbio.3003842)
Supplement: Peer review history

# Editor report: Decision Letter 0

**DOI:** 10.1371/journal.pbio.3003842.r001

**Authors:** Melissa Vazquez Hernandez

6 May 2025

Dear Joseph, dear Edze,

Thank you for submitting your manuscript entitled "Eco-evolutionary feedbacks drive the co-occurrence of restriction-modification systems and antimicrobial resistance genes" for consideration as a Research Article by PLOS Biology.

Your manuscript has now been evaluated by the PLOS Biology editorial staff, as well as by an academic editor with relevant expertise, and I am writing to let you know that we would like to send your submission out for external peer review.

However, before we can send your manuscript to reviewers, we need you to complete your submission by providing the metadata that is required for full assessment. To this end, please login to Editorial Manager where you will find the paper in the 'Submissions Needing Revisions' folder on your homepage. Please click 'Revise Submission' from the Action Links and complete all additional questions in the submission questionnaire.

Once your full submission is complete, your paper will undergo a series of checks in preparation for peer review. After your manuscript has passed the checks it will be sent out for review. To provide the metadata for your submission, please Login to Editorial Manager (https://www.editorialmanager.com/pbiology) within two working days, i.e. by May 08 2025 11:59PM.

If your manuscript has been previously peer-reviewed at another journal, PLOS Biology is willing to work with those reviews in order to avoid re-starting the process. Submission of the previous reviews is entirely optional and our ability to use them effectively will depend on the willingness of the previous journal to confirm the content of the reports and share the reviewer identities. Please note that we reserve the right to invite additional reviewers if we consider that additional/independent reviewers are needed, although we aim to avoid this as far as possible. In our experience, working with previous reviews does save time.

If you would like us to consider previous reviewer reports, please edit your cover letter to let us know and include the name of the journal where the work was previously considered and the manuscript ID it was given. In addition, please upload a response to the reviews as a 'Prior Peer Review' file type, which should include the reports in full and a point-by-point reply detailing how you have or plan to address the reviewers' concerns.

During the process of completing your manuscript submission, you will be invited to opt-in to posting your pre-review manuscript as a bioRxiv preprint. Visit http://journals.plos.org/plosbiology/s/preprints for full details. If you consent to posting your current manuscript as a preprint, please upload a single Preprint PDF.

Feel free to email us at plosbiology@plos.org if you have any queries relating to your submission.

Best wishes,

Melissa

Melissa Vazquez Hernandez, Ph.D.

Associate Editor

PLOS Biology

mvazquezhernandez@plos.org

# Peer review: Decision Letter 1

**DOI:** 10.1371/journal.pbio.3003842.r002

**Authors:** Melissa Vazquez Hernandez, Melissa Vazquez Hernandez

17 Jul 2025

Dear Joe,

Thank you for your patience while your manuscript "Eco-evolutionary feedbacks drive the co-occurrence of restriction-modification systems and antimicrobial resistance genes" was peer-reviewed at PLOS Biology. It has now been evaluated by the PLOS Biology editors, an Academic Editor with relevant expertise, and by two independent reviewers. I would like to sincerely apologize for the long time the peer-review process took.

In light of the reviews, which you will find at the end of this email, we would like to invite you to revise the work to thoroughly address the reviewers' reports. As you will see below, the reviewers think the topic is important and the results potentially interesting, yet some concerns have raised during revision. Reviewer 1 mentions that the study requires clarifications and corrections, specifically regarding the model, suggesting additional simulations. Reviewer 2 thinks the study requires certain aspects to be further discussed in depth to support the conclusions. For example, the reviewer suggests to use RNAseq data to distinguish between functional and non-functional RM systems, to statistically analyse the influence of other defense systems, and additional model simulations including MGE counter-defenses. We agree with all reviewer concerns and would require some additional experimental revisions to address them, as we consider that this would strengthen the work.

IMPORTANT: after discussion with the Academic Editor, we think 1) you should respond to all points; and 2) address in some way R2's point 1 regarding assumption of RM functionality, as this is central to the validity of the study. It could be RNAseq as suggested by the reviewer, or some other suitable approach accessible to the you.

Given the extent of revision needed, we cannot make a decision about publication until we have seen the revised manuscript and your response to the reviewers' comments. Your revised manuscript is likely to be sent for further evaluation by all or a subset of the reviewers.

In addition to these revisions, you will need to complete some formatting changes, which you will receive in a follow up email. A member of our team will be in touch with a set of requests shortly.

We expect to receive your revised manuscript within 3 months. Please email us (plosbiology@plos.org) if you have any questions or concerns, or would like to request an extension.

At this stage, your manuscript remains formally under active consideration at our journal; please notify us by email if you do not intend to submit a revision so that we may withdraw it.

**IMPORTANT - SUBMITTING YOUR REVISION**

Your revisions should address the specific points made by each reviewer. Please submit the following files along with your revised manuscript:

1. A 'Response to Reviewers' file - this should detail your responses to the editorial requests, present a point-by-point response to all of the reviewers' comments, and indicate the changes made to the manuscript.

*NOTE: In your point-by-point response to the reviewers, please provide the full context of each review. Do not selectively quote paragraphs or sentences to reply to. The entire set of reviewer comments should be present in full and each specific point should be responded to individually, point by point.

You should also cite any additional relevant literature that has been published since the original submission and mention any additional citations in your response.

2. In addition to a clean copy of the manuscript, please also upload a 'track-changes' version of your manuscript that specifies the edits made. This should be uploaded as a "Revised Article with Changes Highlighted" file type.

*Re-submission Checklist*

When you are ready to resubmit your revised manuscript, please refer to this re-submission checklist: https://plos.io/Biology_Checklist

To submit a revised version of your manuscript, please go to https://www.editorialmanager.com/pbiology/ and log in as an Author. Click the link labelled 'Submissions Needing Revision' where you will find your submission record.

Please make sure to read the following important policies and guidelines while preparing your revision:

*Published Peer Review*

Please note while forming your response, if your article is accepted, you may have the opportunity to make the peer review history publicly available. The record will include editor decision letters (with reviews) and your responses to reviewer comments. If eligible, we will contact you to opt in or out. Please see here for more details:

https://blogs.plos.org/plos/2019/05/plos-journals-now-open-for-published-peer-review/

*PLOS Data Policy*

Please note that as a condition of publication PLOS' data policy (http://journals.plos.org/plosbiology/s/data-availability) requires that you make available all data used to draw the conclusions arrived at in your manuscript. If you have not already done so, you must include any data used in your manuscript either in appropriate repositories, within the body of the manuscript, or as supporting information (N.B. this includes any numerical values that were used to generate graphs, histograms etc.). For an example see here: http://www.plosbiology.org/article/info%3Adoi%2F10.1371%2Fjournal.pbio.1001908#s5

*Blot and Gel Data Policy*

We require the original, uncropped and minimally adjusted images supporting all blot and gel results reported in an article's figures or Supporting Information files. We will require these files before a manuscript can be accepted so please prepare them now, if you have not already uploaded them. Please carefully read our guidelines for how to prepare and upload this data: https://journals.plos.org/plosbiology/s/figures#loc-blot-and-gel-reporting-requirements

*Protocols deposition*

To enhance the reproducibility of your results, we recommend that if applicable you deposit your laboratory protocols in protocols.io, where a protocol can be assigned its own identifier (DOI) such that it can be cited independently in the future. Additionally, PLOS ONE offers an option for publishing peer-reviewed Lab Protocol articles, which describe protocols hosted on protocols.io. Read more information on sharing protocols at https://plos.org/protocols?utm_medium=editorial-email&utm_source=authorletters&utm_campaign=protocols

Thank you again for your submission to our journal. We hope that our editorial process has been constructive thus far, and we welcome your feedback at any time. Please don't hesitate to contact us if you have any questions or comments.

Sincerely,

Melissa

Melissa Vazquez Hernandez, Ph.D.

Associate Editor

PLOS Biology

mvazquezhernandez@plos.org

------------------------------------

REVIEWERS' COMMENTS

------------------------------------

Reviewer #1:

Authors test the hypothesis that stronger selection for restriction-modification (RM) systems at higher exposure to costly MGEs results in the observed higher number of MGEs in bacteria with more RM systems. Their findings come from the model simulations, on one side, and from the bioinformatics analysis of >40,000 bacterial genomes, on the other. The topic and the goals of this study are interesting and important, but at this point there are many things in the presented manuscript that need to be clarified, justified or, potentially, corrected.

***************

MODEL

(I kindly ask Authors to comment on what I didn't understand correctly, if there is anything, and to also clarify the same in the manuscript.) According to the described model, I understood that a population of bacteria consists of subpopulations characterized by all possible combinations of the following two characteristics:

i) RM system presence & influence (n denotes the scenario), which can be: (1) RM system absence with no (0) effect on the host cells reproduction rate and the likelihood of acquiring an MGE, (2) presence of a hypothetical RM system 1 with low inhibitory effect on the host cells reproduction rate and the likelihood of acquiring an MGE, (3) presence of a hypothetical RM system 2 (with a different target sequence) with high inhibitory effect on the host cells reproduction rate and the likelihood of acquiring an MGE;

ii) MGE presence (i denotes the number of different MGEs present in a cell, not the copy number of the same MGE), where the number of MGEs in a cell can be 0, 1, 2, 3, 4 or 5.

In a compartmental (ODE) model, cell number S_n,i of a given sub-population:

1) increases by division of those cells (where the reproduction rate is negatively affected by RM system and MGEs presence);

2) increases by MGE loss in Sn,i+1 cells (with the same RM system; one MGE is lost completely, not transferred);

3) increases by MGE gain in Sn,i-1 cells (where methylated and unmethylated MGEs are gained with different rates);

4) decreases by cell death (complete elimination from the population);

5) decreases by MGE loss (one MGE is lost completely);

6) decreases by MGE gain (where methylated and unmethylated MGEs are gained with different rates).

I suggest adding a similar conceptual description of the modelled population in the main text, or providing in another way (maybe with and illustration) this information that is needed for the interpretation and understanding of the results.

It seems that the terms corresponding to rates under 3) and 5) are missing from the model (eq. in Supplementary document, lines 40-42)?

(Supplementary document S2.1, 1st paragraph) I assume it should read 'i' MGEs and having RM system 'n'.

Why did you choose this particular dependence of the reproduction rate on the number and costs of MGEs? The strong nonlinear decay of this rate with the number of MGEs needs additional justification.

(Main text, L369) Here authors state that cells in the model can acquire 5 "identical" MGEs and this needs to be clarified. Are different MGEs/ARGs considered, which can also replicate in the host cell, so for another cell to acquire an MGE, it is not needed that the first cell looses its MGE? Or are the copy numbers of the same MGE considered? In that case, dynamics of the MGE number should probably also be described by the model.

(Main text, L160-166) Please explain more directly how you define and vary the population level RM diversity in the model. By setting initial conditions so that there are different numbers of cells with 0, low and high RM investment and examining the state of the system at the end of a simulation?

Does the finding that "bacteria with high RM investment also had a higher mean count of MGEs" depends on which RM systems are most prevalent in the low diversity scenario? E. g. if high RM investment cells comprise the majority of the population?

I wonder if simulating the scenarios of no diversity (all cells in the population are of the same RM investment level) would be helpful in interpreting the results. Also, I would like to know what are the relative sizes of the sub-populations at the end of the simulations - e. g. is the number of MGEs that end up in no-RM cells low because these cells loose in the competition, or because they win so their number is actually large.

(Main text, L167-169) How do the "simulations suggest high exposure to costly MGEs can drive an increased investment into RM-mediated genome defence" when cells in the model cannot change their RM status (acquire new RM systems) in response to the varying MGE costs?

Generally, "investment in RM systems" can be misleading and it could be replaced by e. g. "the cost of maintaining an active RM system". Specifically, one may conclude that cells choose if they are going to keep an RM system or not and that would need to be further discussed.

***************

BIOINFORMATICS

(Main text, L234-236) Does this hypothesis assume the equal probability for all species to come into contact with each other, i. e. live together in the same environment?

(Main text, L313-316) If the species selected for the analysis do not share the same environment, and are therefore typically not expected to share any MGEs via HGT, as authors mention, such a dataset seems inappropriate for a statistical analysis aiming to clarify the relationship between acquired RM systems and ARGs between species ("understanding the role RM plays in regulating HGT of ARGs").

Please add the information from Supplementary document L72-75 ("The trait depth filtered dataset"), on the dataset used in the analysis, to the Methods section in the main text.

------------------------------------

Reviewer #2:

This manuscript presents a synthesis of mathematical modelling and comparative genomics to investigate the eco-evolutionary dynamics underlying the co-occurrence of restriction-modification (RM) systems and antimicrobial resistance genes (ARGs).

The authors show that population-level RM diversity critically modulates the direction of association between RM systems and horizontally acquired ARGs, offering a robust explanation for previously paradoxical genomic patterns. Their large-scale analysis of >40,000 bacterial pathogens' genomes lends empirical support to the model predictions, distinguishing between-species negative correlations from within-species positive associations. The study rules out genetic linkage as a major explanatory factor and instead highlights how imperfect barriers and shared methylation facilitate ARG dissemination among RM-compatible strains.

Overall, the work provides insight into the eco-evolutionary forces shaping genome defense and resistance, with important implications for AMR spread.

General assessment

The general results of the study very interesting, especially because it is taking into consideration eco-evolutionary interactions and infers how they influence genome architecture.

However, some major aspects need to be discussed and addressed in greater depth in order to fully justify the inferred conclusions.

Here are some conceptual aspects (major points):

1. Assumption of RM Functionality: The study assumes that all annotated RM systems are functional without any direct validation. Given that many RM genes are cryptic or degraded, this could overestimate their barrier effect. While bioinformatic annotations alone cannot distinguish functional RM systems from degraded or non-functional remnants, RNAseq data on a small subsample could give at least a clue about what fraction of RM systems are expressed and what are non-expressed pseudogenes.

2. Trait Depth Inference: The use of trait depth as a proxy for acquisition timing lacks resolution and may not reliably disentangle the temporal order of RM and ARG acquisition, especially in recombining lineages. Trait depth is inferred from phylogenetic distribution and assumes vertical inheritance, but in recombining lineages, horizontal gene transfer can blur this signal. If either RM systems or ARGs have been frequently gained or lost through recombination, their phylogenetic placement may not reflect true acquisition timing, leading to inaccurate inferences about which trait arose first. A recent study (https://doi.org/10.1073/pnas.2316302121) showed that in many of the analyzed species the rate of homologous recombination is exceptionally high, indicating that the assumption of solely vertical inheritance is an oversimplification.

3. Limited Consideration of Other Defense Systems: The analysis focuses exclusively on RM systems, ignoring potentially confounding or interacting effects from co-occurring systems like CRISPR-Cas or abortive infection systems. Since other systems have also been annotated (Fig. S2), a statistical analysis of their influence on the probability of ARGs could also be included and would vastly improve the impact of the paper (even if no significant effect is found).

4. Simplified Modeling Framework: The mathematical model does not include key MGE counter-defenses (e.g., anti-restriction systems), which limits its capacity to fully capture real-world eco-evolutionary dynamics. While the authors speculate about their potential impact, some qualitative example simulations of a model, which includes them would be highly beneficial. Also, showing prevalence of anti-restriction systems and RM systems would imply something about their evolutionary importance.

5. Bacterial pathogens only: Pathogenic life style is ecologically very distinct from other non-pathogenic life styles and often shows specific genomic signatures (pathogenicity islands carrying virulence genes, genome reduction, elevated rates of HGT, proliferation of IS elements, pseudogene accumulation, altered GC-content… ). I understand that spread of ARGs in bacterial pathogens is of high medical importance, however in my opinion it deserves explanation in the introduction (not just Materials and Methods). Furthermore, the results should be interpreted more carefully because the findings may not hold for non-pathogenic bacteria.

And some minor comments:

L109 I would strongly suggest using other wording of the sentence and avoid putting the word imperfect in the brackets. It blurs the intention of the authors.

L119 I agree that RM systems can influence homologous recombination rates in Neisseria meningitidis. However, the relevance of the statement here seems to me rather questionable. The paper focusses on HGT of MGEs and suddenly introduces homologous recombination. Is it a special case of homologous recombination within MGEs? If so, please explain. If not, I would leave the sentence out.

L125 Why is the word greater in italics?

L154 I think it would be good to mention here what are the selection coefficients for low and high investments into RM.

L168 "This is likely because RM systems are an imperfect barrier" I think this is an important result and I would highly appreciate having this explored in more detail in the model. So instead of having to say "likely" you could confidently say at what level of imperfection this eco-evolutionary dynamics drives both increased investment into RM system as well as increased MGE acquisition.

L193 This is really interesting! Any idea why?

L215 Could you highlight the selected species in the phylogenetic tree by some arrows and labels or so? Would be nice to see where they sit. However, in the document I received the PDF image quality was rather low so maybe it is somehow highlighted but I could not notice it.

L240 for the five species we conducted intraspecific bioinformatic analyses for,

L244 I find this claim problematic. While in the within species comparison one expects that various strains might co-occur within the same habitat, for between species comparison this is highly speculative. And your claim assumes that the probability of cooccurrence is the same within and between species, which I find rather unlikely (as for example the ecology of Enterococcus faecium, Streptococcus pyogenes are very different). As a result, the rate of ARG transfer is likely to drop with

L312 Again, you used pathogenic bacteria only. While it the logic behind this decision is straightforward - pathogens are heghly exposed to antimicrobials, thus they evolve and transfer ARGs often - it needs to be clearly explained in the text.

L316 This is kind of puzzling to me. Here you do mention that co-occurrence of different species does influence the probability of RM mediated HGT. Needs clarification with regards to my comment L244.

L355 Indeed!

L455 Keep past tense.

L456 Minus is not a verb as far as I know. Subtract is.

L475 Have you considered potential episomes? Some MGEs may integrate into the chromosome.

Table S2: What is meant by "ran on species"?

Fig. 1 Both the main text and the figure caption mention an average count of MGEs. How is it calculated? Is it an average count throughout the simulations? Has equilibrium been reached in some of the simulations? I found this in the model description in SI but I think it would be good to briefly mention these aspects also in the main text or the figure label.

Fig. 3 What exactly is meant by Predicted effect size?

Fig. S2 Where are the defense systems (S2.1) ARGs (S2.2) located? Are they all on MGEs? I would find it helpful to have these data presented in a main figure and described thoroughly in the main text. Also, better interconnection between the model and bioinformatic results would be beneficial.

Fig. S6 A legend indicating what the two colors mean is missing. Also, the figure caption in SI is too brief. What are the plots showing?

# Author comment: Author response to Decision Letter 2

**DOI:** 10.1371/journal.pbio.3003842.r003

2 Dec 2025

Attachment Submitted filename: Responses_to_reviewers.docx

# Peer review: Decision Letter 2

**DOI:** 10.1371/journal.pbio.3003842.r004

**Authors:** Melissa Vazquez Hernandez, Melissa Vazquez Hernandez, Melissa Vazquez Hernandez

27 Jan 2026

Dear Joe,

Thank you for your patience while the revision of your manuscript "Eco-evolutionary feedbacks drive the co-occurrence of restriction-modification systems and antimicrobial resistance genes" was re-reviewed at PLOS Biology. Your study has now been assessed and discussed by the PLOS Biology editors, an Academic Editor with relevant expertise and the original reviewers. Please accept my apologies for the delay in reaching a decision.

We have now received and carefully considered the reviewers’ final comments on your revision. Reviewer #1 is satisfied with the responses and revisions and recommends acceptance. Reviewer #2, however, raises several major concerns that they consider to be unresolved and that, in their view, limit confidence in the central conclusions of the study.

In brief, Reviewer #2 questions whether the current REBASE-based analyses provide sufficiently robust support for RM system functionality, given differences among RM types and heterogeneous, limited methylome sampling; whether the selection of species used for the within-species analyses is adequately justified, and whether the resulting conclusions can be generalized; and whether trait depth can reliably be interpreted as a proxy for acquisition timing under structured gene flow and rapid turnover of defense systems.

I discussed these final comments with the Academic Editor. While we recognize that one reviewer is supportive, the Academic Editor agreed that the outstanding concerns raised by Reviewer #2 are central to the biological interpretation and validity of key inferential steps in the manuscript. Addressing them convincingly would likely require substantial additional analyses and potentially reframing of core aspects of the study, with an uncertain outcome regarding whether the main conclusions would remain supported.

Based on these considerations, I regret that we will not be able to invite a further revision of the manuscript. Successfully resolving the remaining issues would entail a significant amount of work beyond what could reasonably be expected of another revision round.

Because producing the required additional analyses and supporting evidence would require substantial effort with an uncertain outcome, we cannot in good faith invite revision of your study. However, given our and the reviewers’ interest in the topic, if further work were to allow you to comprehensively address the major concerns outlined above, we would be willing to consider an extensively revised manuscript as a new submission. The article would receive a new manuscript number and submission date and would be evaluated in view of any related work published in the interim, but we would make every effort to engage the same Academic Editor, yet two new reviewers, if no novelty concerns arise.

We appreciate that the scale of the requested additional work is significant and understand that you may prefer to pursue faster publication of this work elsewhere. At this point, your manuscript is no longer under active consideration at PLOS Biology. We might be able to facilitate a faster consideration at another PLOS journal; if this is of interest to you, please let me know.

Please feel free to contact me if you would like to discuss next steps for your manuscript. If you do make the choice to revise and re-submit to PLOS Biology, please provide a point-by-point response to the reviewers' comments when re-submitting. You should include this as a separate file, labelled 'Response to Reviewers'. Please also provide the tracking number of this manuscript [PBIOLOGY-D-25-01423R2] in the 'previous interaction' section of the online submission form and in your cover letter.

I am sorry that we cannot be more positive on this occasion. I thank you for having considered PLOS Biology for publication and hope that you will find the reviewers' feedback helpful as you consider how to proceed with this work.

Sincerely,

Melissa Vazquez Hernandez

Associate Editor

PLOS Biology

mvazquezhernandez@plos.org

------------------------------------------------------------------------

REVIEWERS' COMMENTS

------------------------------------------------------------------------

Reviewer #1:

I appreciate the authors' careful consideration in responding to my questions and comments, and their effort in thoroughly revising the manuscript.

------------------------------------------------------------------------

Reviewer #2:

After carefully reviewing the revised manuscript, I must say that while the dataset and overall framing are potentially impactful (and I appreciate the effort of the authors to address the reviewer feedback), several key assumptions remain insufficiently supported. Below I describe my main concerns in more detail.

REBASE cross-validation

While the idea of using the REBASE database of sequenced methylation patterns for confirmation of functionality of predicted RM systems can be useful, the data reported by the authors raise more concerns though.

First, methylation patterns differ among different types of RM systems. While type I and II systems do prevent self cleavage by methylating nearly all of their recognition sites, this is not true for type III and IV. Type III systems methylate only one strand of the DNA at their asymmetric recognition site. Therefore, many (about 50%) predicted sites remain unmethylated. Moreover, type IV systems work in an oposite way - restriction endonucleases cleave only methylated DNA, thus their recognition site must be unmethylated!

The pooling of all the RM system types into one category and expecting that their functionality is confirmed by methylation thus seems like a questinable assumption. Type I and II with only 50% sites methylated (selected cutoff in their analysis) would most likely lead to self cleavage, while methylation of 50% or more in type IV would also lead to self cleavage, pointing towards pseduogenised REases. Only in the case of type III systems this value would be a reasonable expectation.

The data presented by the authors question their approach even more. Even when pooled together, one would expect that in clades with higher proportion of type III and IV systems, the ratio of detected RM systems to the count of modification patterns, should be higher (fewer modification patterns) than in clades with predominant type I and II (more modification patterns). However, the data show the oposite. The largest RM:modification-pattern ratio is Neisseria gonorrhoeae (2.11 filtered), yet in their main Table 1, N. gonorrhoeae has 0 type IV systems. Conversely, species with very large type IV totals in the main dataset (e.g., S. aureus, S. enterica) show ratios < 1 in the REBASE subset (filtered 0.504 and 0.833, respectively), i.e., more methylation patterns than RM calls, not the inflation one would expect from abundant type IV systems.

Moreover, the major type IV outlier in the main dataset, Mycobacterium tuberculosis (they state >50% of all type IV systems are in M. tuberculosis; Table 1) is not included in the REBASE validation table even though many sequence isolates with annotated RM methylation patterns are present in REBASE. Thus, the validation does not test the regime where type IV inflation/decoupling from methylation should be most acute. Instead, Table S1 has E. coli, which is not included in the main bioinformatic analysis. Such selection is rather puzzling and does not imply a very rigorous approach.

High variance in the RM-systems : methylation-patterns ratio also sharply limits what that test can legitimately validate. The filtered ratios range from 0.504 (S. aureus) to 2.110 (N. gonorrhoeae) (∼4-fold range), with similarly broad unfiltered ratios (0.236-1.25). That alone implies that even if the grand mean ratio is near 1 (the authors report 0.909 when filtering motifs by arbitrary >50% methylation) any inference about functionality is highly heterogeneous across taxa.

Such heterogeneity can be, apart from detection biases (insensitivity of PacBio SMRT sequencing to 5mC methylation), also caused by non-RM-related methylation, e.g., immunity related BREX/DISARM/MADS-like MTases and regulatory methylation signals (Dam, CcrM, Dcm, Phase-variable Type III Mod MTases etc…).

Taken together, I think one can make use of the curated REBASE PacBio database for confirmation of functionality of the predicted RM systems but with substantially more focus on the details mentioned above. The MTases there are classified based on the type, percentage of methylated sites, the recognition sequence and the type of modification.

From that data one can exclude for example Dam and Dcm MTases, which are well annotated there but cannot be included as a proxy for REase activity. Also, for such analysis I would appreciate if the authors could report (i) a per-genome scatterplot of RM count vs methylation-pattern count with a correlation (Spearman is fine), (ii) a comparison of the two measurement methods using Bland-Altman-style difference plot, and (iii) the analysis stratified by RM types), because type IV is expected to decouple from MTase-linked methylation signatures.

Another observation which is puzzling to me, is that in the REBASE cross-validation, Table S1 reveals that several species are supported by very small numbers of methylome-profiled genomes (e.g., 4 S. pyogenes, 6 E. faecium). The manuscript states that the authors used 'all publicly available genomes' for which 'complete motif modification profiles' could be obtained from REBASE (584 genomes total), but it does not specify the practical filters that lead to such limited per-species coverage (e.g., requirement for closed/RefSeq-complete assemblies, accession matching, or specific SMRT outputs), nor provide an accession list and download date to allow readers to verify completeness and representativeness. A very brief search showed that for example in S. pyogenes, the REBASE PacBio database contains 55 complete genomes with anotated methylomes, with variable methylation patterns.

Because these small Ns can affect the inferred RM:motif ratios for individual taxa, the authors should explicitly document the REBASE query/criteria, provide a list of included accessions, and discuss how limited and uneven methylome availability impacts the strength of this validation.

Species selection for GLMMs

This detailed examination of the reported measures brought me back to their initialy reported findings. The selection of species for the within-species phylogeny-controlled GLMMs (Fig. 3) currently appears insufficiently justified and potentially prone to selection bias. The authors state that they excluded several taxa because they lacked "heterogeneity in RM system counts," yet their own Table S3 indicates substantial within-species diversity of RM system content (high Shannon indices) in multiple excluded species, which makes it unclear why these taxa were deemed uninformative.

Because Shannon diversity of RM identities need not map onto variance in total RM counts per genome, the appropriate way to support the exclusion criterion would be to report, for all 14 species, the empirical dispersion of RM counts per genome (e.g. a violin/boxplot) and ideally estimate species-specific RM slopes in a hierarchical (partial-pooling) model rather than presenting results for a pre-selected subset.

Without this, it remains possible that some excluded species would show null or even opposite within-species associations, and the scope of the "within-species" claim should be restricted to solely the five species that were actually analyzed.

Varying RM efficacy

I appreciate that the authors performed a model robustness analysis by varying RM efficacy (i.e., the assumed strength of RM-mediated protection during HGT) and showed that the qualitative association of interest is stable across a range of efficacies (the new supplemental analysis/figure). This also directly addresses my concern about unmodelled anti-restriction/anti-RM mechanisms: while they do not explicitly model particular anti-RM genes or pathways, sweeping the efficacy parameter effectively captures the net impact of partial RM evasion or inhibition and tests whether the conclusions depend on near-perfect restriction.

Trait depth inference

The authors' defense of trait depth is based on the assumption that (i) extensive HGT sufficient to make a trait appear "deep" (>0.9 clade positivity) would also tend to disseminate the trait beyond that clade, and (ii) recombination should blur RM and ARG signals in broadly comparable ways.

Both assumptions are questionable given empirical evidence that gene flow can be highly structured, with exchange occurring far more frequently within biologically coherent populations than between them, producing sharp gene-flow-defined clusters even among close relatives. (https://doi.org/10.1016/j.cell.2019.06.033)

In such a regime, a horizontally acquired trait can plausibly sweep to near-fixation within a population/clade (meeting the ≥0.9 criterion) while remaining rare outside it, so deep trait depth does not necessarily indicate old/vertical inheritance.

Moreover, phage-defense repertoires can turn over extremely rapidly via mobile genetic elements, thus effectively decoupling defense presence from deeper phylogenetic structure and making within-clade saturation compatible with recent gain and fast turnover. (https://doi.org/10.1126/science.abb1083).

Together, these observations imply that trait depth may primarily reflect population structure and within-population sweeps rather than acquisition timing, and that differential mobility/turnover of RM-linked defenses versus ARGs could systematically bias the intended "recent vs deep" comparisons.

Overall assessment

Overall, while the revised manuscript addresses some technical points, the revised analyses and responses introduce (and in several places leave unresolved) substantial concerns about the biological interpretation and the validity of key inferential steps. Most notably the use of methylome patterns as support for RM functionality, the ad hoc species selection for within-species effect estimation, and the reliance on trait depth as a proxy for temporal depth under structured gene flow and rapid defense turnover.

At the end of the day, these discrepancies undermine confidence in the central claims, and I therefore cannot recommend the article for publication in its current form. While I still find the idea of using eco-evolutionary dynamics and mathematical modelling to interpret patterns in HGT compelling, resolving the resulting doubts would require extensive additional analyses and reframing beyond what I would consider feasible as a minor or moderate revision. For this reason, I refrain from commenting on the authors' other responses.

# Author comment: Author response to Decision Letter 3

**DOI:** 10.1371/journal.pbio.3003842.r005

4 Mar 2026

Attachment Submitted filename: Responses_to_reviewers_auresp_3.docx

# Editor report: Decision Letter 3

**DOI:** 10.1371/journal.pbio.3003842.r006

**Authors:** Melissa Vazquez Hernandez, Melissa Vazquez Hernandez, Melissa Vazquez Hernandez, Melissa Vazquez Hernandez

9 Mar 2026

Dear Edze and Joe,

Thank you for your patience while we considered your appeal of the manuscript "Eco-evolutionary feedbacks drive the co-occurrence of restriction-modification systems and antimicrobial resistance genes" for consideration as a Research Article at PLOS Biology.

As mentioned in the appeal yes letter, we would like you to revise according to your appeal letter and submit a response to reviewers addressing the concerns of Reviewer 2. We will then send this to Reviewer 1 to seek their opinion, and only if needed maybe an additional reviewer. Please feel free to reach out if something is unclear.

In addition to these revisions, you may need to complete some formatting changes, which you will receive in a follow up email. A member of our team will be in touch with a set of requests shortly. If you do not receive a separate email within a few days, please assume that checks have been completed, and no additional changes are required.

We expect to receive your revised manuscript within 1 month. Please email us (plosbiology@plos.org) if you have any questions or concerns, or would like to request an extension.

At this stage, your manuscript remains formally under active consideration at our journal; please notify us by email if you do not intend to submit a revision so that we withdraw the manuscript.

**IMPORTANT - SUBMITTING YOUR REVISION**

Your revisions should address the specific points made by each reviewer. Please submit the following files along with your revised manuscript:

1. A 'Response to Reviewers' file - this should detail your responses to the editorial requests, present a point-by-point response to all of the reviewers' comments, and indicate the changes made to the manuscript.

*NOTE: In your point-by-point response to the reviewers, please provide the full context of each review. Do not selectively quote paragraphs or sentences to reply to. The entire set of reviewer comments should be present in full and each specific point should be responded to individually.

You should also cite any additional relevant literature that has been published since the original submission and mention any additional citations in your response.

2. In addition to a clean copy of the manuscript, please also upload a 'track-changes' version of your manuscript that specifies the edits made. This should be uploaded as a "Revised Article with Changes Highlighted " file type.

*Resubmission Checklist*

When you are ready to resubmit your revised manuscript, please refer to this resubmission checklist: https://plos.io/Biology_Checklist

To submit a revised version of your manuscript, please go to https://www.editorialmanager.com/pbiology/ and log in as an Author. Click the link labelled 'Submissions Needing Revision' where you will find your submission record.

Please make sure to read the following important policies and guidelines while preparing your revision:

*Published Peer Review*

Please note while forming your response, if your article is accepted, you may have the opportunity to make the peer review history publicly available. The record will include editor decision letters (with reviews) and your responses to reviewer comments. If eligible, we will contact you to opt in or out. Please see here for more details:

https://blogs.plos.org/plos/2019/05/plos-journals-now-open-for-published-peer-review/

*PLOS Data Policy*

Please note that as a condition of publication PLOS' data policy (http://journals.plos.org/plosbiology/s/data-availability) requires that you make available all data used to draw the conclusions arrived at in your manuscript. If you have not already done so, you must include any data used in your manuscript either in appropriate repositories, within the body of the manuscript, or as supporting information (N.B. this includes any numerical values that were used to generate graphs, histograms etc.). For an example see here: http://www.plosbiology.org/article/info%3Adoi%2F10.1371%2Fjournal.pbio.1001908#s5

*Blot and Gel Data Policy*

We require the original, uncropped and minimally adjusted images supporting all blot and gel results reported in an article's figures or Supporting Information files. We will require these files before a manuscript can be accepted so please prepare them now, if you have not already uploaded them. Please carefully read our guidelines for how to prepare and upload this data: https://journals.plos.org/plosbiology/s/figures#loc-blot-and-gel-reporting-requirements

*Protocols deposition*

To enhance the reproducibility of your results, we recommend that if applicable you deposit your laboratory protocols in protocols.io, where a protocol can be assigned its own identifier (DOI) such that it can be cited independently in the future. Additionally, PLOS ONE offers an option for publishing peer-reviewed Lab Protocol articles, which describe protocols hosted on protocols.io. Read more information on sharing protocols at https://plos.org/protocols?utm_medium=editorial-email&utm_source=authorletters&utm_campaign=protocols

Thank you again for your submission to our journal. We hope that our editorial process has been constructive thus far, and we welcome your feedback at any time. Please don't hesitate to contact us if you have any questions or comments.

Sincerely,

Melissa

Melissa Vazquez Hernandez, Ph.D.

Associate Editor

PLOS Biology

mvazquezhernandez@plos.org

# Author comment: Author response to Decision Letter 4

**DOI:** 10.1371/journal.pbio.3003842.r007

15 Apr 2026

Attachment Submitted filename: Responses_to_reviewers_resubmission_2.docx

# Peer review: Decision Letter 4

**DOI:** 10.1371/journal.pbio.3003842.r008

**Authors:** Melissa Vazquez Hernandez, Melissa Vazquez Hernandez, Melissa Vazquez Hernandez, Melissa Vazquez Hernandez, Melissa Vazquez Hernandez

27 Apr 2026

Dear Joe,

Thank you for your patience while we considered your revised manuscript "Eco-evolutionary feedbacks drive the co-occurrence of restriction-modification systems and antimicrobial resistance genes" for publication as a Research Article at PLOS Biology. This revised version of your manuscript has been evaluated by the PLOS Biology editors, the Academic Editor and Reviewer 1.

Based on the reviews, we are likely to accept this manuscript for publication, provided you satisfactorily address the remaining points raised by the reviewers regarding the limitation on trait depth. Please also make sure to address the following data and other policy-related requests.

1) We routinely suggest changes to titles to ensure maximum accessibility for a broad, non-specialist readership, and to ensure they reflect the contents of the paper. In this case, we would suggest a minor edit to the title, which is just to add "bacteria". Please ensure you change both the manuscript file and the online submission system, as they need to match for final acceptance:

"Eco-evolutionary feedbacks drive the co-occurrence of restriction-modification systems and antimicrobial resistance genes in bacteria"

2) You may be aware of the PLOS Data Policy, which requires that all data be made available without restriction: http://journals.plos.org/plosbiology/s/data-availability. For more information, please also see this editorial: http://dx.doi.org/10.1371/journal.pbio.1001797

Please supply the numerical values either in the a supplementary file or as a permanent DOI’d deposition for the following figures:

Figure 1AB, 2AB, 3, S1AB, S1.2AB, S3A-D, S6, S7, S8

NOTE: the numerical data provided should include all replicates AND the way in which the plotted mean and errors were derived (it should not present only the mean/average values).

*I am aware that your data availability statement says "Data collected on these genomes and all scripts used for mathematical modelling, bioinformatic data collection, and statistical analyses can be accessed at https://doi.org/10.5281/zenodo.19387437". Could you please confirm that all data and scripts necessary to reproduce these figures are there? It would be great, albeit not exactly necessary, if you could indicate which data/code is for which figure.

3) Please cite the location of the data clearly in all relevant main and supplementary Figure legends, e.g. “The data underlying this Figure can be found in S1 Data” or “The data underlying this Figure can be found in https://doi.org/10.5281/zenodo.XXXXX”

4) Please provide the tree files for the phylogenetic trees in Figures S4.1, S4.2, S4.3, S4.4, S4.5. Please make sure all relevant figures have scale bars.

5) Per journal policy, if you have generated any custom code during the course of this investigation, please make it available without restrictions. Please ensure that the code is sufficiently well documented and reusable, and that your Data Statement in the Editorial Manager submission system accurately describes where your code can be found. More information on our Code Policy, what and how to share can be found here: https://journals.plos.org/plosbiology/s/code-availability

Please note that we cannot accept sole deposition of code in GitHub, as this could be changed after publication. However, you can archive this version of your publicly available GitHub code to Zenodo. Once you do this, it will generate a DOI number, which you will need to provide in the Data Accessibility Statement (you are welcome to also provide the GitHub access information). See the process for doing this here: https://docs.github.com/en/repositories/archiving-a-github-repository/referencing-and-citing-content

As you address these items, please take this last chance to review your reference list to ensure that it is complete and correct. If you have cited papers that have been retracted, please include the rationale for doing so in the manuscript text, or remove these references and replace them with relevant current references. Any changes to the reference list should be mentioned in the cover letter that accompanies your revised manuscript.

In addition to these revisions, you may need to complete some formatting changes, which you will receive in a follow up email. A member of our team will be in touch with a set of requests shortly. If you do not receive a separate email within a few days, please assume that checks have been completed, and no additional changes are required.

We expect to receive your revised manuscript within two weeks.

To submit your revision, please go to https://www.editorialmanager.com/pbiology/ and log in as an Author. Click the link labelled 'Submissions Needing Revision' to find your submission record. Your revised submission must include the following:

- a cover letter that should detail your responses to any editorial requests, if applicable, and whether changes have been made to the reference list

- a Response to Reviewers file that provides a detailed response to the reviewers' comments (if applicable, if not applicable please do not delete your existing 'Response to Reviewers' file.)

- a track-changes file indicating any changes that you have made to the manuscript.

NOTE: If Supporting Information files are included with your article, note that these are not copyedited and will be published as they are submitted. Please ensure that these files are legible and of high quality (at least 300 dpi) in an easily accessible file format. For this reason, please be aware that any references listed in an SI file will not be indexed. For more information, see our Supporting Information guidelines:

https://journals.plos.org/plosbiology/s/supporting-information

*Published Peer Review History*

Please note that you may have the opportunity to make the peer review history publicly available. The record will include editor decision letters (with reviews) and your responses to reviewer comments. If eligible, we will contact you to opt in or out. Please see here for more details:

https://plos.org/published-peer-review-history/

*Press*

Should you, your institution's press office or the journal office choose to press release your paper, please ensure you have opted out of Early Article Posting on the submission form. We ask that you notify us as soon as possible if you or your institution is planning to press release the article.

*Protocols deposition*

To enhance the reproducibility of your results, we recommend that if applicable you deposit your laboratory protocols in protocols.io, where a protocol can be assigned its own identifier (DOI) such that it can be cited independently in the future. Additionally, PLOS ONE offers an option for publishing peer-reviewed Lab Protocol articles, which describe protocols hosted on protocols.io. Read more information on sharing protocols at https://plos.org/protocols?utm_medium=editorial-email&utm_source=authorletters&utm_campaign=protocols

Please do not hesitate to contact me should you have any questions.

Sincerely,

Melissa

Melissa Vazquez Hernandez, Ph.D.

Associate Editor

mvazquezhernandez@plos.org

PLOS Biology

------------------------------------------------------------------------

REVIEWER'S COMMENTS

------------------------------------------------------------------------

Reviewer #1:

On request of the Editors, I have re-evaluated the manuscript to determine whether the revised version and the Authors' responses adequately address the concerns raised by Reviewer 2, which is necessary for the manuscript to be considered for publication.

With respect to the concerns regarding the use of methylome patterns as evidence for restriction-modification (R-M) system functionality and the species selection procedure, I find that the Authors have provided additional explanations, supporting analyses, and discussion of limitations that strengthen their methodological approach and support their results. Although I agree with Reviewer 2 that factors such as orphan methyltransferases, phase-variable expression, lack of direct evidence for the presence of restriction enzymes, and limitations of REBASE may introduce errors, the study's objective—to infer general principles from a large dataset—does not require maximal precision in system selection. Furthermore, the analytical permutations introduced by the Authors (e.g., exclusion of Type IV systems and variation of cutoff values) did not substantially affect the results, indicating robustness to the specific issues raised by Reviewer 2. In this regard, I consider the methodological details to be sufficiently justified and the analyses reproducible.

However, the uncertainty regarding whether trait depth accurately reflects acquisition timing remains a valid concern, particularly because the results depend strongly on this assumption. Nevertheless, based on the results presented and the supporting literature, I do not find sufficient grounds to reject the Authors' hypothesis. Namely, this issue constitutes a hypothesis in its own right that would require independent testing; therefore, I tried to base my decision on whether the Authors have provided adequate support for their current hypothesis. Accordingly, although some uncertainty remains regarding the study design and results, I consider that the manuscript presents findings of sufficient interest and relevance—particularly in light of the further questions it raises—to warrant publication.

# Author comment: Author response to Decision Letter 5

**DOI:** 10.1371/journal.pbio.3003842.r009

20 May 2026

Attachment Submitted filename: Responses_to_reviewers_resubmission_2_auresp_5.docx

# Editor report: Decision Letter 5

**DOI:** 10.1371/journal.pbio.3003842.r010

**Authors:** Melissa Vazquez Hernandez, Melissa Vazquez Hernandez, Melissa Vazquez Hernandez, Melissa Vazquez Hernandez, Melissa Vazquez Hernandez, Melissa Vazquez Hernandez

24 May 2026

Dear Joe, dear Edze,

Thank you for the submission of your revised Research Article "Eco-evolutionary feedbacks drive the co-occurrence of restriction-modification systems and antimicrobial resistance genes in bacteria" for publication in PLOS Biology. On behalf of my colleagues and the Academic Editor, Kathryn E. Holt, I am pleased to say that we can in principle accept your manuscript for publication, provided you address any remaining formatting and reporting issues. These will be detailed in an email you should receive within 2-3 business days from our colleagues in the journal operations team; no action is required from you until then. Please note that we will not be able to formally accept your manuscript and schedule it for publication until you have completed any requested changes.

Please take a minute to log into Editorial Manager at http://www.editorialmanager.com/pbiology/, click the "Update My Information" link at the top of the page, and update your user information to ensure an efficient production process.

PRESS

We frequently collaborate with press offices. If your institution or institutions have a press office, please notify them about your upcoming paper at this point, to enable them to help maximise its impact. If the press office is planning to promote your findings, we would be grateful if they could coordinate with biologypress@plos.org. If you have previously opted in to the early version process, we ask that you notify us immediately of any press plans so that we may opt out on your behalf.

We also ask that you take this opportunity to read our Embargo Policy regarding the discussion, promotion and media coverage of work that is yet to be published by PLOS. As your manuscript is not yet published, it is bound by the conditions of our Embargo Policy. Please be aware that this policy is in place both to ensure that any press coverage of your article is fully substantiated and to provide a direct link between such coverage and the published work. For full details of our Embargo Policy, please visit http://www.plos.org/about/media-inquiries/embargo-policy/.

Thank you again for choosing PLOS Biology for publication and supporting Open Access publishing. We look forward to publishing your study.

Sincerely,

Melissa

Melissa Vazquez Hernandez, Ph.D., Ph.D.

Associate Editor

PLOS Biology

mvazquezhernandez@plos.org
